# Alternate RASSF1 Transcripts Control SRC Activity, E-Cadherin Contacts, and YAP-Mediated Invasion

**DOI:** 10.1016/j.cub.2015.09.072

**Published:** 2015-12-07

**Authors:** Nikola Vlahov, Simon Scrace, Manuel Sarmiento Soto, Anna M. Grawenda, Leanne Bradley, Daniela Pankova, Angelos Papaspyropoulos, Karen S. Yee, Francesca Buffa, Colin R. Goding, Paul Timpson, Nicola Sibson, Eric O’Neill

**Affiliations:** 1 CRUK/MRC Oxford Institute, Department of Oncology, University of Oxford, Oxford OX3 7DQ, UK; 2 Applied Computational Genomics Group, Department of Oncology, University of Oxford, Oxford OX3 7DQ, UK; 3 Faculty of Medicine, Garvan Institute of Medical Research, University of New South Wales, Darlinghurst, NSW 2010, Australia; 4 Ludwig Institute for Cancer Research, University of Oxford, Oxford OX3 7DQ, UK

## Abstract

Tumor progression to invasive carcinoma is associated with activation of SRC family kinase (SRC, YES, FYN) activity and loss of cellular cohesion. The hippo pathway-regulated cofactor YAP1 supports the tumorigenicity of RAS mutations but requires both inactivation of hippo signaling and YES-mediated phosphorylation of YAP1 for oncogenic activity. Exactly how SRC kinases are activated and hippo signaling is lost in sporadic human malignancies remains unknown. Here, we provide evidence that hippo-mediated inhibition of YAP1 is lost upon promoter methylation of the RAS effector and hippo kinase scaffold RASSF1A. We find that RASSF1A promoter methylation reduces YAP phospho-S127, which derepresses YAP1, and actively supports YAP1 activation by switching *RASSF1* transcription to the independently transcribed RASSF1C isoform that promotes Tyr kinase activity. Using affinity proteomics, proximity ligation, and real-time molecular visualization, we find that RASSF1C targets SRC/YES to epithelial cell-cell junctions and promotes tyrosine phosphorylation of E-cadherin, β-catenin, and YAP1. RASSF1A restricts SRC activity, preventing motility, invasion, and tumorigenesis in vitro and in vivo, with epigenetic inactivation correlating with increased inhibitory pY527-SRC in breast tumors. These data imply that distinct *RASSF1* isoforms have opposing functions, which provide a biomarker for YAP1 activation and explain correlations of *RASSF1* methylation with advanced invasive disease in humans. The ablation of epithelial integrity together with subsequent YAP1 nuclear localization allows transcriptional activation of β-catenin/TBX-YAP/TEAD target genes, including Myc, and an invasive phenotype. These findings define gene transcript switching as a tumor suppressor mechanism under epigenetic control.

## Highlights

• Methylation of RASSF1A correlates with loss of hippo-inhibitory phospho-S127-YAP1
• SRC, FYN, and YES are activated by RASSF1C in RASSF1A-methylated cells
• RASSF1C promotes E-cadherin internalization and reduces cell junction integrity
• RASSF1A loss drives RASSF1C-YAP1/β-catenin-mediated transcription and invasion

## Graphical Abstract

**Figure:**
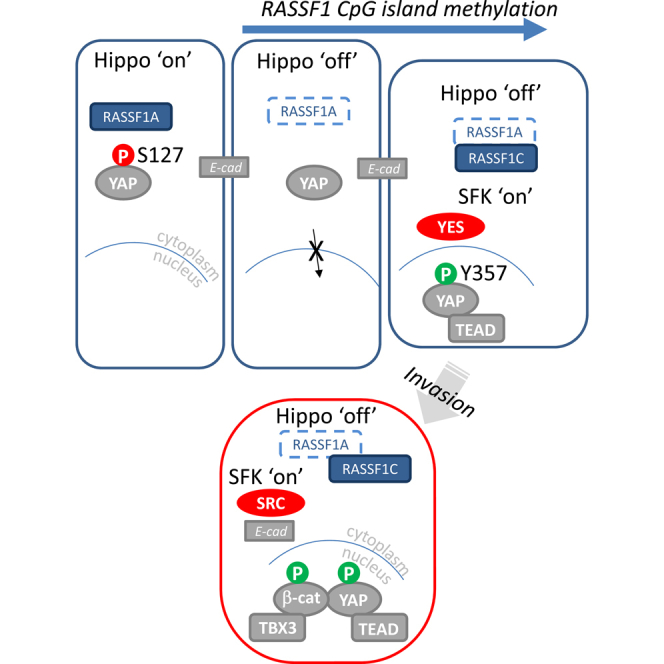

## Teaser

In a wide variety of sporadic malignancies, promoter methylation of the *RASSF1* gene is associated with tumor invasion and metastasis. Vlahov et al. show that the clinical phenotype is driven by both RASSF1A loss and the independently transcribed RASSF1C isoform, which promotes SRC activation, pseudo-EMT, and β-catenin/YAP1-dependent invasion of tumor cells.

## Introduction

Recent advances have highlighted that YES-associated protein (YAP1) supports KRAS tumorigenicity and assists in the maintenance of transformed phenotypes [1]. YAP1 drives proliferation by acting as a cofactor for TEAD transcriptional regulators, an activity which is restricted by hippo pathway-mediated disruption of TEAD association. In model systems, genetic ablation of core hippo pathway components leads to increased tumorigenesis [1]. In human tumors, failure to activate LATS1 due to either GNAQ mutations in uveal melanoma or through inactivation of NF2/merlin in the tumor-prone neurofibromatosis syndrome prevent this inhibitory signal and make YAP1 permissive for activation [1]. Similarly, stromal mechanics and genetic instability are reported to trigger the hippo pathway and present a tumor barrier, but as with GNAQ mutations and germline defects in NF2/merlin, these mechanisms appear to be independent of the hippo kinase/MST itself [1]. Identification of the core hippo pathway by proteomics has revealed the main direct activators of MST kinases to be SAV1 and RASSFs [2], which although infrequently mutated in cancers [3] have germline and epigenetic alterations, particularly in RASSF1A, that accelerate tumor onset and increase tumorigenicity [4, 5]. Moreover, RASSF1A activation of the hippo pathway both restricts YAP1 binding to TEAD [6] and is a direct substrate of RAS signaling in the pancreas [7], supporting the potential crosstalk in pancreatic tumor development [8, 9]. Intriguingly, methylation of the CpG island (CGI) spanning the promoter and first exon of *RASSF1* has widespread prognostic value for disease-free and poor overall survival in all major sporadic cancers [10]. Thus, loss of RASSF1A expression in *RASSF1*-methylated tumors is likely to contribute to reduced hippo pathway-mediated repression of YAP1, due to direct activation of MST kinases and the subsequent interaction and activation of LATS1 [4, 11, 12, 13, 14]. This RASSF1A/MST2-promoted LATS1 activity is targeted toward YAP1 [12], resulting in increased pS127-YAP [15] and decreased YAP-TEAD oncogenic behavior [6, 15].

Hahn and colleagues recently highlighted that, by using a YAP1 mutant for all LATS1 phosphorylation sites (5SA) including the inhibitory S127-YAP1 phosphorylation, while permissive, required additional phosphorylation of Y357-YAP1 by the SRC family kinase (SFK) YES to activate transcription [16]. Moreover, they found that the main tumorigenic consequence of APC loss in colorectal cancer was due to a subset of β-catenin genes that were YAP1 dependent. Thus, YAP1 may serve to integrate WNT and RAS signaling in order to trigger epithelial-to-mesenchymal transition (EMT) and invasive disease [17]. Tumor progression to invasive carcinoma is associated with constitutive loss of cellular cohesion, leading to EMT, increased cellular motility, and invasion into the surrounding tissues [18]. Activation of SFKs is associated with increased tumor invasion and metastasis through initial destabilization of epithelial cell-cell junctions. Mechanistically, this occurs through direct phosphorylation of E-cadherin, p120-catenin, and β-catenin [19, 20, 21]. Phosphorylation of E-cadherin promotes internalization, whereas phosphorylation of β-catenin decreases its affinity for E-cadherin, allowing nuclear localization where it serves as a transcriptional co-activator of TCF/Lef1 factors [22, 23]. The identification of a YAP1-β-catenin transcriptional program specified a novel role for β-catenin in activating transcription via T-box factors rather than TCF, particularly in cancers [16]. Importantly, YAP1 has also been shown to cooperate with β-catenin at TEAD-dependent promoters to induce cell proliferation in the heart and during tumorigenesis [24].

Here, we identify that *RASSF1* CGI methylation correlates with hippo pathway inactivation and loss of pS127-YAP1 in sporadic malignancies and provide evidence for the association of this methylation with invasive behavior in cancers, such as breast, bladder, and in gliomas. CGI methylation restricts primary transcript expression but can additionally promote switching to alternative gene promoters or influence splicing, implying that epigenetic regulation may modulate the relative levels of gene isoforms rather than simply silence gene expression [25]. In tumors with *RASSF1* CGI methylation, an alternative isoform, RASSF1C, is expressed from an internal promoter and has been suggested to promote motility and invasive disease [26, 27]. We find that RASSF1C actually supports tumorigenesis by promoting SRC/YES-mediated phosphorylation of E-cadherin, β-catenin, and YAP1, disrupting cell-cell contacts and initiating an EMT-like response. RASSF1A also binds to SFKs but additionally scaffolds CSK, potentially through an exon 1α-encoded C1 domain, not present in RASSF1C, which inhibits SFKs and maintains epithelial integrity. In the absence of RASSF1A, RASSF1C promotes tyrosine phosphorylation of β-catenin and YAP1, resulting in their re-localization to the nucleus and transcriptional activation of the TBX target genes, BCL2L1, BIRC5 [16], and cMyc [28]. Analyses of invasive breast tumor data sets indicate an inverse correlation of high RASSF1A methylation/low pS127-YAP1 with SRC activation and the expression of invasion-associated transcripts. To validate a role in motility and invasion, we demonstrate that RASSF1C directly promotes SFK-dependent motility, 3D invasion of mammospheres and tumor spread in vivo. These data imply that SFK activation/inactivation relies on distinct RASSF1 isoforms, presenting a mechanism for YAP1 activation in sporadic tumors and explains the clinical correlation of *RASSF1* methylation with advanced invasive disease.

## Results

### Switching of RASSF1 Isoforms Induces Nuclear Localization of YAP1

**Figure 1:**
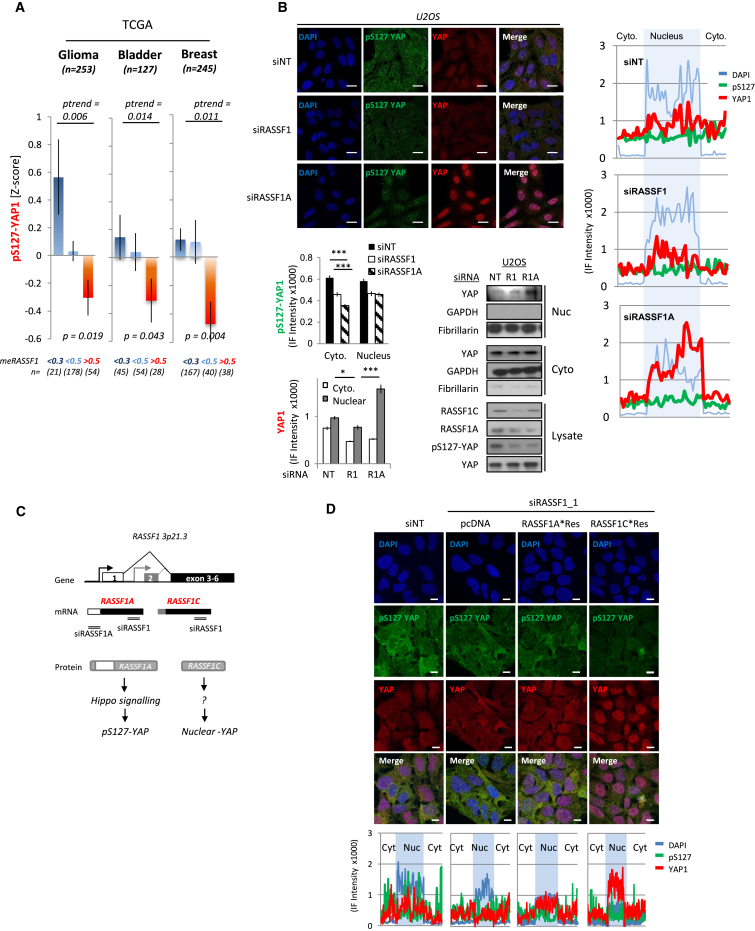
Loss of RASSF1A Mediates Nuclear Localization of YAP1 (A) Correlation of RASSF1 methylation levels (meRASSF1) below 0.3, between 0.3 and 0.5, and above 0.5 with loss of inhibitory YAP1-phospho-Ser127 (pS127-YAP1) in the cancer genome atlas (TCGA) data sets (cBioportal). (B) Immunofluorescence detection of YAP1 and pS127-YAP1 in U2OS cells transfected with siNT, siRASSF1A, and siRASSF1 (top). (Bottom left) Using vectors depicted in Figure S1, histogram indicating the comparative nuclear and cytoplasmic levels of YAP1 (bottom) and pS127 (top) determined by intensity of the immunofluorescence distribution is shown. (Bottom right) The corresponding immunoblot showing the nuclear, cytoplasmic, and total lysate levels of YAP1 is shown. (Far right) Distribution of DAPI (blue), YAP1 (red), and pS127-YAP1 (green) across equatorial cell vector determined by immunofluorescence is shown (average of n = 29). (C) Schematic representation of the RASSF1A and RASSF1C isoforms (top), the location of the sequence for the siRNAs used (middle), and protein functions (bottom). (D) Immunofluorescence detection of YAP1 and pS127-YAP1 in U2OS cells transfected with siNT, siRASSF1, and siRNA-resistant versions of RASSF1A and RASSF1C as indicated. Images are representative of two independent siRASSF1 oligos to which two siRNA restraint isoforms of both RASSF1A and RASSF1C were designed (see Figures S1G and S1H). Distribution of DAPI (blue), YAP1 (red), and pS127-YAP1 (green) across equatorial cell vector determined by immunofluorescence is shown (bottom). All scale bars represent 20 μm.

RASSF1A is a hippo pathway scaffold that switches YAP1 association from oncogenic TEAD transcriptional complexes to tumor-suppressive YAP1/p73 [6]. As RASSF1A expression is lost in multiple cancers and associates with poor outcome, we wanted to determine whether this was a route through which the hippo pathway may be inactivated in sporadic cancers. To address this, we explored YAP1 protein information in data sets of tumors where *RASSF1* is known to be methylated, clinically significant [10], and for which pS127-YAP information was available. We found that methylation of *RASSF1-1α* (representing gene silencing) significantly correlates with low pS127-YAP1 in glioma, bladder, and breast cancer cohorts (Figure 1A), suggesting that YAP1 may be nuclear and active in *RASSF1*-methylated tumors. To test this correlation, we targeted RASSF1A expression in U2OS cells, unmethylated for RASSF1A, with siRNAs and observed lower pS127-YAP1 in line with the clinical data (Figures S1A and S1C). Intriguingly, in contrast to siRNA targeting exons common to all isoforms (siRASSF1), specific ablation of the RASSF1A isoform resulted in elevated nuclear localization of YAP1, indicating that reduced pS127-YAP1 appears required but insufficient for nuclear localization of YAP1 (Figure 1B). In keeping with a loss of hippo pathway activity, the loss of RASSF1 or RASSF1A led to a decrease in the phosphorylation of the core hippo kinases MST1/2 and LATS1 (Figure S1C) and was conversely increased by overexpression of RASSF1A in both U2OS and H1299 (methylated) cells (Figure S1D). RASSF1C transcripts are often present in RASSF1A-methylated tumors due to expression from a distinct promoter and are susceptible to siRASSF1, but not siRASSF1A (Figures 1C, S1B, and S1C) [27]. To determine whether the RASSF1C transcript was responsible for elevated nuclear YAP1 upon siRASSF1A, we designed derivatives of RASSF1A or RASSF1C to be resistant to the two distinct RASSF1 siRNAs, allowing a direct comparison. RASSF1C expression restored nuclear YAP1 in siRASSF1-transfected cells without affecting the pS127-YAP1 levels, whereas RASSF1A failed to do so (Figures 1D, S1E, S1G, and S1H). Thus, reduced hippo signaling and pS127-YAP levels appear effectively uncoupled from automatic nuclear localization, in keeping with the recently demonstrated requirement for tyrosine phosphorylation to mediate the transition [29]. Intriguingly, phosphotyrosine immunoprecipitates of U2OS cells demonstrate that RASSF1C promotes increased tyrosine phosphorylation of YAP1 (Figure S1F). This indicates that promoter methylation of *RASSF1-1α*, which inhibits RASSF1 isoform A expression, reduces hippo signaling and inhibitory pYAP-S127 but favors the nuclear localization of YAP1 via *RASSF1-1α*-independent transcription of RASSF1 isoform C.

### RASSF1 Isoforms Interact with and Differentially Regulate SFKs

**Figure 2:**
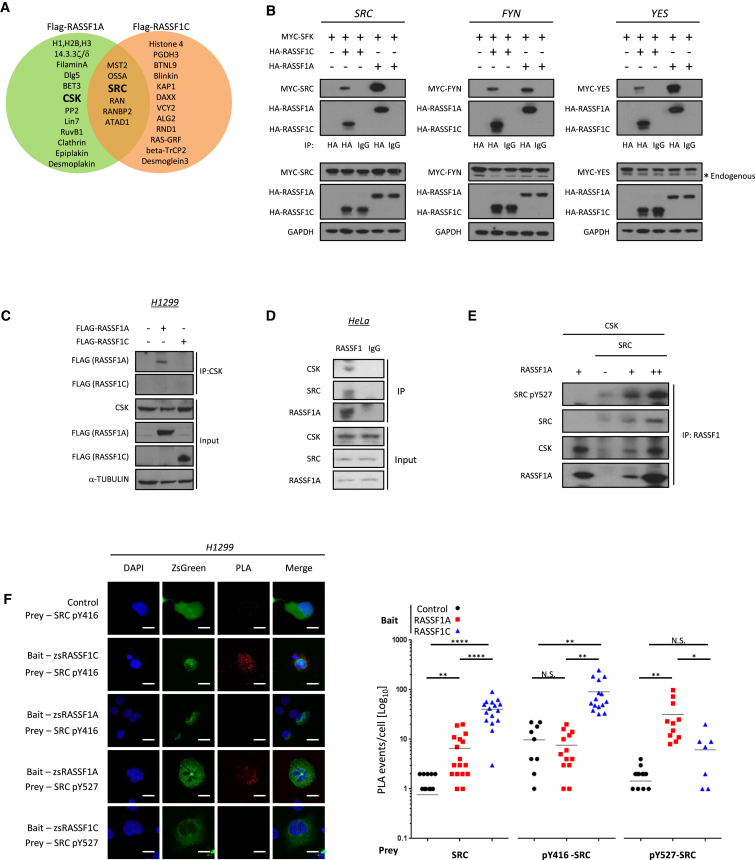
RASSF1C Binds to and Activates SFKs (A) Proteomic identification of RASSF1A and RASSF1C immunoprecipitates by tandem MS/MS. (B) HA-tag immunoprecipitation from 293T cells transfected with MYC-SRC (left), MYC-FYN (middle), or MYC-YES (right) in combination with HA-RASSF1A or HA-RASSF1C, analyzed by immunoblotting. (C) CSK immunoprecipitation from H1299 cells transfected with FLAG-RASSF1A or FLAG-RASSF1C. The precipitates along with input fractions were analyzed by immunoblotting. (D) RASSF1 immunoprecipitation from HeLa cells, immunoblotted for CSK, SRC, and RASSF1A. (E) Purified protein interaction and immunoprecipitation of GST-tagged CSK, SRC, and RASSF1A. (F) Representative immunofluorescence images showing the different levels of colocalization of RASSF1A and RASSF1C (baits) with pY416-SRC and pY527-SRC (preys) using proximity ligation assay (Duolink) in H1299 cells (left). Graph showing the quantification of the events per cell is shown (right). All scale bars represent 20 μm.

To determine the mechanism of how RASSF1C promotes tyrosine phosphorylation and nuclear localization of YAP, we performed a proteomic screen of both isoforms to identify novel protein-protein interactions (Figure 2A). We screened RASSF1 immunoprecipitates for candidates and found that both RASSF1 isoforms bind the tyrosine kinases c-SRC, FYN, and YES (Figure 2B); interestingly, however, RASSF1A had an additional unique association with the SRC inhibitory kinase, CSK (Figures 2C and S2G). The binding of endogenous RASSF1A to CSK was confirmed in HeLa cells, unmethylated for RASSF1A (Figure 2D), and could also be demonstrated to be direct using bacterially purified proteins for RASSF1A, SRC, and CSK (Figure 2E). This association was in line with a similar role for the homolog dRASSF8 in *Drosophila* [30]. To further confirm association, we employed proximity ligation assays and found that, whereas both isoforms bind SRC, RASSF1C associated with active pY416-SRC whereas RASSF1A bound inactive, CSK-phosphorylated, pY527-SRC (Figures 2F and S2A). We next investigated the effect of RASSF1 isoform modulation of SFK activity. Depletion of RASSF1A alone had no effect on SRC activity, whereas siRASSF1 led to reduced pY416-SRC in serum or HGF-stimulated cells (Figures S2B and S2C). Conversely, exogenous expression of RASSF1C elevated pY416-SRC in *RASSF1*α-methylated colorectal, breast, and lung cancer cells but failed to do so in unmethylated cells where RASSF1A is expressed (Figures S2D and S2E) [4]. We therefore hypothesized that RASSF1C may activate SRC but is restricted by competition for association with higher-affinity RASSF1A and associated CSK. To test this, we expressed RASSF1C in H1299 cell lines in which RASSF1A expression was inducible [4] and observed increased pY416-SRC only in the absence of RASSF1A (Figure S2F). Taken together, the data indicate that RASSF1A and RASSF1C modulate SRC activity by promoting differential phosphorylation of SRC.

### RASSF1C Targets SRC to the Plasma Membrane, Destabilizing Junctions

**Figure 3:**
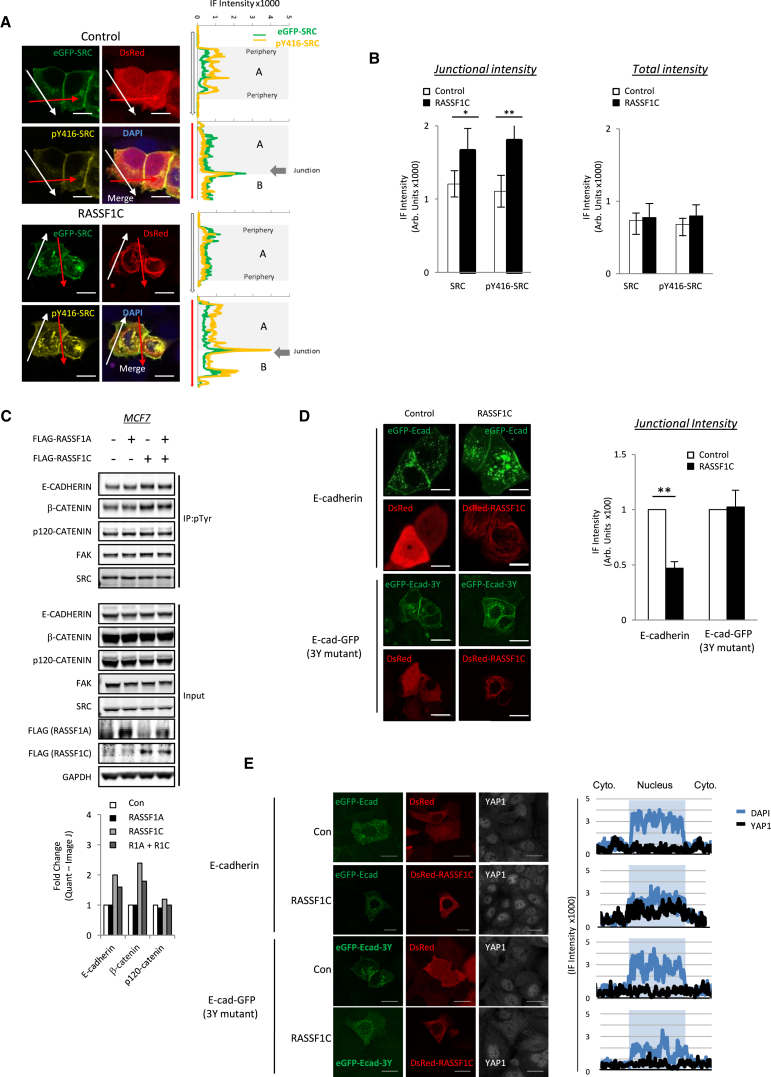
RASSF1C/SRC Destabilizes E-Cadherin at Cell-Cell Junctions (A) Representative immunofluorescence images showing the localization of SRC and pY416-SRC at the sites of cell-cell junctions in control and RASSF1C-expressing H1299 cells. Graphs represent vector line measurements of eGFP-SRC (green) and pY416-SRC immunofluorescence (yellow) through a single cell (A) including bulk membrane (white arrow) or through two attached cells (A and B) including junctional membrane (red arrow). (B) Graphs representing the localization of SRC and pY416-SRC at the sites of cell-cell junctions in control and RASSF1C-expressing cells in (A). Values are mean intensity of the immunofluorescent signal for the indicated antibody and representative of three independent experiments, 15 cells measurements per experiment. (C) Phosphotyrosine immunoprecipitation from MCF7 cells expressing empty vector, FLAG-RASSF1A, FLAG-RASSF1C, or both. Bars indicate quantitation of the displayed images (ImageJ) and are representative of n = 3 experiments. (D) Fluorescence intensity of MCF7 cells expressing GFP-E-cadherin or GFP-E-cadherin mutant (Y753F, Y754F, and Y755F) with either Ds-Red vector or Ds-Red-RASSF1C, quantified in graph (right). Quantification of GFP intensity was done on average of ten cells, three independent experiments. (E) Immunofluorescence detection of YAP1 in MCF7 cells transfected with GFP-E-cadherin or GFP-E-cadherin mutant and either Ds-Red or DsRed-RASSF1C. Representative distribution of DAPI (blue) and YAP1 (black) across equatorial cell vector determined by immunofluorescence is shown (right). All scale bars represent 20 μm.

**Figure 4:**
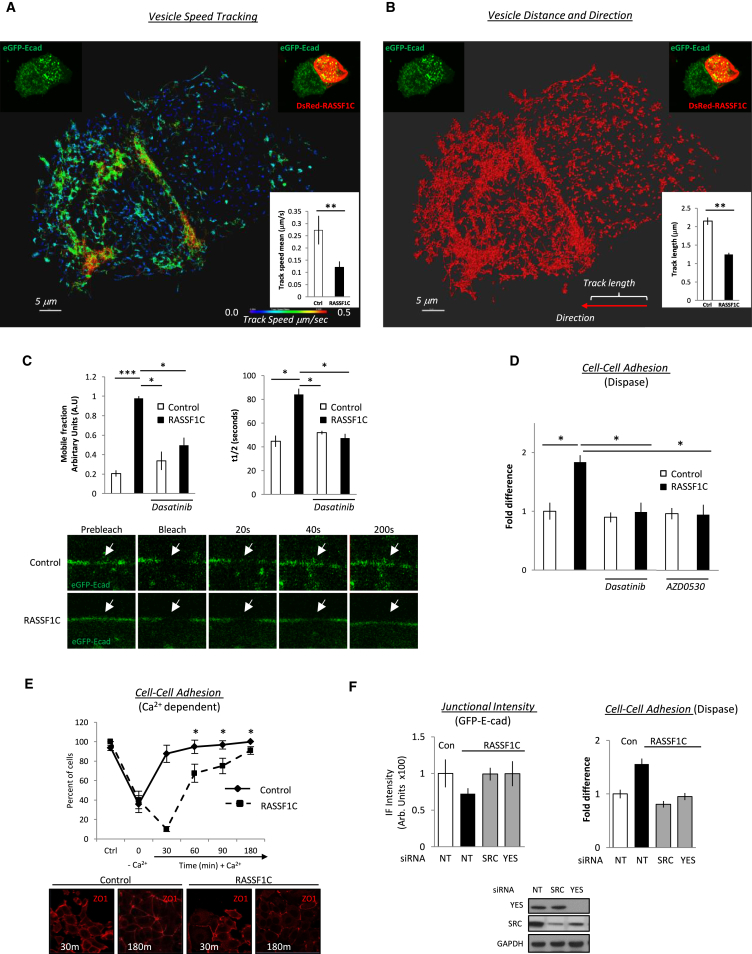
RASSF1C Expression Leads to Disruption of E-Cadherin Trafficking (A and B) Representative images of the tracking of all the vesicles in control and DsRed-RASSF1C-expressing MCF7 cells, transfected with eGFP-E-cadherin (Imaris). Bar graphs (bottom right) show the analysis of the mean speed heatmap (A) or distance (B) of the vesicles in control and RASSF1C cells. For each analysis, five cells per experiment were used and an average of 700 vesicles were tracked (bars). The results are from three independent experiments including Movie S1. Inserts (top) display representative still immunofluorescence images showing the accumulation of E-cadherin and the expression of DsRed-RASSF1C. (C) FRAP analysis of RASSF1C-expressing MCF7 cells. (Left) Mobile fraction of the return of GFP-E-cadherin after photobleaching is shown. (Right) Halftime of the return of GFP-E-cadherin at the sites of cell-cell junctions after bleaching in MCF7 cells is shown. (Bottom) Representative still images of Movies S2 and S3 displaying junctional GFP-E-cadherin in MCF7 cells, expressing DS-Red or Ds-Red-RASSF1C, captured prebleach and following bleach. Arrows, bleached area. For each of the three independent experiments, FRAP analysis was done on ten cells. (D) Quantification of a dispase assay in MCF7 cells expressing Ds-Red or Ds-Red-RASSF1C in the presence or absence of dasatinib treatment (50 nM; 18 hr) or AZD0530 (2.5 μM; 18 hr) showing number of single cells in suspension. (E) Quantification of total cell-cell contacts formed in calcium switch assay in MCF7 cells expressing Zs-green empty vector or Zs-green-RASSF1C. (Bottom) Representative images for ZO-1 at the sites of cell-cell junctions are shown. (F) Quantification of a dispase assay (right) and the junctional intensity levels (left) of MCF7 cells transfected with siNT, siSRC, or siYES. (Bottom) Immunoblot indicating the level of siRNA knockdown is shown.

Once activated, SRC translocates to the cell membrane and phosphorylates key target proteins [31]. To determine whether the activation of SRC by RASSF1C affects the localization, we tracked GFP-SRC and pY416-SRC by florescence microscopy. We found that increased pY416-SRC levels in cells correlated with localization of RASSF1C, endogenous SRC, and pY416-SRC at the membrane (Figures S3A and S3B), with a significant increase at cell junctions compared to non-junctional plasma membrane (Figures 3A and 3B; red versus white arrows). Phosphotyrosine immunoprecipitates suggest that RASSF1C promotes phosphorylation of junction proteins E-cadherin and β-catenin, but not p120-catenin or FAK (focal adhesion kinase) (Figure 3C). SRC phosphorylation of E-cadherin is known to increase its internalization, subsequently destabilizing cell-cell junctions [19]. To determine whether RASSF1C-promoted phosphorylation of E-cadherin has any effect on E-cadherin junctional integrity, we first took GFP-E-cadherin-expressing cells where E-cadherin can be visualized at the cell periphery (Figure 3D). Co-expression of RASSF1C decreased intensity of GFP-E-cadherin at cell-cell junctions compared to controls but did not affect a GFP-E-cadherin derivative harboring mutations in all three SRC phosphorylation sites Y753F, Y754F, and Y755F [32] (Figure 3D). Moreover, the destabilization of eGFP-E-cadherin by RASSF1C occurs in a SRC-dependent manner (Figure S3C). It has been previously shown that loss of junctional components like α-catenin [33, 34] or E-cadherin [35] leads to YAP1 nuclear localization. Therefore, we tested whether the loss of adherens junctions in cells expressing RASSF1C similarly leads to YAP1 nuclear localization and indeed found RASSF1C promotes nuclear YAP1, but not in cells expressing E-cadherin with the SRC phosphorylation sites mutated (Figure 3E). The fidelity of E-cadherin-mediated junctions relies on continuous recycling via internalization and replacement through the late endosomal compartment [36]. Visualization of E-cadherin endosome trafficking in real-time via 4D tracking software (Imaris Bitplane; ANDOR) indicated that RASSF1C-expressing cells had reduced trafficking speed and did not register movement toward the junction, supporting the idea of increased internalization and failure to recycle (Figures 4A and 4B; Movie S1), a phenomenon which again was not observed in the case of the E-cadherin mutant (Figure S4A; Movies S2 and S3). To further determine the E-cadherin stability at cell-cell junctions, we expressed GFP-E-cadherin and monitored its dynamics by FRAP analysis, as has been shown previously [37]. We found that expression of RASSF1C increased the mobile fraction of E-cadherin and its turnover at junctions (increased half-life [t 1/2]), but not upon inhibition of SRC with dasatinib (Figures 4C and S4B; Movies S4 and S5), suggesting that RASSF1C increased E-cadherin recycling via SRC, thus creating junctions that are less molecularly stable. To determine the physical effect on cell-cell adhesion, we employed a dispase assay and observed that RASSF1C expression weakened cellular cohesion in a SRC-dependent manner (Figure 4D). As E-cadherin-mediated adherence relies on Ca^2+^ for stable contacts, we next wanted to test whether cell-cell disruption was indeed due to E-cadherin by employing a Ca^2+^-switch assay. We found that, after E-cadherin contacts were efficiently ablated by removal of Ca^2+^ from the media, RASSF1C-expressing cells failed to efficiently form mature contacts upon Ca^2+^ replenishment (Figures 4E, S5A, and S5B). The siRNA knockdown of both SRC and YES endowed resistance to RASSF1C expression, implicating both in RASSF1C-mediated loss of junctional strength (Figure 4F, right) and E-cadherin intensity (Figure 4F, left). Taken together, the results indicate that expression of RASSF1C destabilizes cell-cell junctions and disrupts further recycling of E-cadherin via SRC. The loss of adherens junctions can have a wide effect on the cell. Therefore, we next wanted to further investigate the effect on cells after the loss of E-cadherin.

### RASSF1C Promotes YAP and β-Catenin-Dependent Transcription

**Figure 5:**
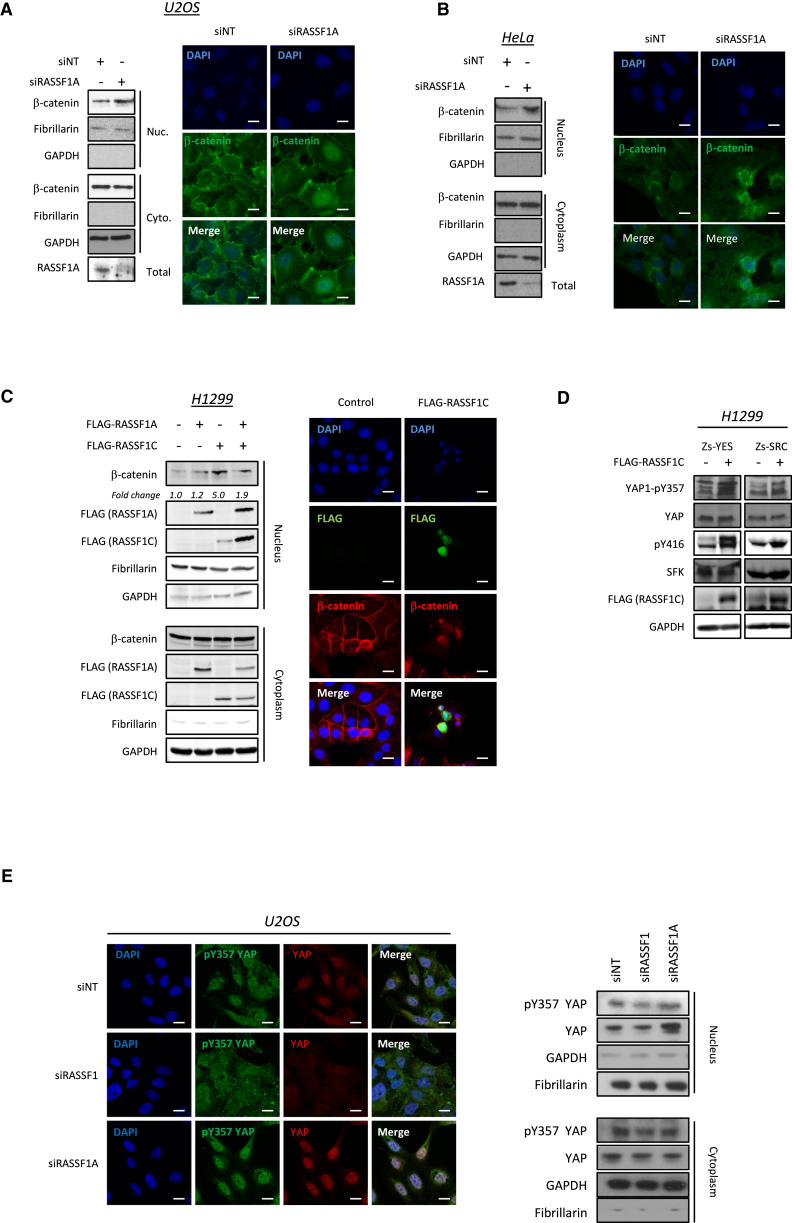
RASSF1C Leads to β-Catenin Nuclear Localization and pY357 (A) Nuclear/cytoplasmic fractionation of U2OS cells transfected with siNT or siRASSF1A (left). Representative images of U2OS cells transfected with siNT or siRASSF1A are shown (right). (B) Nuclear/cytoplasmic fractionation (left) and immunofluorescence detection of β-catenin (right) of HeLa cells transfected with siNT or siRASSF1A. (C) Nuclear/cytoplasmic fractionation of H1299 cells transiently transfected with empty vector, FLAG-RASSF1A, FLAG-RASSF1C, or both FLAG-RASSF1A and FLAG-RASSF1C to show β-catenin localization. (Right) Representative images show β-catenin localization in H1299 transfected with empty vector or FLAG-RASSF1C. (D) H1299 cells transfected with Zs-Green-SRC or Zs-Green-YES and either empty vector or FLAG-RASSF1C and blotted for pY416-SRC, YAP, and the SFK site, pY357-YAP1, as indicated. (E) Immunofluorescence detection of YAP1 and pY357-YAP1 in U2OS cells transfected with siNT, siRASSF1, or siRASSF1A (left) and immunoblot showing the nuclear/cytoplasmic distribution of YAP1 (right). All scale bars represent 20 μm.

**Figure 6:**
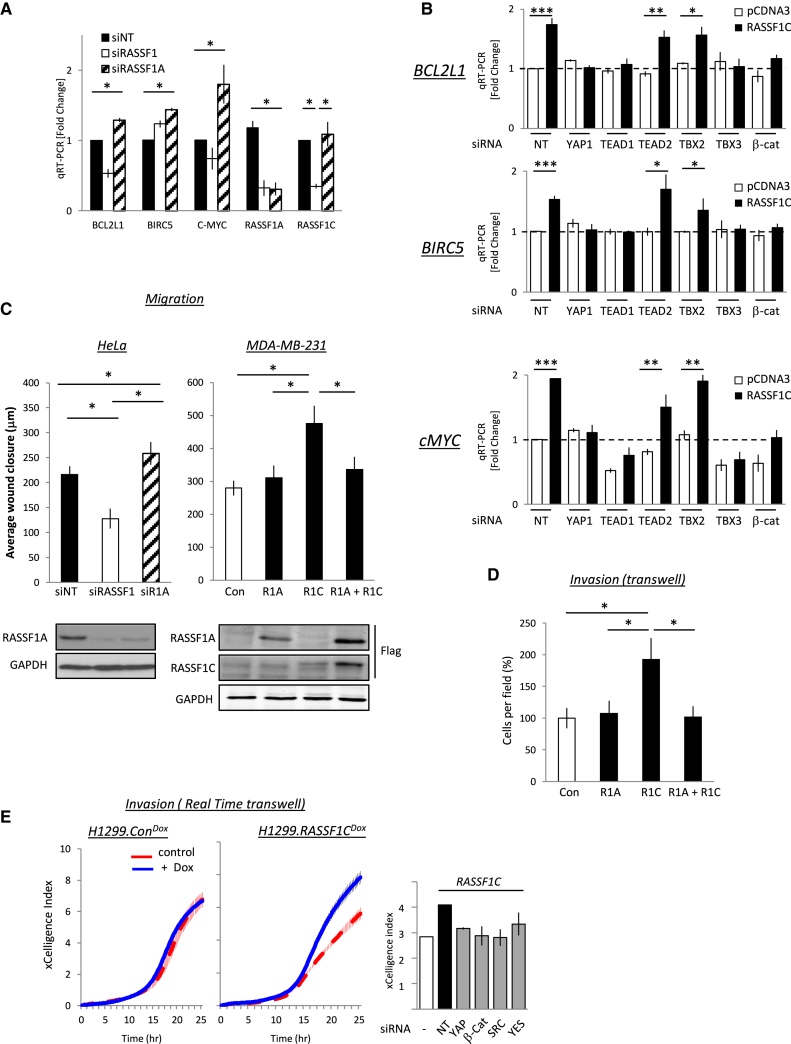
RASSF1C Promotes YAP1-Dependent Transcription and Cell Motility (A) Quantification of qRT-PCR for the β-catenin-YAP target genes *BCL2L1*, *BIRC5*, and *cMYC* as well as RASSF1A and RASSF1C expression in U2OS cells transfected with siNT, siRASSF1, or siRASSF1A. (B) Quantification of qRT-PCR for the β-catenin-YAP target genes *BCL2L1*, *BIRC5*, and *cMYC* in MCF7 cells transfected with empty vector or FLAG-RASSF1C and indicated siRNAs. (C) Scratch wound motility assay of HeLa cells transfected with non-targeting siNT, siRASSF1, and siRASSF1A (left). Wound healing motility assay of MDA-MB-231 cells transfected with empty vector, FLAG-RASSF1A, FLAG-RASSF1C, or both FLAG-RASSF1A and FLAG-RASSF1C is shown (right). (D) Quantification of a Transwell assay with MDA-MB-231 cells expressing empty vector, FLAG-RASSF1A, FLAG-RASSF1C, or both. (E) Migration assay in real time using ExCELLigence analyzer with H1299.R1C^Dox^ TET-ON FLAG-RASSF1C inducible cells (right) or controls H1299.Con^Dox^ TET-ON empty-vector-inducible cells (left) in the presence or absence of 1 μg/μl doxycycline. (Right) Migration assay in real time using ExCELLigence analyzer on MCF7 cells transfected with empty vector or FLAG-RASSF1C with siNT, siYAP1, siβ-catenin, siSRC, or siYES is shown.

Strong E-cadherin contacts require β-catenin, which is protected from degradation through its interaction with E-cadherin [38]. RASSF1C expression promotes the phosphorylation of β-catenin (Figure 3C). Moreover, SRC phosphorylation of β-catenin decreases its affinity for E-cadherin, leading to dissociation from the membrane [22, 23]. This allows nuclear entry and transcription of target genes. Nuclear-cytoplasmic fractionation and immunofluorescence analysis confirmed that either RASSF1C expression (in RASSF1A-methylated H1299 and MCF7 cells) or specific loss of endogenous RASSF1A (U2OS and HeLa cells) increases nuclear β-catenin (Figures 5A–5C, S6A, and S6B). We also found that RASSF1C binds and activates YES1 (Figures 2B and 5D), known to promote YAP1 tyrosine phosphorylation that is required for nuclear localization and accumulation of a β-catenin-YAP complex [16, 39]. RASSF1C-induced nuclear YAP1 is also phosphorylated on Y357 (Figures 5D and 5E). Moreover, in MDA-MB-231 mesenchymal breast cancer cells that lack E-cadherin junctions, expression of RASSF1C is sufficient to drive nuclear localization of β-catenin and YAP1 (Figure S6C). β-catenin/YAP1 utilizes the T-box transcription factor TBX5 to drive a pro-tumorigenic transcriptional program including BCL2L1 and BIRC5 [16]. TBX5 is primarily linked to congenital heart disease; however, the homologs TBX2 and TBX3, which share DNA binding consensus with TBX5, are associated with cancer and invasive cellular behavior [40]. In line with the formation of a YAP1/β-catenin/TBX complex, we found that loss of RASSF1A, or expression of RASSF1C, similarly induced BCL2L1 and BIRC5 (BCL-xl and survivin; Figures 6A, 6B, S6A, and S6D).

Interestingly, RASSF1C also induces *cMYC* (Figures 6A, 6B, and S6A), which is a recognized transcriptional target of β-catenin [41] and YAP/TEAD1 [42] and is bound by TBX3 [28]. Thus, nuclear YAP1/β-catenin may be directed to a set of survival and invasive genes via TEAD1/TBX3 transcription factors. The induction of BCL2L1, BIRC5, and MYC expression by RASSF1C was reduced by siRNAs targeting either β-catenin/TBX3 or YAP1/TEAD1, suggesting that the YAP1/β-catenin complex is recruited via both TBX3 and TEAD1 DNA binding (Figures 6B and S6D). A reduction in BCL2L1, BIRC5, and MYC expression was also observed in RASSF1C cells treated with SRC inhibitors, further implicating that this process is SFK dependent (Figure S6E) [43]. MYC, as with SRC, is associated with invasive growth [44]. In agreement with activation of both oncogenes, RASSF1C promoted motility and invasion and was antagonized by RASSF1A, independently of other SRC activators identified in our screen (Figures 6C, 6D, and S6F–S6J). This was not a defect of attachment or spreading as increased invasion followed induction of RASSF1C expression in attached H1299 cells (Figure 6E, left) and is dependent on β-catenin, YAP, SRC, and YES (Figure 6E, right). Together, the data suggest that loss of RASSF1A promotes invasion via coordinated elevation of pY357-YAP1 and reduced pS127-YAP1, leading to transcriptional activation of specific TEAD/TBX3 genes.

### Expression of RASSF1C Promotes Invasiveness and Tumorigenesis

To investigate the clinical relevance, we interrogated the databases described above (Figure 1A) for correlations between pS127-YAP1^low^ and invasive signatures using total YAP1 levels and the mSigDB database YAP-TAZ signature as a control. We assessed the extent of increased gene expression from invasive, metastatic, or EMT signatures but failed to see any highly significant correlation (p < 0.0001).

**Figure 7:**
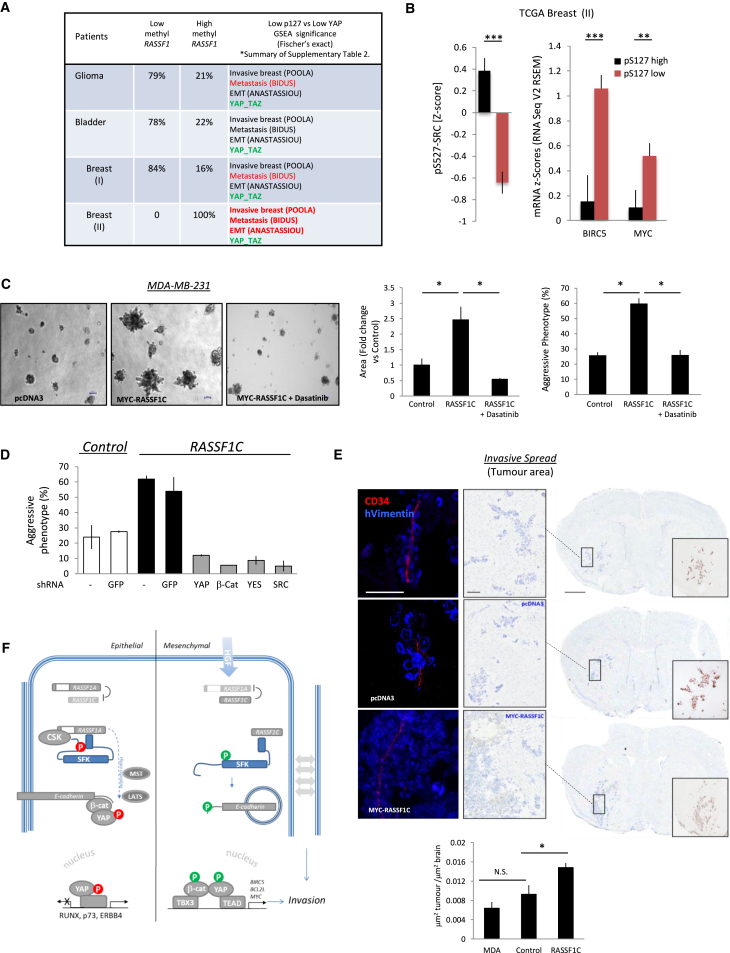
RASSF1C Promotes Invasive Spread and Tumorigenesis In Vivo (A) Cohorts of glioma, bladder, and breast (I, Koboldt; II, invasive carcinoma) from The Cancer Genome Atlas (TCGA) with percent of patients with RASSF1A methylation as indicated. Relative significance of gene changes from MSigDB signatures in active YAP1 is shown (pS127-YAP1^low^ versus YAP1), red (p < 0.5); bold (p < 0.000001); both groups have identical expression of genes for YAP-TAZ signatures (green). (B) The pS127-YAP1^low^ population in the TCGA Breast (II) displays lower pY527-SRC and increased BIRC5 and MYC mRNA (red bars) compared to the pS127-YAP1high population (black bars). (C) Mammospheres from MDA-MB-231 cells stably expressing empty vector, MYC-RASSF1C, or MYC-RASSF1C treated on days 3, 6, and 9 with SFK inhibitor (10 nM dasatinib) grown in Matrigel. Images were taken on day 10. Graphs indicate size of mammospheres (middle) and mammospheres with aggressive phenotypes (right). All scale bars represent 50 μm. (D) Quantification of mammosphere growth experiment on Matrigel, using MDA-MB-231 cells stably expressing empty vector or MYC-RASSF1C and transfected with shGFP, shYAP1, shβ-catenin, shYES, or shSRC. (E) Immunofluorescence photomicrographs showing tumor growth patterns in each of the experimental groups at day 21 after intracerebral tumor cell injection (left). Brain vessels stained with CD34 (red: Cye3 fluorophore) and tumor cells stained with human vimentin (blue: AMCA fluorophore) are shown. The scale bar represents 50 μm. Immunohistochemical photomicrographs of the tumor core for each tumor cell line counterstained with cresyl violet are shown (right). The scale bar represents 1 mm. Higher-resolution images of boxed region are shown on the left-hand side (scale bars 100 μm), and insets show contiguous sections in which tumor cells were stained with anti-vimentin antibody. (Bottom) Graph shows area of tumor colonization in each of the experimental groups 21 days after intracerebral injection. Error bars represent SEM. (F) Model. The hippo pathway both prevents YAP1-TEAD association and promotes cytoplasmic YAP1. RASSF1A sustains CSK inhibitory phosphorylation of SRC family kinases (SFKs) in response to HGF (Figure S2C) and promotes hippo-mediated pS127-YAP1 and tumor suppressive transcription. EMT signals that disrupt RASSF1A or epigenetic inactivation (*meRASSF1*) make YAP1 permissive for TEAD transcription and allow RASSF1C to activate SFKs. Internalization of E-cadherin and phosphorylation of β-catenin and Y357-YAP1 then promotes nuclear localization and TBX/TEAD-mediated transcription of invasive genes invasive transcription.

We next examined a second breast data set where methylation of RASSF1A was confirmed in all cases, suggesting that RASSF1C could be expressed and that loss of pS127-YAP1 can now combine with RASSF1C-promoted pY357-YAP1 to allow YAP1/β-catenin nuclear localization. In this data set, pS127-YAP1^low^ (independent of total-YAP1) did indeed have significantly more genes from invasive, metastatic, and EMT signatures (POOLA p = 1.09e^−78^; BIDUS p = 4.52e^−11^; ANASTASIOU p = 2.62e^−08^; Fisher’s exact test). The fact that the entire data set is from invasive breast cancers negates direct mSigDB analysis in different groups; however, we could control for the increased invasive transcripts in the pS127-YAP1^low^ group as neither the YAP-TAZ or an unrelated signature displayed variation (Figures 7A and S7A; Table S1). We interpret these data in the breast (II) cohort to imply that the pS127-YAP1^low^ group has a YAP/TAZ signature but only an invasive signature when *RASSF1A* methylation is 100% penetrant (Table S2). Interestingly, this group showed significantly lower levels of the CSK substrate site pY527-SRC and elevated levels of both *BIRC5* and *MYC* (Figure 7B). Therefore, as RASSF1C is associated with SRC activation and transcription of these genes, we decided to investigate whether RASSF1C expression could promote tumorigenesis in vitro and in vivo. MDA-MB-231 cells, stably expressing either a control plasmid or RASSF1C, readily formed mammospheres in Matrigel. However, RASSF1C-associated mammospheres were significantly larger and displayed a more-aggressive phenotype that was SRC, YES, β-catenin, and YAP dependent (Figures 7C, 7D, and S7B–S7D). In line with these observations, RASSF1A methylation has recently been associated with brain metastasis [45]. To directly address tumorigenesis and invasive spread, we adopted a brain-seeding model where spread of human cells could be readily traced. MDA-MB-231 cells stably expressing RASSF1C, injected into the left striatum of SCID mice, formed significantly larger, more-aggressive tumors than either naive or empty vector controls (Figure 7E). The results collectively indicate the role of RASSF1C in invasiveness both in vitro and in vivo. The corroboration of RASSF1C activity with loss of the RASSSF1A transcript also supports increasing importance of *RASSF1A* promoter methylation with invasion, metastasis, and adverse outcome in multiple human tumors.

## Discussion

SRC and its associated family members play multiple roles in normal cell homeostasis controlling: cell proliferation and survival; cytoskeleton organization; cell shape; cell-cell and cell-ECM contacts; and motility. Deregulation of these activities promotes tumorigenesis, cancer cell invasion, and metastasis [46]. Evidence exists for elevation of SRC activity in tumors as a result of growth factor or cytokine signaling, but a clear somatic event is lacking [47]. In addition to mutational events and copy-number variations that drive tumors, epigenetic alterations are known to be a major contributing factor to disease progression and prognosis. We have identified that, similar to dRASSF8 binding to dCSK in *Drosophila* [30], RASSF1A associates with CSK and serves to keep SRC repressed. Inactivation of RASSF1A expression is the most widely observed epigenetic event across all sporadic human malignancies and has been confirmed to be a deleterious prognostic factor in meta-analyses of breast, bladder, lung, colorectal, prostate, esophageal, and ovarian cancers [10]. Therefore, the absence of CSK scaffolding to SRC, identified here, is likely to be a contributing factor in association of *RASSF1A* methylation with disease progression. In colorectal cancer, methylation of *RASSF1A* is not only associated with tumor dissemination [10], but it is found to be dramatically elevated in liver metastasis compared to primary tissue [48]. The fact that RASSF1A cooperates with APC loss to promote intestinal tumors [49] suggests that SRC activation may contribute to colorectal tumorigenesis by promoting the tyrosine phosphorylations of both β-catenin and YAP1 that are required for nuclear localization and transcriptional activation [16, 29, 39]. These data therefore provide an explanation for the prognostic value of *RASSF1A* methylation, while also providing a biomarker rational for treating RASSF1A-negative tumors with SRC inhibitors.

EMT is characterized by loss of cell-cell contacts through inactivation of E-cadherin and gain of mesenchymal markers. We find that RASSF1C expression in epithelial cells replicates a partial-EMT phenotype [18] where E-cadherin is expressed, together with the mesenchymal marker vimentin (data not shown), but prevented from forming stable contacts. Further, we observed that RASSF1C expression allows β-catenin and YAP1, normally sequestered at the membrane, to translocate to the nucleus. In addition to SRC-mediated destruction of cell-cell contacts in epithelial cells, we find that mesenchymal MDA-MB-231 cells also appear to require YES-mediated phosphorylation of YAP for nuclear targeting, as has been implicated previously for RUNX2 complexes [39] and in cancer-associated fibroblasts (CAFs) in response to mechanical stress [50]. Interestingly, the association of RASSF1A with filamin A and Arp3 (Figure 2A) suggests that actin dynamics and the mechanical stress response may by sensed by RASSF1A and contribute to SRC and YAP1 activation. RASSF1C also promotes nuclear accumulation of YAP1/β-catenin and upregulation of target genes that promote tumorigenesis, including BCL2L1 and BIRC5. YAP1/β-catenin promote cancer cell proliferation and tumorigenesis via TBX5 [16] but also promote overgrowth of the heart through binding to TEAD [24]. Our data suggest that, in breast cancer cells, YAP1/β-catenin complex with TBX3, which phenocopies TBX5 at *BCL2L1* and *BIRC5* but additionally promotes *cMYC* expression, potentially by combining known TEAD- and TBX3-binding elements [28, 42].

We also confirm that RASSF1A activation of the hippo pathway maintains phosphorylation of YAP1, specifically pS127-YAP1 (Figures 1B and S1A), preventing association with TEAD [6, 15]. Upon loss of the pS127, YAP1 is permissive for activation but requires additional modifications for nuclear localization and transcriptional transactivation [15, 16, 29, 39], which we find are dependent on loss of the RASSF1A transcript and expression of RASSF1C (Figure 7F). Moreover, YAP-dependent tumorigenesis, mammosphere formation, and growth in soft agar have been attributed to BIRC5 expression [16, 42], which together with MYC-mediated invasion [44] is in line with the formation of larger, more-aggressive mammospheres in cells expressing RASSF1C and larger tumors in vivo. These results also explain the emerging association of both YAP1 and TBX3 with invasive cancers and motility during development [51, 52].

These phenotypes are supported by investigation of the clinical data sets that now include CGI methylation and protein phosphorylation, corroborating links of *RASSF1A* methylation with invasive spread and explaining prognostic association of this epigenetic event. Given the differential regulation of SFK complexes and YAP activity by RASSF1A and RASSF1C isoforms, the balance of expression of these two RASSF1 isoforms may provide an elegant mechanism for fine-tuning SFK signaling and YAP1 transcriptional activity during development and in emerging epithelial cancers. This isoform switch of RASSF1A may govern further SFK-regulatory events by the RASSF family, such as RASSF1C homolog Rapl-mediated regulation of SRC activation in innate immunity [53]. Moreover, is likely to be a common mechanism that adds complexity to the functionality of genetic information encoded by a single gene.

## Experimental Procedures

### Proximity Ligation Assay

Proximity ligation assay was performed as described using the Duolink Starter Kit (Sigma). H1299 cells were transfected and treated as per instructions before overnight incubation with primary antibodies (SRC, SRC pY416, and SRC pY527 [Cell Signaling]; ZsGreen [Clontech]; RASSF1A [Epitomics]; RASSF1C [Abcam]; FLAG tag [Sigma]; and HA tag [Millipore]) and secondary antibodies for 1 hr. Hybridization was performed for 30 min in a humidified chamber, ligation reactions for 30 min, amplification for 100 min, and DAPI was used for cell detection. The dot-like structures were imaged using Zeiss LSM780 microscope using a 63× objective. For each cell, five z stack images were taken and analyzed with BlobFinder V3.2 (Uppsala University) [54]. The average values, SEM, and significance were calculated using Prism 6.0 (Graphpad) software.

### Real-Time Molecular Visualization

MCF7 cells (2 × 10^5^ cells/condition) on glass bottom plates (Ibidi) were transfected with GFP-E-cadherin and DsRed or DsRed-RASSF1C plasmids. Cells were grown in complete media until imaging, when media was changed to DMEM/F-12 media without phenol red and supplemented with 10% (v/v) FBS and 100 U/ml Pen/strep. Cells were imaged using Zeiss LSM780 confocal microscope using a 63× objective, for the time-lapse one image per second for a total of 200 s. Tracking of internalized E-cadherin was achieved using Imaris 7.71 software (Bitplane; ANDOR). Threshold was determined for each video and varied between 0.4 and 0.6 μm in size. The average distance traveled was set at a threshold of 1 μm. A Quality filter was applied and the threshold set at 15. The data were extracted using the Vantage feature and average values, SEM, and significance were calculated using Prism 6.0 (Graphpad) software. On average, five cells and 700 vesicles each were counted per experiment, and the data shown represent three independent experiments.

### Fluorescence Recovery after Photobleaching

MCF7 cells (2 × 10^5^ cells/condition) were plated on 35-mm glass bottom plates (Ibidi). Cells were grown in complete media until imaging, when media was changed to DMEM/F-12 media without phenol red and supplemented with 10% (v/v) FBS and 100 U/ml pen/strep. Cells were imaged using Zeiss LSM780 confocal microscope using a 63× (NA 1.4) objective. Each bleach was done at full laser power, and two pulses were used in order to achieve 60% bleach per region of interest. Images were taken every 2 s for 5 min after the bleach. Data were analyzed using easyFRAP software [55]. The immobile and mobile fractions were calculated using double exponential formula (intensity [I] = IE − I1 × e(−t/T1) − I2 × e(−t/T2)).

### Animal Experiments

Mice were anesthetized and skull burr-hole drilled. Animals were each focally injected with 5 × 10^3^ MDA-MB-231 tumor cells expressing empty vector (pCDNA3) or MYC-RASSF1C in 0.5 μl PBS in the left striatum using a 75-mm-tipped glass microcapillary (Clark Electromedical Instruments). At day 21, all animals were transcardially perfusion fixed under terminal anesthesia (n = 4 per group) and brains were post-fixed, cryoprotected, embedded, and frozen in isopentane at −40°C. To assess areas of tumor colonization, photomicrographs of each brain section were obtained using ScanScope CS slide scanner (Aperio) and analyzed using ImageScope (Aperio). For immunofluorescence, sections were streptavidin and biotin blocked, incubated with anti-CD34 primary antibody (Abcam; brain vessels) or anti-vimentin antibody (VectorLabs; tumor cells), washed, and incubated with a streptavidin-Cy3 fluorophore or AMCA-conjugated secondary antibody (Invitrogen; 1:100) for 30 min. Expanded animal experimental procedures are outlined in Supplemental Experimental Procedures.

All animal experiments were approved by the University of Oxford local animal ethical committee and were performed according to terms of a license granted by the UK Home Office, adhering to the Animals (Scientific Procedures) Act 1986.

### The Cancer Genome Project Analysis

The data were downloaded from cBioPortal for Cancer Genomics [56, 57] and analyzed with SPSS 21.0 and R version 3.0.1 software. For each clinical data set, cases with missing (NA) methylation, protein, or gene expression values were excluded where appropriate. The Shapiro-Wilk test was used to assess distribution of data sets, null hypothesis of normal distribution was rejected at p < 0.05 level, and the non-parametric Spearman-Rho test was used for correlation. The non-parametric Kruskal-Wallis or Jonckheere-Terpstra was used to compare the differences of either protein or gene expression levels between the appropriate groups analyzed. The Molecular Signatures Database (MSigDB v4.0) [58] was used to select gene sets, and POOLA_ INVASIVE_ BREAST_ CANCER_ UP [59], BIDUS_ METASTASIS_ UP [60], ANASTASSIOU_ CANCER_ MESENCHYMAL_ TRANSITION_ SIGNATURE [61], CORDENONSI_ YAP_ CONSERVED_ SIGNATURE [62], REACTOME_ YAP1_ AND_ WWTR1_ TAZ_ STIMULATED_ GENE_ EXPRESSION, and BLALOCK_ ALZHEIMERS_ DISEASE_ INCIPIENT_ UP [63] were selected as either test or control gene signature sets. The non-parametric Mann-Whitney test was used to compare variation in gene expression between the groups analyzed and the Fisher’s exact test used to compare differences in frequency distributions.

### Statistics

For all in vitro experiments, statistical analysis was carried out using a Student’s t test. Tumor areas were compared by ANOVA followed by post hoc Newman-Keuls t tests. All data are expressed as mean ± SEM.

## Author Contributions

N.V. and S.S. helped design and performed the majority of experiments. M.S.S. and N.S. contributed the in vivo experiments. A.M.G. performed the bioinformatics analyses with advice from F.B. L.B., A.P., and K.S.Y. contributed to nuclear localization and hippo pathway experiments. D.P. and S.S. performed the spheroid analysis. C.R.G. assisted with TBX reagents and advice. P.T. advised S.S. on imaging analysis of Src and E-cadherin. E.O. is responsible for the concept and designed and wrote the manuscript with S.S. and N.V.

## Supplemental Information

Document S1. Figures S1–S7, Tables S1 and S2, and Supplemental Experimental Procedures

Movie S1. Time-Lapse of the Movement of Internalized E-Cadherin Left: control cell. Right: RASSF1C-expressing cell. Images were taken every second for 200 seconds. Video represents 100 seconds of time lapse.

Movie S2. Time-Lapse of the Movement of Internalized Control Cells Expressing Mutant E-Cadherin Images were taken every second for 200 seconds. Video represents 100 seconds of time lapse.

Movie S3. Time-Lapse of the Movement of Internalized RASSF1C Cells Expressing Mutant E-Cadherin Images were taken every second for 200 seconds. Video represents 100 seconds of time lapse.

Movie S4. Representative Video for the FRAP Analysis of Control Cells Red rectangle: site of photobleaching. Video represents 3 min of time lapse made of 100 consecutive images taken.

Movie S5. Representative Video for the FRAP Analysis of RASSF1C Cells Red rectangle: site of photobleaching. Video represents 3 min of time lapse made of 100 consecutive images taken.

Document S2. Article plus Supplemental Information
