# Supplementary material for: Alternate RASSF1 Transcripts Control SRC Activity, E-Cadherin Contacts, and YAP-Mediated Invasion
Source: Curr Biol. 2015 Dec 7;25(23):3019–34. doi: 10.1016/j.cub.2015.09.072 (PMC4683097; doi:10.1016/j.cub.2015.09.072)
Supplement: Document S1. Figures S1–S7, Tables S1 and S2, and Supplemental Experimental Procedures [file mmc1.pdf]

Current Biology

Supplemental Information

Alternate RASSF1 Transcripts Control SRC Activity, E-Cadherin Contacts, and YAP-Mediated Invasion

Nikola Vlahov, Simon Scrace, Manuel Sarmiento Soto, Anna M. Grawenda, Leanne Bradley, Daniela Pankova, Angelos Papaspyropoulos, Karen S. Yee, Francesca Buffa, Colin R. Goding, Paul Timpson, Nicola Sibson, and Eric O'Neill

Figure S1

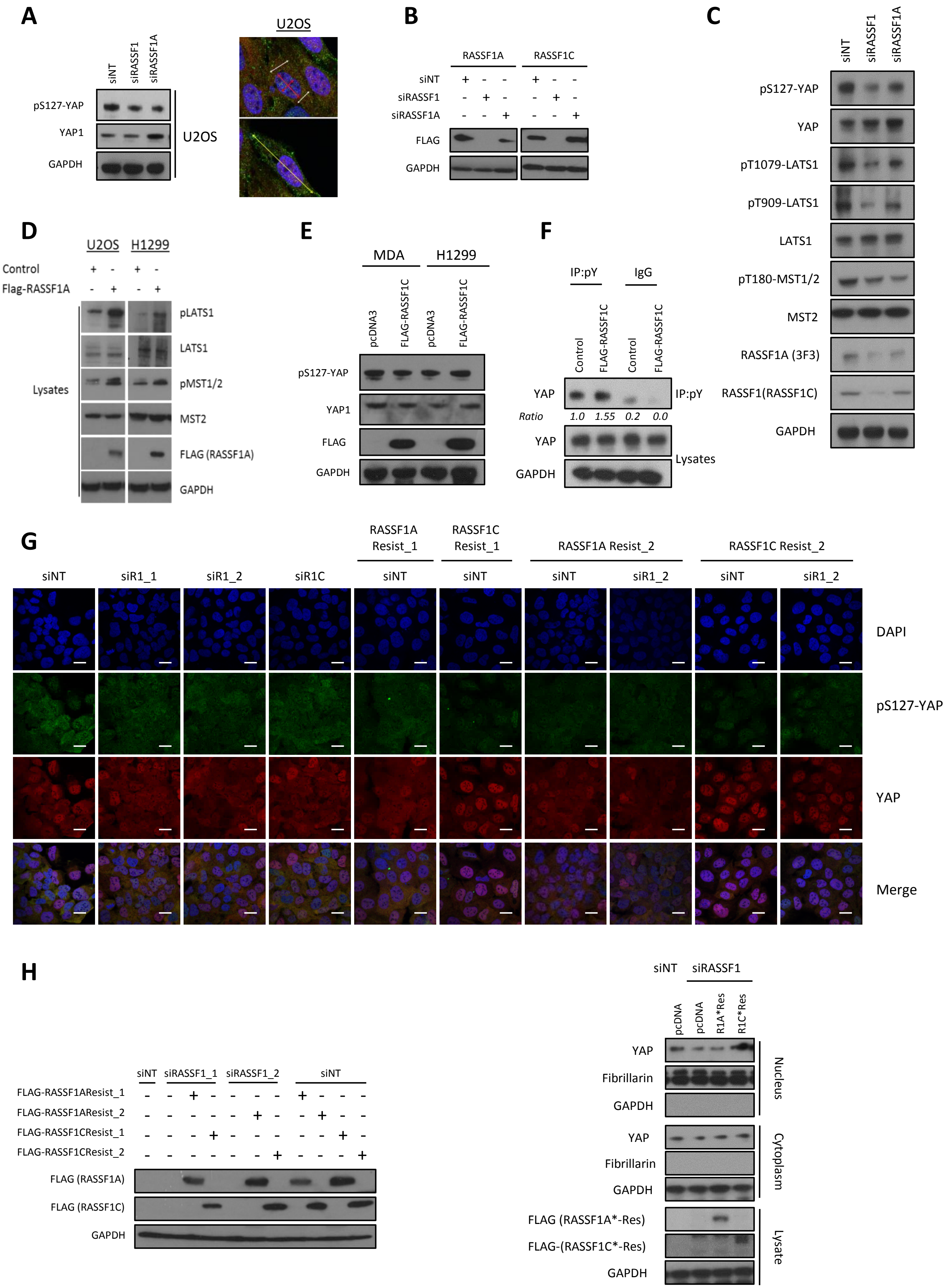
A
U2OS
siNT
siRASSF1
siRASSF1A
pS127-YAP
YAP1
GAPDH
U2OS
B
RASSF1A
RASSF1C
siNT
siRASSF1
siRASSF1A
FLAG
GAPDH
C
pS127-YAP
YAP
pT1079-LATS1
pT909-LATS1
LATS1
pT180-MST1/2
MST2
RASSF1A (3F3)
RASSF1(RASSF1C)
GAPDH
D
U2OS
H1299
Control
Flag-RASSF1A
Lysates
pLATS1
LATS1
pMST1/2
MST2
FLAG (RASSF1A)
GAPDH
E
MDA
H1299
pcDNA3
FLAG-RASSF1C
pS127-YAP
YAP1
FLAG
GAPDH
F
IP:pY
IgG
Control
FLAG-RASSF1C
YAP
Ratio
1.0
1.55
0.2
0.0
IP:pY
Lysates
GAPDH
G
RASSF1A Resist_1
RASSF1C Resist_1
RASSF1A Resist_2
RASSF1C Resist_2
siNT
siR1_1
siR1_2
siR1C
DAPI
pS127-YAP
YAP
Merge
H
siNT
siRASSF1_1
siRASSF1_2
FLAG-RASSF1AResist_1
FLAG-RASSF1AResist_2
FLAG-RASSF1CResist_1
FLAG-RASSF1CResist_2
FLAG (RASSF1A)
FLAG (RASSF1C)
GAPDH
siRASSF1
pcDNA
R1A*Res
R1C*Res
Fibrillarin
Nucleus
Cytoplasm
FLAG (RASSF1A*-Res)
FLAG-(RASSF1C*-Res)
Lysate

Figure S1 (Related to Figure 1). RASSF1C Leads to Nuclear Localisation of YAP1.

(*A*) Left, lysates from siNT, siRASSF1 or siRASSF1A treated U2OS cells western blotted for total YAP1 or pS127-YAP1. Images indicate vectors taken for determination of cytoplasmic (value = average of two vectors/cell, white arrows) nuclear (value = average of two vectors/cell, red arrows) staining and cell equatorial vector (yellow arrows). (*B*) Western blot of knockdown of exogenously expressed FLAG-RASSF1A (left) or FLAG-RASSF1C (right) by siRASSF1 or siRASSF1A. (*C*) Immunoblotting of lysates from U2OS cells transfected with siNT, siRASSF1 or siRASSF1A. (*D*) Immunoblotting of lysates from U2OS and H1299 cells transfected with either Control or Flag-RASSF1A. (*E*) Levels of total YAP1 and pS127-YAP1 in MDA-MB-231 (Left) and H1299 (Right) cells (RASSF1A ^{methyl}) in the presence and absence of FLAG-RASSF1C (*F*) Immunoprecipitation with total pY antibody from U2OS cells transfected with either Control or Flag-RASSF1C (Right). (*G*) Immunofluorescence images of YAP1 and pS127-YAP1 nuclear/cytoplasmic localisation of U2OS cells, transfected with siRNA resistant forms of RASSF1A and RASSF1C and treated with siNT, siRASSF1 (R1_1 or R1_2) or siRASSF1C (siR1C). Images represent siNT for R1_1 shown in Figure 1D. (H) Western blot showing RASSF1A and RASSF1C resistant construct expression is unaffected by siRNA treatment with siRASSF1_1 or siRASSF1_2 (Left). Nuclear/Cytoplasmic fractionation for YAP1 of U2OS cells transfected with either siNT or siRASSF1 and siRNA resistant versions of RASSF1A and RASSF1C (Right). All scale bars represent 20 μm.

Figure S2

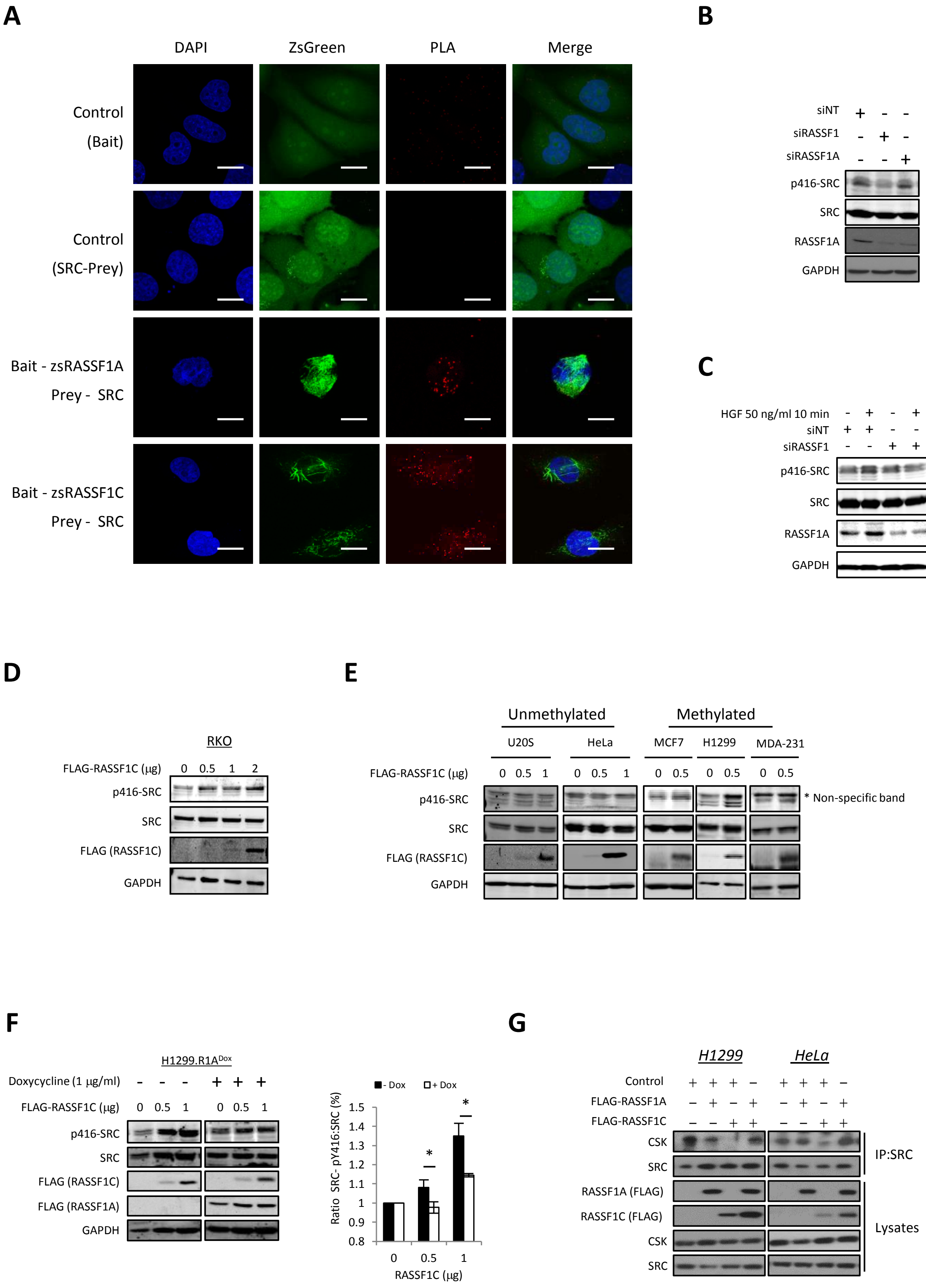
A
DAPI
ZsGreen
PLA
Merge
Control
(Bait)
Control
(SRC-Prey)
Bait - zsRASSF1A
Prey - SRC
Bait - zsRASSF1C
Prey - SRC
B
siNT + - -
siRASSF1 - + -
siRASSF1A - - +
p416-SRC
SRC
RASSF1A
GAPDH
C
HGF 50 ng/ml 10 min - + - +
siNT + + - -
siRASSF1 - - + +
p416-SRC
SRC
RASSF1A
GAPDH
D
RKO
FLAG-RASSF1C (μg) 0 0.5 1 2
p416-SRC
SRC
FLAG (RASSF1C)
GAPDH
E
Unmethylated
Methylated
U20S
HeLa
MCF7
H1299
MDA-231
FLAG-RASSF1C (μg) 0 0.5 1 0 0.5 1 0 0.5 0 0.5 0 0.5
p416-SRC
SRC
FLAG (RASSF1C)
GAPDH
* Non-specific band
F
H1299.R1A^Dox
Doxycycline (1 μg/ml) - - - + + +
FLAG-RASSF1C (μg) 0 0.5 1 0 0.5 1
p416-SRC
SRC
FLAG (RASSF1C)
FLAG (RASSF1A)
GAPDH
- Dox
+ Dox
Ratio SRC- pY416:SRC (%)
1.5
1.4
1.3
1.2
1.1
1
0.9
0.8
*
*
0
0.5
1
RASSF1C (μg)
G
H1299
HeLa
Control + + + – + + + –
FLAG-RASSF1A – + – + – + – +
FLAG-RASSF1C – – + + – – + +
CSK
SRC
IP:SRC
RASSF1A (FLAG)
RASSF1C (FLAG)
CSK
SRC
Lysates

Figure S2 (Related to Figure 2) RASSF1C Expression Activates SRC Only When RASS1F1A is Absent. (*A*) Representative immunofluorescence images showing the different levels of colocalisation of RASSF1A and RASSF1C (Baits) with SRC (Preys) using proximity ligation assay (Duolink). (*B*) HeLa cells transfected with non-targeting siRNA (NT), siRNA targeting all RASSF1 isoforms (siRASSF1) or specifically RASSF1A (siRASSF1A). Blotted for SRC auto-phosphorylation site, Y416, using antibodies against avian SRC (p416-SRC). (*C*) Western blot of pY416-SRC in HeLa cells transfected with siNT or siRASSF1 prior to stimulation with 50 ng/ml HGF. (*D*) Western blot p416-SRC in RKO cells, methylated for RASSF1A, transfected with increasing concentrations of FLAG-RASSF1C. (*E*) Western blot of p416-SRC in RASSF1A positive (U2OS and HeLa) and RASSF1A negative (MCF7 and MDA-MB-231) cells transiently transfected with FLAG-RASSF1C. (*F*) H1299.R1A^{Dox} TET-ON FLAG-RASSF1A inducible cells transiently transfected with indicated concentrations of FLAG-RASSF1C and treated with doxycycline as indicated. Graph shows quantitation of SRC-pY416/SRC ratio. Error bars depict 1 x SEM. (*G*) Immunoprecipitation of SRC from lysates from H1299 and HeLa cells transfected with either Flag-RASSF1A or Flag-RASSF1C. All scale bars represent 20 μm.

Figure S3

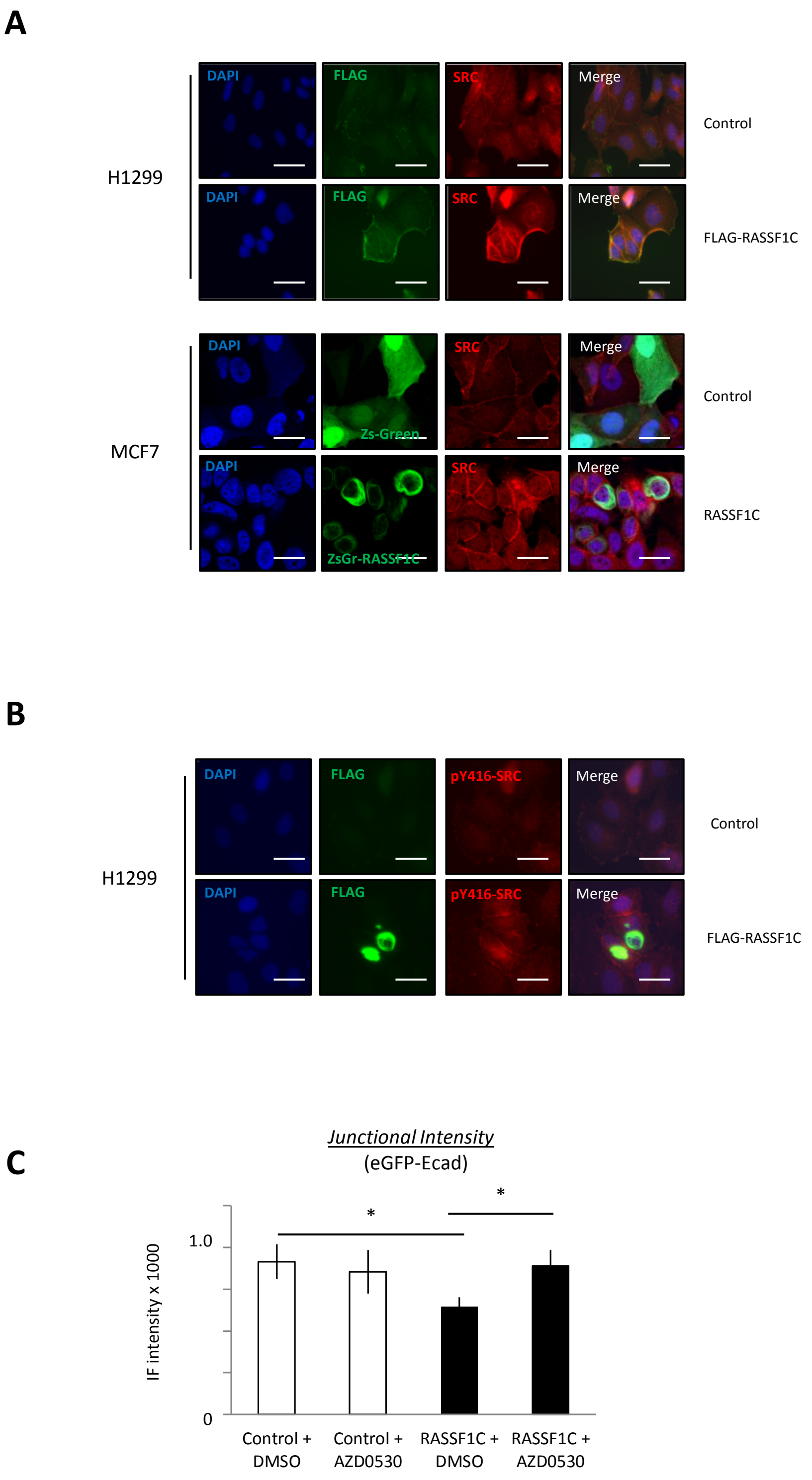
A
H1299
DAPI
FLAG
SRC
Merge
Control
FLAG-RASSF1C
MCF7
Zs-Green
ZsGr-RASSF1C
RASSF1C
B
H1299
pY416-SRC
C
Junctional Intensity
(eGFP-Ecad)
IF intensity x 1000
1.0
0
*
Control + DMSO
Control + AZD0530
RASSF1C + DMSO
RASSF1C + AZD0530

Figure S3 (Related to Figure 3). RASSF1C Localises SRC activation to Adherens Junction.

(*A*) Immunofluorescence images of SFK localisation in H1299 cells transfected with empty vector or FLAG-RASSF1C (top) and MCF7 cells transfected with Zs-Green empty vector or Zs-Green-RASSF1C (bottom). (*B*) Immunofluorescence images of pY416-SRC in H1299 cells transfected with empty vector or greater expression levels of FLAG-RASSF1C after 24hrs. (*C*) Junctional intensity of MCF7 cells transfected with eGFP-E-cadherin and either DsRed or DsRed-RASSF1C cells, treated with DMSO or AZD0530 (2.5 μM, 18 h). The intensity was measured using the Zeiss Zen 2011 software. All scale bars represent 20 μm.

Figure S4

A

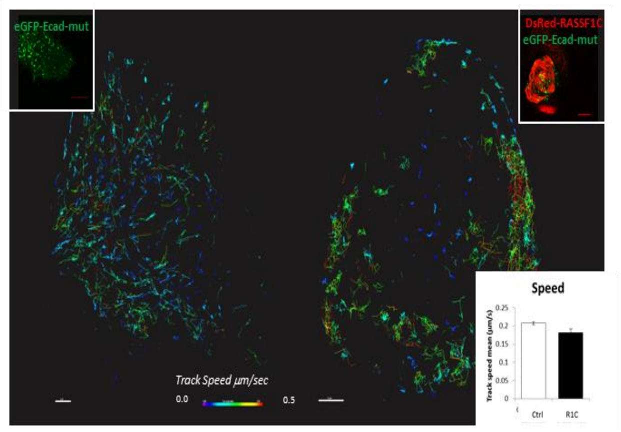
eGFP-Ecad-mut
DsRed-RASSF1C
eGFP-Ecad-mut
Speed
Track speed mean (µm/s)
Ctrl
R1C
Track Speed µm/sec
0.0
0.5
5 µm
Track length
Direction
Track Length
Length (µm)

B

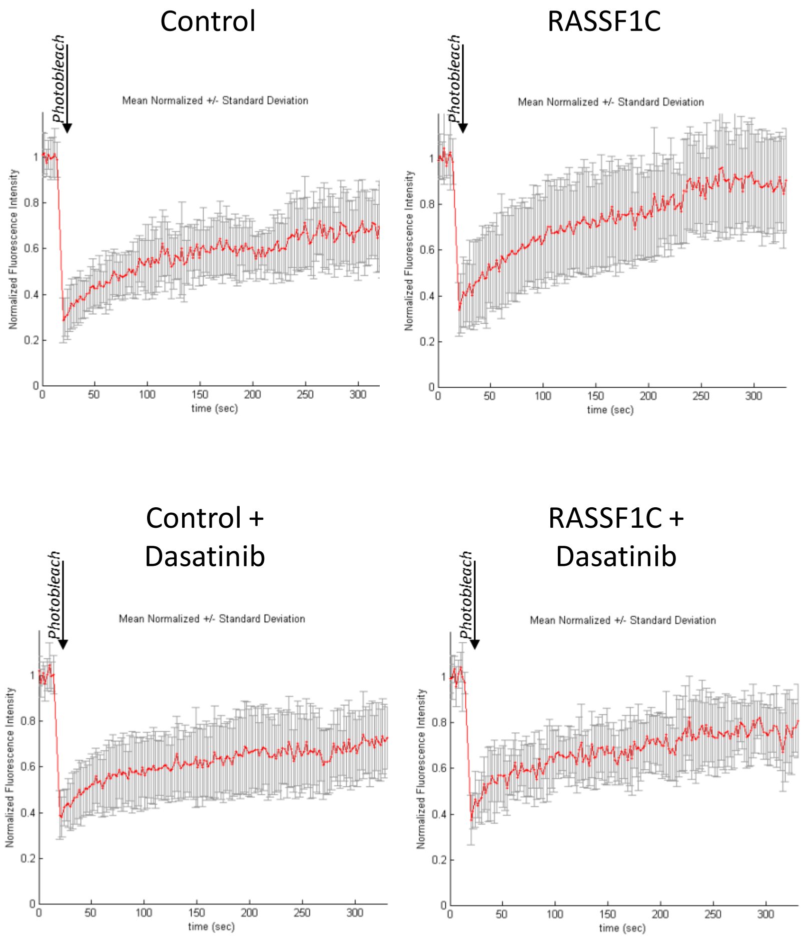
Control
RASSF1C
Control + Dasatinib
RASSF1C + Dasatinib
Photobleach
Mean Normalized +/- Standard Deviation
Normalized Fluorescence Intensity
time (sec)

Figure S4 (Related to Figure 4). Representative Graphs for the FRAP Analysis.

(*A*) Representative images of the tracking of all the vesicles in DsRed Control and DsRed-RASSF1C expressing MCF7 cells and transfected with eGFP-E-cadherin mutant (Y753F, Y754F, Y755F) (Imaris). Bar graphs show the analysis of the mean speed heatmap (Bottom left) or distance (Bottom right) of the vesicles in control and RASSF1C cells. For each analysis 5 cells per experiment were used and an average of 700 vesicles were tracked (bars). The results are from three independent experiments including Movie S2 and S3. Immunofluorescence images at the top demonstrate the accumulation of E-cadherin mutant and the expression of DsRed-RASSF1C. (*B*) Example graphs from FRAP analysis of junctional GFP-E-cadherin in MCF7 cells transfected with Ds-Red empty vector or Ds-Red-RASSF1C, treated with Dasatinib. Representative FRAP timelapse in Movies S4 and S5.

Figure S5

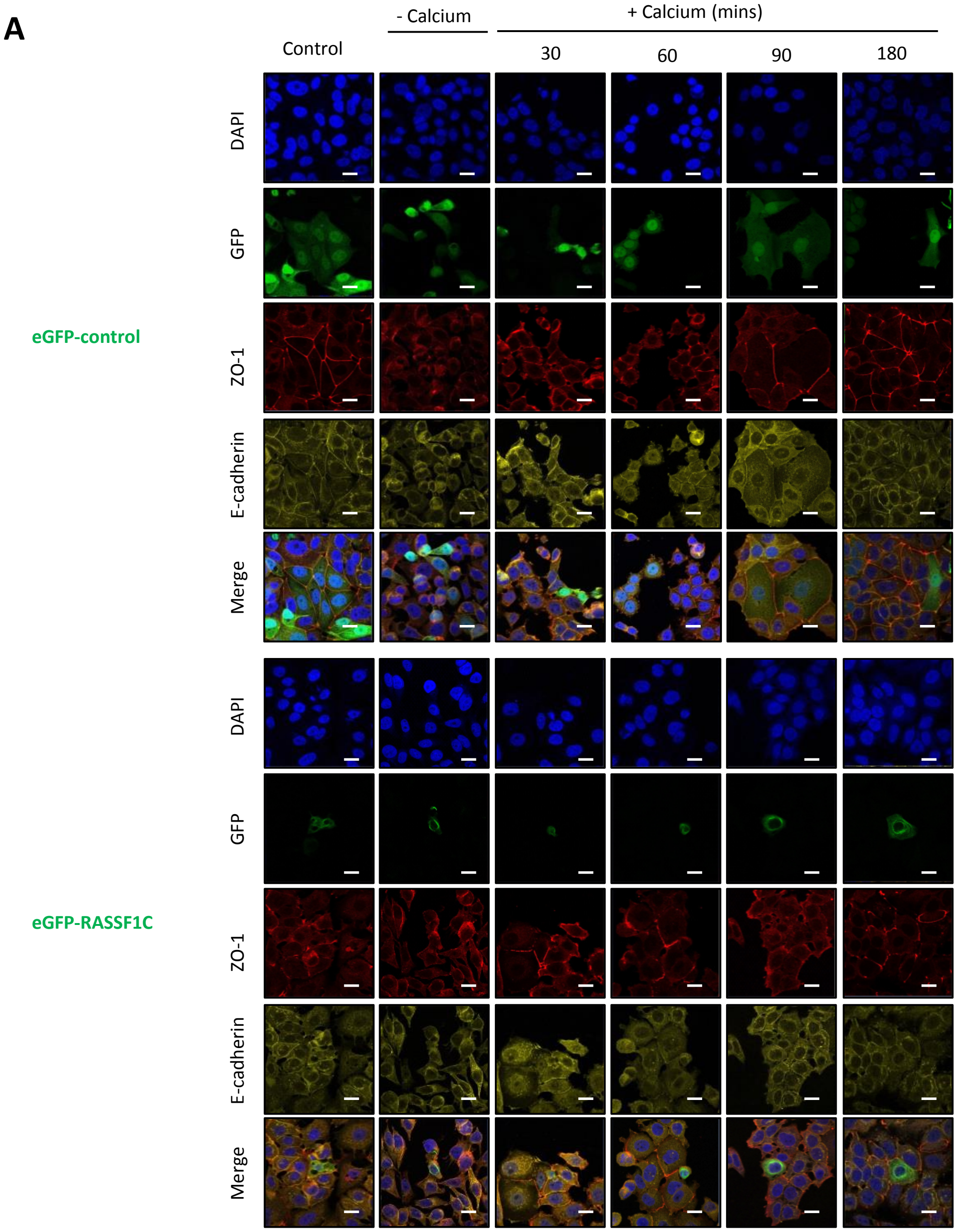
A
- Calcium
+ Calcium (mins)
Control
30
60
90
180
DAPI
GFP
ZO-1
E-cadherin
Merge
eGFP-control
DAPI
GFP
ZO-1
E-cadherin
Merge
eGFP-RASSF1C

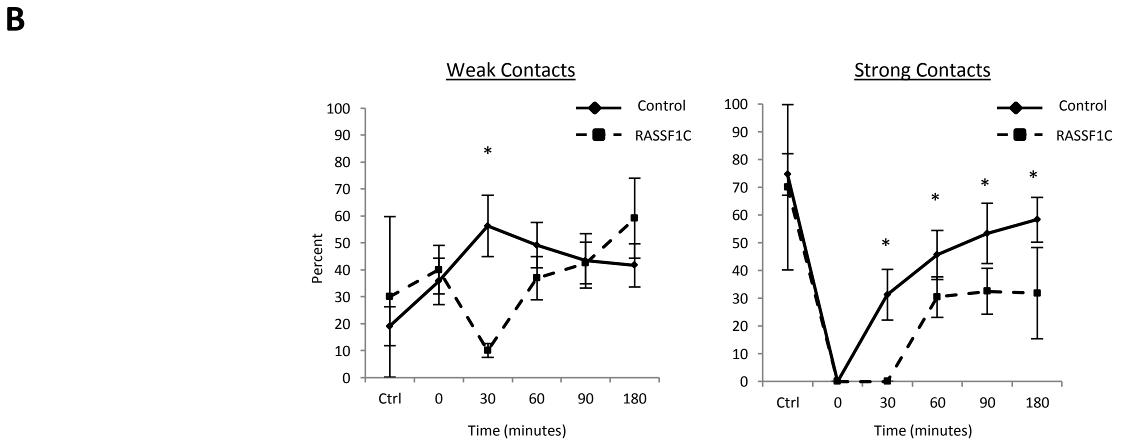
B
Weak Contacts
Control
RASSF1C
Percent
Ctrl
0
30
60
90
180
Time (minutes)
Strong Contacts
Control
RASSF1C
Ctrl
0
30
60
90
180
Time (minutes)

Figure S5 (Related to Figure 4). RASSF1C Affects the Establishment of Cell-Cell Junctions.

(*A*) Example images of Calcium Switch Assay in MCF7 cells transfected with GFP-empty vector or GFP-RASSF1C. Control represents cells that have remained in media containing calcium. All scale bars represent 20 μm. (*B*) Quantitation of number of weak contacts (left) and strong contacts (right) (defined in methods) formed at indicated time points.

Figure S6

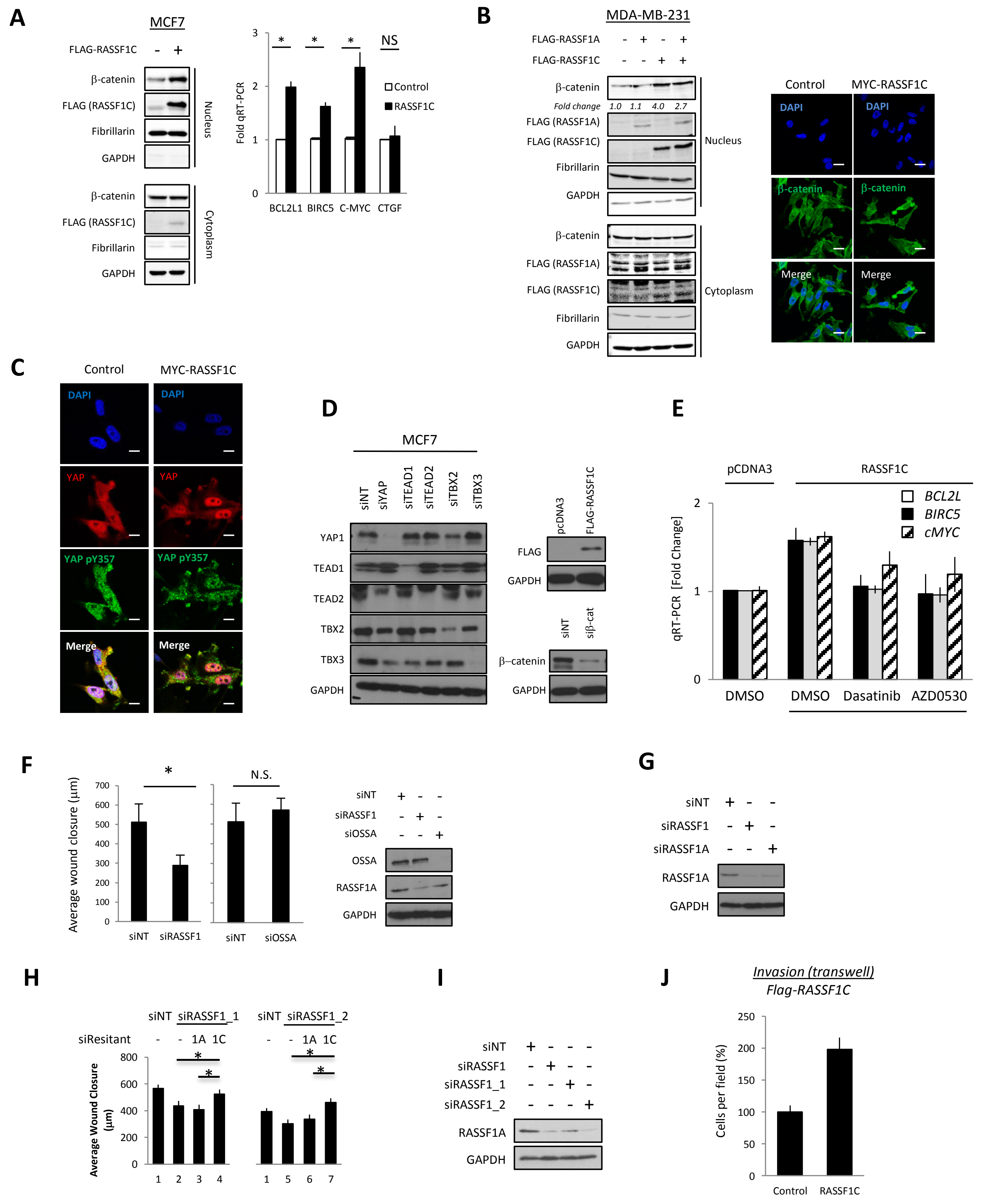
A
MCF7
FLAG-RASSF1C - +
β-catenin
FLAG (RASSF1C)
Fibrillarin
GAPDH
Nucleus
Cytoplasm
Fold qRT-PCR
* * * NS
Control
RASSF1C
BCL2L1 BIRC5 C-MYC CTGF
B
MDA-MB-231
FLAG-RASSF1A - + - +
FLAG-RASSF1C - - + +
Fold change 1.0 1.1 4.0 2.7
FLAG (RASSF1A)
FLAG (RASSF1C)
Control
MYC-RASSF1C
DAPI
β-catenin
Merge
C
YAP
YAP pY357
D
MCF7
siNT siYAP siTEAD1 siTEAD2 siTBX2 siTBX3
YAP1
TEAD1
TEAD2
TBX2
TBX3
pcDNA3 FLAG-RASSF1C
FLAG
siNT siβ-cat
E
pCDNA3
RASSF1C
BCL2L
BIRC5
cMYC
qRT-PCR [Fold Change]
DMSO DMSO Dasatinib AZD0530
F
Average wound closure (µm)
* N.S.
siNT siRASSF1 siNT siOSSA
siNT + - -
siRASSF1 - + -
siOSSA - - +
OSSA
RASSF1A
G
siNT + - -
siRASSF1 - + -
siRASSF1A - - +
H
siNT siRASSF1_1 siNT siRASSF1_2
siResitant - - 1A 1C - - 1A 1C
Average Wound Closure (µm)
1 2 3 4 1 5 6 7
I
siNT + - - -
siRASSF1 - + - -
siRASSF1_1 - - + -
siRASSF1_2 - - - +
J
Invasion (transwell)
Flag-RASSF1C
Cells per field (%)
Control RASSF1C

Figure S6 (Related to Figures 5 & 6). RASSF1C promotes β-catenin/YAP1 Dependent Transcription and Invasion.

(*A*) Nuclear/Cytoplasmic fractionation of MCF7 cells expressing empty vector or FLAG-RASSF1C (left). Quantification of qRT-PCR for YAP-β-catenin-TBX target genes BCL2L1 and BIRC5, β-catenin-TCF/LEF target gene c-MYC and YAP/TEAD target gene CTGF in MCF7 cells transfected with empty vector or FLAG-RASSF1C (Right). * represents $p < 0.05$. (*B*) Left: Nuclear/Cytoplasmic fractionation of MDA-MB-231 cells transiently transfected with empty vector, FLAG-RASSF1A, FLAG-RASSF1C or Both FLAG-RASSF1A and FLAG-RASSF1C to show β-catenin localisation, quantitated by Licor imaging software. Right: Immunofluorescence depicting β-catenin localisation in MDA-MB-231 cells transiently transfected with empty vector or MYC-RASSF1C. (*C*) Representative images for the localisation of YAP1 and pY357-YAP1 in MDA-MB-231 cells transfected with Control or MYC-RASSF1C vectors. All scale bars represent 20 μm. (D) Lysates from MCF7 cells with the indicated siRNAs used in qRT-PCR assays in Fig. 6B. (*E*) Quantification of qRT-PCR for BCL2L1, BIRC5 and c-MYC genes in MCF7 cells transfected with empty vector or FLAG-RASSF1C and treated with either Dasatinib (50 nM, 18 h) or AZD0530 (2.5 μM, 18 h). (*F*) Left: Quantification of scratch wound assay of siNT vs siRASSF1 (p= 2.6 x 10-4) and siNT vs siRNA targeting the oxidative stress-associated activator of SRC, OSSA (p= 0.16). Right: Western blot showing knock down of RASSF1A by siRASSF1 and OSSA by siOSSA. (*G*) Western blot of knockdown of RASSF1A by siRASSF1 and siRASSF1A, related to Fig. 3D. (*H*) Quantitation of scratch wound assay of HeLa cells transfected with siNT or two different siRNAs to RASSF1 (siRASSF1_1 or siRASSF1_2) and siRNA resistant constructs of either RASSF1A or RASSF1C as described in Supplementary Fig S1g, S1H (siRASSF1_1: siNT vs siRASSF1_1 p= 5.7 x 10-9, RASSF1C vs pcDNA p= 6.2 x 10-5, RASSF1C vs RASSF1A p= 9.7 x 10-10. siRASSF1_2: siNT vs siRASSF1_2 p= 1.9 x 10-6, RASSF1C vs pcDNA p= 5.4 x 10-12, RASSF1C vs RASSF1A p= 1.2 x 10-8). (*I*) Western blot showing knockdown of endogenous RASSF1A by siRASSF1 (SMARTpool), siRASSF1_1 and siRASSF1_2. (*J*) Quantification of transwell assay with MDA-MB-231 cells stably expressing empty vector or MYC-RASSF1C. Error bars depict 1 x SEM.

Figure S7

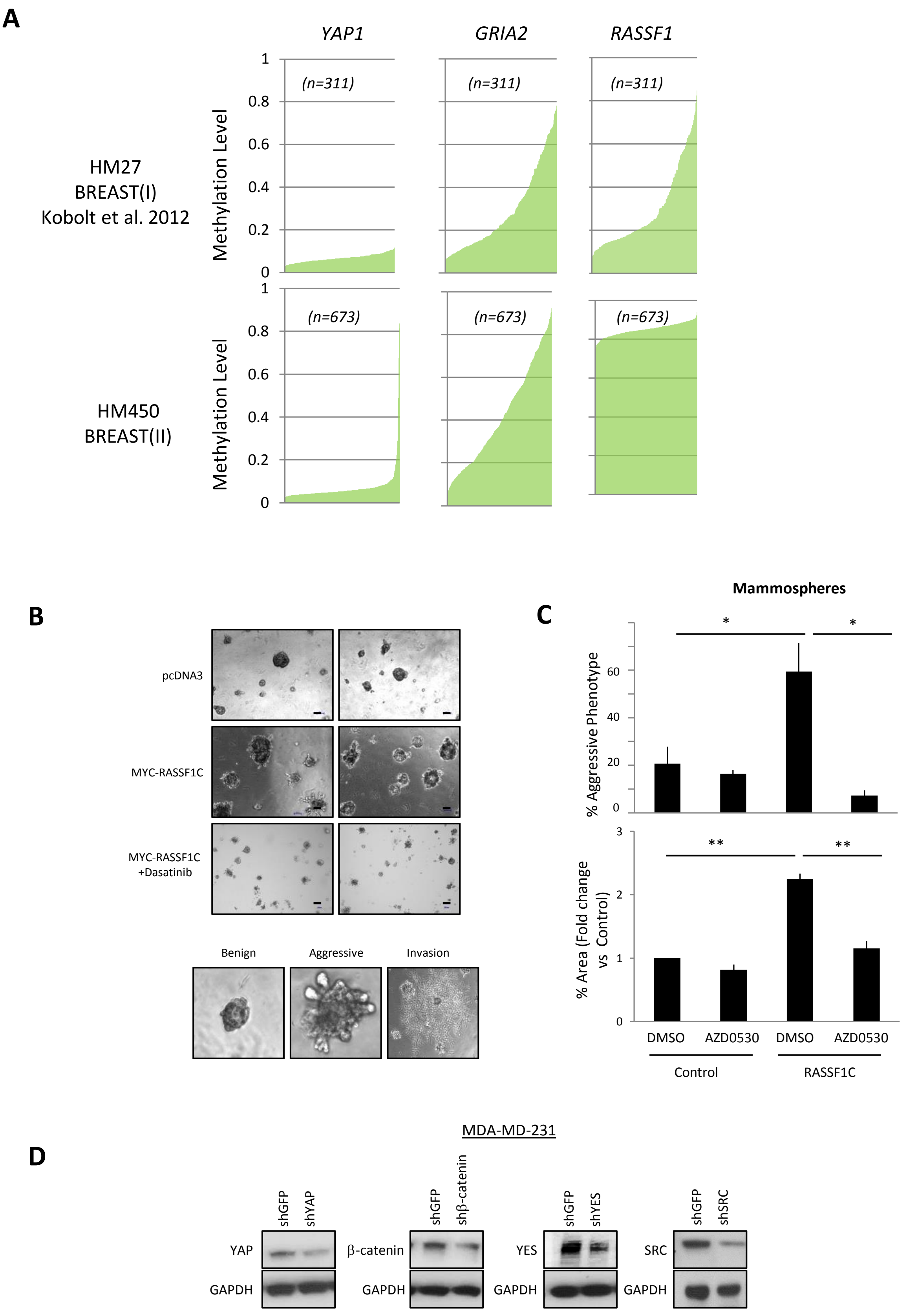

A
YAP1
GRIA2
RASSF1
(n=311)
(n=673)
Methylation Level
1
0.8
0.6
0.4
0.2
0
HM27
BREAST(I)
Kobolt et al. 2012
HM450
BREAST(II)
B
pcDNA3
MYC-RASSF1C
MYC-RASSF1C
+Dasatinib
Benign
Aggressive
Invasion
C
Mammospheres
% Aggressive Phenotype
60
40
20
0
*
*
% Area (Fold change vs Control)
3
2
1
0
**
**
DMSO
AZD0530
DMSO
AZD0530
Control
RASSF1C
D
MDA-MD-231
shGFP
shYAP
shβ-catenin
shYES
shSRC
YAP
β-catenin
YES
SRC
GAPDH

Figure S7 (Related to Figure 7) RASSF1C Promotes Invasiveness *in vitro* and *in vivo*.

(*A*) Top: Invasive breast cancer dataset (BREAST (I)) methylation levels (n=311/825) for non-methylated (YAP1) and methylated genes (GRIA2 and RASSF1) using Illumina HM27 beadchi. Bottom: Invasive Breast cancer dataset (Breast (II)) analysed for the same targets using Illumina HM450 beadchip (n=673/1003), identifies similar levels of methylation as HM27 for *YAP1* and *GRIA2* but increased methylation of *RASSF1*. Data sets are non-overlapping and the same samples do not have information from both reads. (*B*) Example images of Mammospheres from MDA-MB-231 cells stably expressing empty vector, MYC-RASSF1C or MYC-RASSF1C treated with dasatinib grown in matrigel. Aggressive phenotype was defined as mammospheres with greater than 5 invasive projections into the surrounding matrigel. Comparative images of benign, aggressive and invasion (invasion of MDA-MB-231 cells stably expressing RASSF1C through matrigel to the base of the tissue culture dish) are also indicated. All scale bars represent 100 μm. (*C*) Mammospheres grown in matrigel from MDA-MB-231 cells stably expressing empty vector or MYC-RASSF1C were treated on days 3, 6 and 9 with either DMSO or SFK inhibitor (1.25 μM AZD0530). Images were taken on day 10. Graphs indicate the number of mammospheres with aggressive phenotypes (Top) and the size of mammospheres (Bottom). (*D*) Immunoblots showing the knockdown of the targeted genes by shRNA in MDA-MB-231 cells used for mammospheres from Figure 7D.

Table S1

Gene expression signature<br>*Significance threshold Mann-Whitney (Bonferroni corrected)	Gene (n)	% Signature Genes with variation in mRNA levels* (expected direction)									% Signature genes increased (^) and decreased (v) in <pS127 or <YAP		
		Glioma			Bladder			Breast (I)			Breast (II)		
		< pS127	< YAP1	Fisher's exact (pS127 vs YAP1)	< pS127	< YAP1	Fisher's exact (pS127 vs YAP1)	< pS127	< YAP1	Fisher's exact (pS127 vs YAP1)	< pS127 (Fisher's exact) ^ vs v	< YAP1 (Fisher's exact) ^ vs v	Fisher's exact (pS127^{low} vs YAP1^{low})
Invasive breast cancer (POOLA) 0.0001	275	32%*	25%*	*0.07*	0.4%	6%	***p*=0.0001**	2%	0.4%	*p*=0.1	^ 15%<br>v 9%<br>*p=0.036*	2.5%<br>4%<br>*p=0.35*	***p*=1.09e^{-07}**
Metastasis (BIDUS) 0.0004	214	17%	4%	*p=0.2*	0	0%	*p*=1	11%	2%	***p*=0.006**	^ 25%<br>v 1%<br>*p=2.77e^{-15}*	3%<br>1%<br>*p*=0.2	***p*=4.52e^{-11}**
Cancer EMT (ANASTASSIOU) 0.0007	64	17%	9%	*p*=0.3	2%	53%	***p*=6.52e^{-12}**	5%	11%	*p*=0.3	^ 89%<br>v 0%<br>*p=2.2e-^{16}*	42%<br>0%<br>*p*=4.5e-^{10}	***p*=2.62e^{-08}**
YAP SIGNATURE (CORDENONSI) 0.0008	57	25%	26%	*p=1*	2%	14%	*p=0.03*	10.5%	25%	*0.08*	^ 38%<br>v 5%<br>*p=2.24e^{-05}*	28%<br>2%<br>*p*=9.3e^{-05}	*p=0.3*
REACTOME_YAP_TAZ 0.002	24	17%	17%	*p*=1.0	4%	8%	*p*=1.0	25%	20%	*p=1*	^ 33%<br>v 0%<br>*p=3.90e-^{03}*	25%<br>0%<br>*p*=0.04792	*p=0.7*
Alzheimer's 0.0001	388	-	-	-	-	-	-	-	-	-	^ 2%<br>v 7%<br>*p=0.0011*	0.5%<br>7%<br>*p*=1.44e^{-07}	***p=0.6***

* opposite direction

Table S1 (Related to Figure 7A). Enrichment analysis of mSigDB invasive signatures in Cohorts of Glioma, Bladder and Breast (I – Koboldt et al. 2012; II - Invasive Carcinoma HM450) from The Cancer Genome Atlas (TCGA). Cohorts were separated into groups of high and low based on the top and bottom 100 zScores for phospho-S127-YAP1 or total YAP1. The number of genes that increased above the significance cut off for each signature (*) in the low group were scored as a percentage. Comparison of changes in pS127-YAP1 to YAP1 indicates specificity for loss of the phospho signal rather than effects of total protein – confirmed by indistinguishable scores for YAP-TAZ signatures. For Breast (II) we additionally show percentage of signatures genes that go in the opposite direction, genes decreased in low groups (v).

Table S2

Gene Signature		pS127-YAP1		YAP1	
	Breast (II) vs.	Fisher's exact	OR	Fisher's exact	OR
POOLA	Breast (I)	$2.85e^{-08}$	8.05	0.07	7.14
	Glioma	**$5.32e^{-06}$**	0.38	**$2.43e^{-15}$**	0.08
	Bladder	$1.98e^{-12}$	49.13	0.06	0.40
BIDUS	Breast (I)	$8.15e^{-08}$	5.07	0.17	3.57
	Glioma	$5.65e^{-07}$	4.35	1	0.87
	Bladder	$< 2.2e^{-16}$	Inf	0.01	Inf
ANASTASSIOU	Breast (I)	$< 2.2e^{-16}$	151.34	$5.04e^{-05}$	6.24
	Glioma	$< 2.2e^{-16}$	37.42	0.14	1.83
	Bladder	$< 2.2e^{-16}$	456.63	1	0.97
YAP/TAZ (REACTOME)	Breast (I)	0.75	1.49	0.74	1.55
	Glioma	0.32	2.45	0.49	2.03
	Bladder	0.02	10.98	0.14	4.39
YAP (CORDENONSI)	Breast (I)	0.0009	5.26	0.83	1.20
	Glioma	0.16	1.92	1	1.09
	Bladder	$5.61e^{-07}$	34.32	0.10	4.39

Table S2 (Related to Figure 7A). Low levels of pS127-YAP1 correlate with invasive signature only in the context of *RASSF1-1α* methylation.

Comparison of invasive gene signatures enrichment (mSigDB database) in Breast II - Invasive Carcinoma HM450 to BREAST (I), Glioma and Bladder of The Cancer Genome Atlas (TCGA). Elevated YAP-TAZ signature genes are equivalent for pS127-YAP1 and YAP1 in all cohorts (Supplementary Table 1) and no significant difference is observed between Breast I and II, however, invasive, metastatic and EMT signatures show highly significant differences between the *RASSF1-1α* methylated Breast II and limited methylated Breast I. Similar significance is observed with Glioma and Bladder where identical HM450 analysis was applied to characterise *RASSF1-1α*.

Supplemental Experimental Procedures

Cell Culture and Transfection.

Cells were purchased from ATCC and maintained in DMEM supplemented with 10 % FBS, 2 mM Glutamine and 100 U/ml Penicillin/Streptomycin (all from Life Technologies) at 37 ^{o}C, 5 % CO_2 in a humidified incubator. Cells were passaged by trypsinising (0.25 % Trypsin EDTA solution (Life Technologies) and reseeding when necessary. Cells were transfected using Lipofectamine 2000 transfection reagent (Life Technologies) according to manufacturer's guidelines. See siRNA sequences table at the end of Experimental Procedures. For experiments investigating SRC activation by RASSF1C, or using growth factors (HGF (Peprotech)), cells were incubated overnight in DMEM containing 0.1 % FBS to remove growth factor stimulation prior to growth factor treatment and cell lysis.

Cell Lysis.

Cells were lysed for western blot using Laemmli lysis buffer (2.5 mM Tris-HCl pH6.8, 2 % SDS, supplemented with protease inhibitor) before boiling (100 ^{o}C, 10 min). Protein concentration of lysates for western blot and prior to addition of lysate to immunoprecipitations was done using Bradford reagent. Absorbance was analysed using POLARstar OMEGA machine at 595 nm.

Mammosphere Growth Assay.

24 well plates were coated with a thick layer (300 μl per well) of matrigel mixed 1:1 with DMEM media without supplements. Matrigel was allowed to set for at least 30 min at 37 ^{o}C before 2500 MDA-MB-231 cells were plated on top of the matrigel. Mammospheres were allowed to form over 10 days changing the media every 3 days. Images of the mammospheres were taken at 4 X magnification with Nikon TE2000 Eclipse microscope using NIS elements software. Image analysis was done using Image J software.

Nuclear/Cytoplasmic Fractionation.

Cells were trypsinised, collected, washed two times with PBS and incubated for 15 min on ice in cytoplasmic lysis buffer (10 mM HEPES pH 7.9, 10 mM KCl, 0.1 mM EDTA, 0.1 mM EGTA, 1 mM DTT, 0.5 mM PMSF) before NP40 detergent was added (final concentration = 0.65 % (v/v)). Cells were vortexed immediately for 10 seconds and centrifuged (1500 x g, 5 min, 4 ^{o}C). The supernatant (cytoplasmic fraction) was removed and placed into a fresh tube. The pellet (Nuclear fraction) was further lysed with Laemmli lysis buffer (see Cell Lysis). Lysates were analysed by western blot.

Immunoprecipitation.

Cells were lysed in immunoprecipitation lysis buffer (150 mM NaCl, 1 % NP40, 20 mM Hepes pH 7.5, 0.5 mM EDTA, 1 x protease inhibitor (Roche), 50 mM NaF, 10 mM β–glycerophosphate and 0.5 mM Sodium orthovanidate). Lysates were then cleared by centrifugation (20817 x g, 10 min, 4 °C). The protein concentration of the lysates was then determined by Bradford assay, such that an equal amount of protein was loaded into each immunoprecipitation. Protein G Dyna-beads (Millipore) were washed three times before indicated antibodies and lysate were added to beads and rotated at 4 °C for 3 hr. Beads were washed four times with immunoprecipitation wash buffer (150 mM NaCl, 1 % NP40, 20 mM Hepes pH 7.5, 0.5 mM EDTA) by centrifugation (2150 x g, 4 °C, 2 min) prior to resuspension in loading buffer (10 % Glycerol, 62.5 mM Tris-HCl pH 6.8, 2 % SDS, 2 % 2-Mercaptoethanol plus bromophenol blue).

Immunoprecipitation of Purified Proteins.

Recombinant His-tagged RASSF1A (1 μg) (Fitzgerald Industries, USA) was mixed with either recombinant, kinase active His-tagged CSK (1 μg) (Active Motif) or His-tagged SRC (1 μg) (Active Motif) in a reaction tube with mild lysis buffer (0.1% NP40, 20 mM HEPES pH 7.5, 150 mM NaCl, 0.5 mM EDTA, 50 mM NaF, 10 mM β–glycerophosphate and 0.5 mM Sodium orthovanidate) supplemented with protease inhibitors (Roche) in 20 μl volume. The tubes were then incubated for 30 minutes at 37 °C after which 5 μl of each reaction was used for immunoprecipitation. The pull-downs were achieved with either SRC (Cell Signalling) or RASSF1 (Santa Cruz) antibodies bound to agarose beads for 1.5 hours at 4 °C under constant rotation. The beads were washed four times with the lysis buffer by centrifugation (2150 x g, 4 °C, 5 min) prior to resuspension in loading buffer (10 % Glycerol, 62.5 mM Tris-HCl pH 6.8, 2 % SDS, 2 % 2-Mercaptoethanol plus bromophenol blue).

Immunofluorescence.

Cells, plated onto glass cover slips, were fixed for 15 min in 4 % paraformaldehyde (PFA) and permeabilsed with 0.2 % Triton X before blocking with 0.2 % Fish Skin Gelatin (FSG) for 1 hr. Primary antibody incubations were carried out at 4 °C overnight in 0.2 % FSG. Unless otherwise stated primary antibodies were diluted 1 in 100. Cover slips were washed three times with PBS before incubation with Alexa fluor secondary antibody (1 in 500) (Life Technologies). Cover slips were washed a further three times with PBS before being mounted onto slides with ProLong Gold antifade mounting medium with DAPI (Life Technologies). Slides were imaged using Nikon 90i microscope using NIS elements software, Leica DM IRBE microscope using Simple PCI6 software or Zeiss LSM780 confocal microscope using Zeiss ZEN2011 software.

Dispase Assay.

Cells were incubated in 2 μg/ml Dispase II solution (Sigma) for 30 min before junctions were disrupted mechanically by pipetting. Cells were then centrifuged (1 x g, 5 min) to pellet cell aggregates. Samples were taken from the top of the supernatant and analysed using a haemocytometer.

Scratch Wound Assay.

Cells were grown at high confluency on a tissue culture plate. A wound was made using a P200 pipette tip. Dislodged cells were removed by washing 3 times with PBS before media was re-added to the plate. HeLa cells were incubated in DMEM supplemented with 10 % serum for 24 hr. MDA-MB-231 cells were incubated in DMEM supplemented with 1 % serum for 14 hr. Images were taken at the same point in each plate at 0 hr and the end time point using the Nikon TE2000 Eclipse microscope and analyzed using NIS elements software. Transwell assay is described in supplementary methods.

Transwell Assay.

DMEM media containing 10 % FBS was loaded into the bottom chamber of a transwell plate (BD-Biosciences) before 2.5 x 10^4 MDA-MB-231 cells were loaded into the top chamber of a transwell plate in DMEM containing 0.1 % FBS. Cells were allowed to migrate at 37 ^{o}C, 5 % CO_2 for 18 hr in a humidified incubator before cells were fixed in 100 % methanol for 10 min at RT. Transwell membranes were removed from the bottom of the top chamber and mounted onto microscope slides using Prolong® Gold antifade reagent with DAPI (Life Technologies). Cells that had migrated through the membrane were then counted using a Nikon 90i microscope at 20 X magnification. Five fields of view were counted per membrane and three membranes were used in each experiment.

Calcium Switch Assay.

MCF7 cells were grown at high density (3 x 10^5 cells/condition) on cover slips in calcium containing media. With the exception of 'Control', cells were washed twice with PBS before being incubated overnight in calcium free media (Life Technologies). The cells were then washed in PBS and normal media (DMEM supplemented with 10 % FBS, 2 mM Glutamine and 100 U/ml Penicillin/Streptomycin) was added for up to 180 minutes. Coverslips were collected at the indicated time points. The coverslips were then stained using the immunofluorescence protocol. Images were taken using Ziess LSM780 using a 63 X objective and analysed using ZEN software. Cells expressing GFP-empty vector or GFP-RASSF1C were utilised for single cell analysis. Total contacts were calculated as the percentage of junctions formed vs the total number of cells in contact with cell being analysed.

Weak contacts were defined as being diffuse or interrupted staining at the cell-cell junction. Strong contacts were defined as robust, well defined staining at the cell-cell junction.

Mass Spectrometry.

Gel pieces excised from the SDS-PAGE gel were subjected to tryptic digest and the resulting peptides extracted from the gel following the *in-gel* digest protocol from the Kessler lab (www.ccmp.ox.ac.uk/protocols-and-tools). Briefly, samples were incubated overnight at RT in wash buffer (50 % (v/v) methanol, 5 % (v/v) acetic acid) before dehydration with acetonitrile. Samples were reduced (10 mM DTT, 10 min, RT) and alkylated (50 mM iodoacetamide, 10 min, RT) prior to digestion overnight at 37 ^{o}C with 20 μg/μl trypsin (Promega) (in 50 mM ammonium bicarbonate). Excess trypsin was removed and gel pieces incubated at 37 ^{o}C overnight. Trypsinised protein was extracted from the gel with extraction buffer 1 (50 % (v/v) acetonitrile, 5 % (v/v) formic acid, 10 min, RT) followed by extraction buffer 2 (85 % (v/v) acetonitrile, 5 % (v/v) formic acid, 10 min RT). Extracted peptides were analysed by online nanoflow liquid chromatography tandem mass spectrometry using a Dionex U300 (fitted with a Pepmap C18 column and eluted with a linear gradient of acetonitrile) connected to a Bruker HCTultra ETD II ion trap trough a nanoelectrospray ion source. Identified peptides were analysed using Mascot MS/MS ion search (www.matrixscience.com).

ExCELLigence Analysis.

160 μl DMEM containing 10 % FBS, with or without doxycycline (2 μg/ml) (SIGMA) were added to the bottom chamber of an exCELLigence CIM plate. The top chamber was then attached to the bottom chamber and 50 μl of DMEM without supplements was added to each well. The plate was loaded into the exCELLigence analyser and a blank run was done to zero the machine. 40,000 cells were plated into the top chamber and allowed to settle for 30 min at room temperature before the plate was loaded into the exCELLigence analyser. Cells were incubated in the machine at 37 ^{o}C, 5 % CO_2 in a humidified incubator and measurements were taken every 15 min for 25 hr. [S11]

Quantitative real time PCR (qPCR).

50,000 cells were lysed and cDNA prepared using the Power SYBR Green Cells-to-Ct Kit (Life Technologies). qPCR was done using the same kit on an Applied Biosystems 7500 Fast Real Time PCR system. Protocol: Holding Step (1 x cycle: 95 ^{o}C 10 min), Cycling Step (50 x cycle: 95 ^{o}C 15 sec, 60 ^{o}C 1 min) and Melt Curve Step (1 x cycle: 95 ^{o}C 15 sec, 60 ^{o}C 1 min, 95 ^{o}C 30 sec, 60 ^{o}C 15 sec). 18S was used as an internal control. Primers are listed in Table 1.

Western Blot.

Protein samples were loaded onto a gel and separated by SDS-PAGE. NuPAGE® precast gels (10 % or 4-12 %) (Life Technologies) were loaded into a Novex® mini cell tank filled with Novex® MOPS running buffer (both from Life Technologies). Self-cast gels made with a 10 % resolving gel and 4 % stacking gel and were loaded into BioRad Mini Protean® TetraCell gel tanks. Protein was transferred onto PVDF-F or PVDF-P using Bio-Rad mini gel blotting system. PVDF-P was blocked and incubated with primary with 5 % non-fat milk or BSA diluted in PBS-Tween 20. Secondary antibodies were always incubated in 5 % non-fat Milk. PVDF-P membranes were covered in ECL solutions from Thermo Scientific, Millipore or GE Healthcare prior to exposure to film (Kodak) and developed in a XoGraph developer. PVDF-F membranes were blocked and incubated with both primary and secondary antibodies in Licor blocking buffer (diluted 1:1 in PBS) prior to being analysed using the Licor Odyssey analyser. Membranes were quantitated using Image J or Licor Odyssey software. Primary antibodies: pLATS1 (Ser909), pLATS1 (Thr1079), YAP-pS127, phospho-MST1 (T183)/MST2 (T180), YES, SRC-pY416, SRC-pY527, SFK, c-SRC, E-cadherin and CSK were purchased from Cell Signalling Technologies; RASSF1A (3f3), TBX3 (A-20), TEAD2, β-catenin, YAP and CSK were purchased from Santa Cruz Biotechnology; TBX2 (62-2) (provided by Colin Goding); LATS1 (BETHYL Labs). RASSF1C, YAP-pY357, Fibrillarin and FAM120A (OSSA) were purchased from Abcam; FLAG-(M2)-tag, MYC-tag, HA-tag and anti-pTyrosine were purchased from Millipore, GAPDH and MST2 were purchased from Epitomics; p120-catenin, FAK and TEAD1were purchased from BD Biosciences; ZO-1 from Invitrogen.

Animal experiments.

Female SCID mice, 7-8 weeks old, were anaesthetized with 2-3 % isoflurane in 70 % N_2O:30 % O_2, placed in a stereotaxic frame (Stoetling Co., USA) and maintained with 1 % isoflurane. The skull was exposed and a burr-hole drilled. Three groups of animals were focally injected with either 5 x 10^3 MDA-MB-231 tumor cells untreated, expressing empty vector (pCDNA3) or MYC-RASSF1C, in 0.5 µl PBS in the left striatum (coordinates relative to Bregma; anterior +0.5 mm, lateral 1.5 mm, depth 2.5 mm) using a 75 mm-tipped glass microcapillary (Clark Electromedical Instruments, UK). At day 21 after intracerebral tumor cell injection all animals were transcardially perfusion-fixed under terminal anesthesia (n = 4 per group) with 0.9 % heparinized saline followed by 200 ml of periodate lysine paraformaldehyde (PLP) containing only 0.025 % glutaraldehyde (PLP*light*). The brains were post-fixed, cryoprotected, embedded and frozen in isopentane at -40 °C. Immunohistochemistry. For immunohistochemical analysis, 10 µm sections were collected onto gelatinized slides, washed in PBS

and counterstained for 5 min in cresyl violet. Slides were mounted and mounted using DPX (Thermo Fisher Scientific, UK). To assess areas of tumor colonization, photomicrographs of each brain section were obtained using ScanScope CS slide scanner (Aperio, Vista, CA, USA) and analyzed using ImageScope (Aperio). For immunofluorescence, sections were quenched with 1 % hydrogen peroxide in PBS, streptavidinand biotin-blocked (SP-2002, Vector Laboratories), blocked (TNB, PerkinElmer), incubated with anti-CD34 primary antibody (Abcam, UK, brain vessels) and anti-Vimentin antibody (VectorLabs, tumor cells), and secondary antibody (biotinylated anti-Rat and anti-Rabbit antibodies respectively). Sections were then washed with PBS, incubated with streptavidin-HRP (PerkinElmer; 1:200) in TNB for 30 min, washed and incubated for 8 min in the dark with TSA-biotin (PerkinElmer; 1:100) in amplification buffer (PerkinElmer). Slides were washed and incubated with a streptavidin-Cy3 fluorophore or AMCA-conjugated secondary antibody (Invitrogen; 1:100) for 30 min. Slides were cover-slipped using Vectashield mounting medium (Vector Laboratories).

RT-PCR primer sequences.

Target Gene	Forward Primer	Reverse Primer
BCL2L1[16]	ACTCTTCCGGGATGGGGTAA	ACAAAAGTATCCCAGCCGCC
BIRC5[16]	TGACGACCCCATAGAGGAACA	CGCACTTTCTCCGCAGTTTC
C-MYC[16]	CCTACCCTCTCAACGACAGC	CTTGTTCCTCCTCAGAGTCGC
CTGF	CACCCGGGTTACCAATGACA	GGATGCACTTTTTGCCCTTCTTA
RASSF1A	AGTGCGCGCATTGCAAGTT	AAAGGTCAGGTGTCTCCCAC
RASSF1C[26]	CTGCAGCCAAGAGGACTCGG	GGGTGGCTTCTTGCTGGAGGG
18S[30]	AGTCCCTGCCCTTTGTACACA	GATCCGAGGGCCTCACTAAAC

siRNA sequences.

Target Gene	siRNA target sequence	Purchased from
siRASSF1 (siGenomeSMARTpool)	ACGCACAAGGGCACGUGAA	Thermo (Dharmacon)
	CAAGGACGGUUCUUACACA	
	GCAAGAAGCCACCCUCCUU	
	CUACAUAACUUCCUACGUA	
siRASSF1A	GACCUCUGUGGCGACUUC	Eurofins MWG
siMST	GGAUAGUUUUUCAAAUAGGtt	Ambion
siOSSA (siGenome SMARTpool)	GCGUAUGACUCUGAUUAUG	Thermo (Dharmacon)
	GUUAUUCGAUUUAAGAGAG	
	AGGCAGCUGUCUUAAAUAA	
	GCUAUCAGCUCUCUUCAUG	
siRASSF1_1	ACGCACAAGGGCACGUGAA	Eurofins MWG
siRASSF1_2	CAAGGACGGUUCUUACACA	Eurofins MWG
siYAP1	CUGGUCAGAGAUACUUCUUtt	
siCTNNB1 (siGenome SMARTpool)	GCUGAAACAUGCAGUUGUA	Thermo (Dharmacon)
(b-catenin)	GAUAAAGGCUACUGUUGGA	
	CCACUAAUGUCCAGCGUUU	
	ACAAGUAGCUGAUAUUGAU	
siTEAD1 (siGenome SMARTpool)	Cat no. GS7003	Qiagen
siTEAD2 (siGenome SMARTpool)	GGAAGACCCGAACUCGAAA	Thermo (Dharmacon)
	GGAAUGAACUGAUCGCCCG	
	GCAGUUGAUUCUUACCAGA	
	CGAAGGAAAUCAAGGGAAA	
siTBX2	GUUUCACAACUCCCGCUGGUU	
siTBX3	CAGCTCACCCTGCAGTCCA	
siNT siGenome sequence No. 2	siGenome sequence No. 2	Thermo(Dharmacon)
siYES1	GAAGGACCCUGAUGAAAGA	Thermo(Dharmacon)
siSRC	GAGAACCUGGUGUGCAAAG	Thermo(Dharmacon)

Supplemental References

S1. **Scrace, S., O'Neill, E., Hammond, E.M., and Pires, I.M. (2013). Use of the xCELLigence System for Real-Time Analysis of Changes in Cellular Motility and Adhesion in Physiological Conditions. Methods Mol Biol *1046*, 295-306.**
