# Supplementary figures and images for: Alternate RASSF1 Transcripts Control SRC Activity, E-Cadherin Contacts, and YAP-Mediated Invasion
Source: Curr Biol. 2015 Dec 7;25(23):3019–34. doi: 10.1016/j.cub.2015.09.072 (PMC4683097; doi:10.1016/j.cub.2015.09.072)

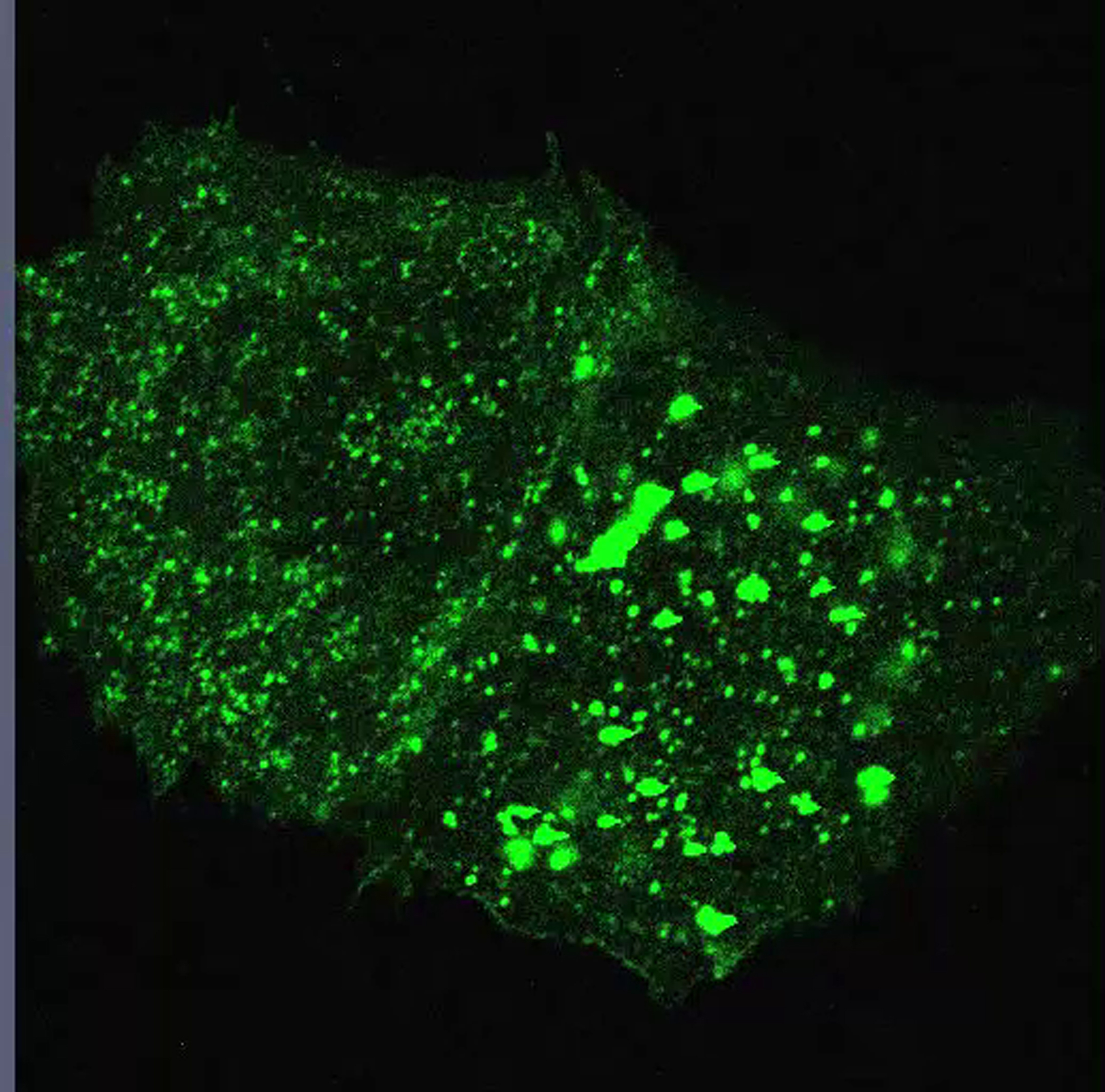

Supplement: Movie S1. Time-Lapse of the Movement of Internalized E-Cadherin — Left: control cell. Right: RASSF1C-expressing cell. Images were taken every second for 200 seconds. Video represents 100 seconds of time lapse. [file mmc2.jpg]

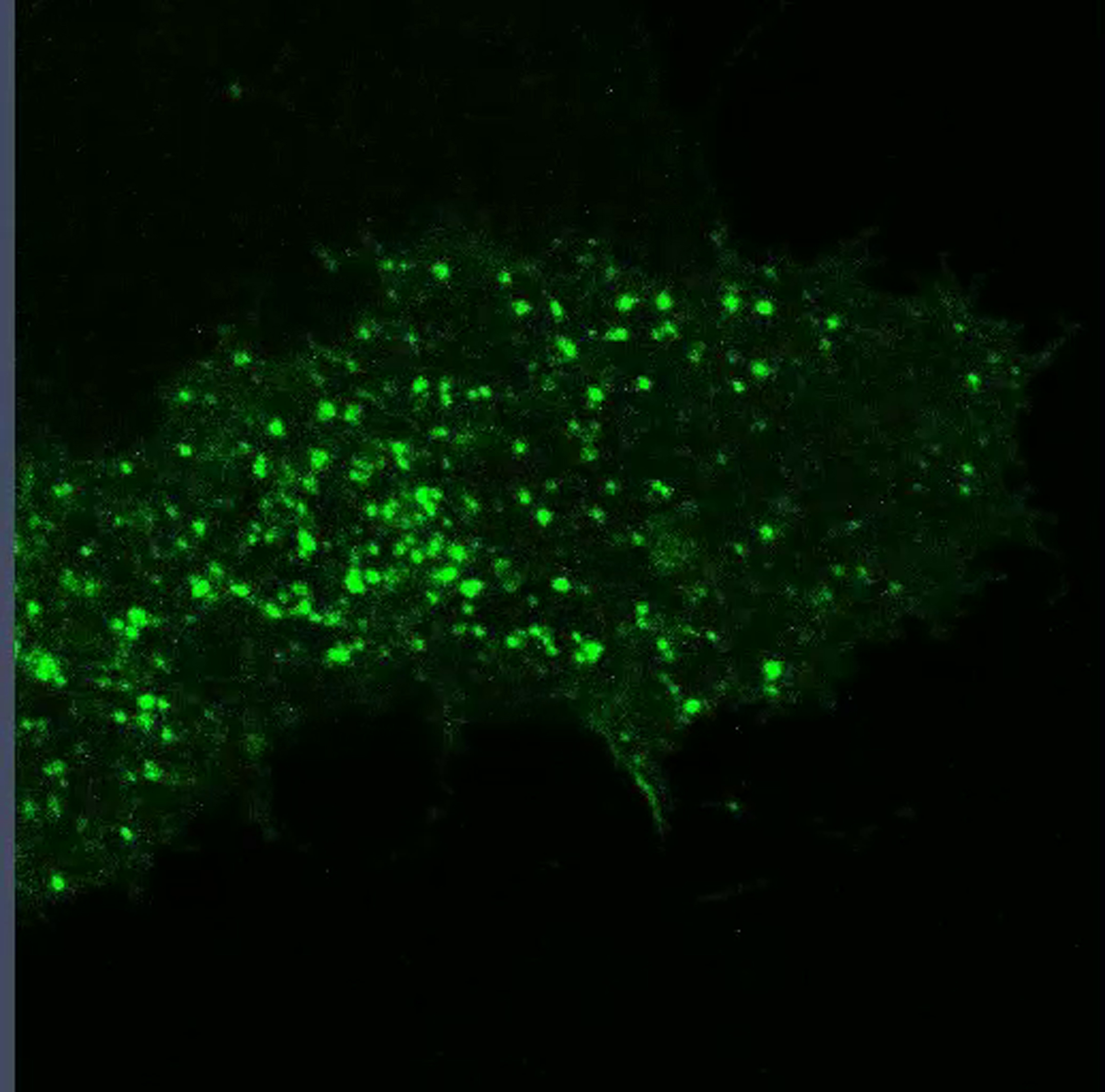

Supplement: Movie S2. Time-Lapse of the Movement of Internalized Control Cells Expressing Mutant E-Cadherin — Images were taken every second for 200 seconds. Video represents 100 seconds of time lapse. [file mmc3.jpg]

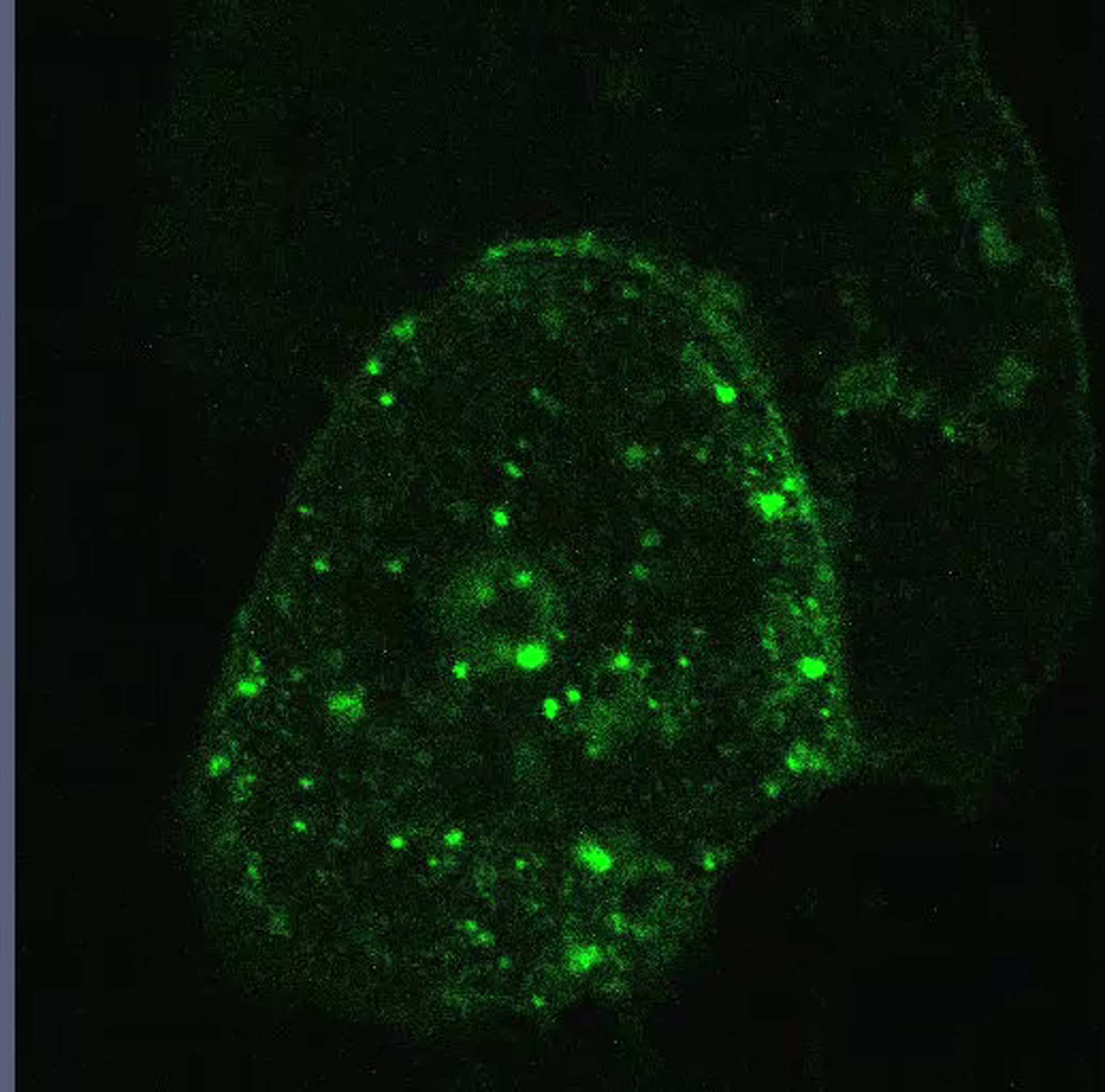

Supplement: Movie S3. Time-Lapse of the Movement of Internalized RASSF1C Cells Expressing Mutant E-Cadherin — Images were taken every second for 200 seconds. Video represents 100 seconds of time lapse. [file mmc4.jpg]

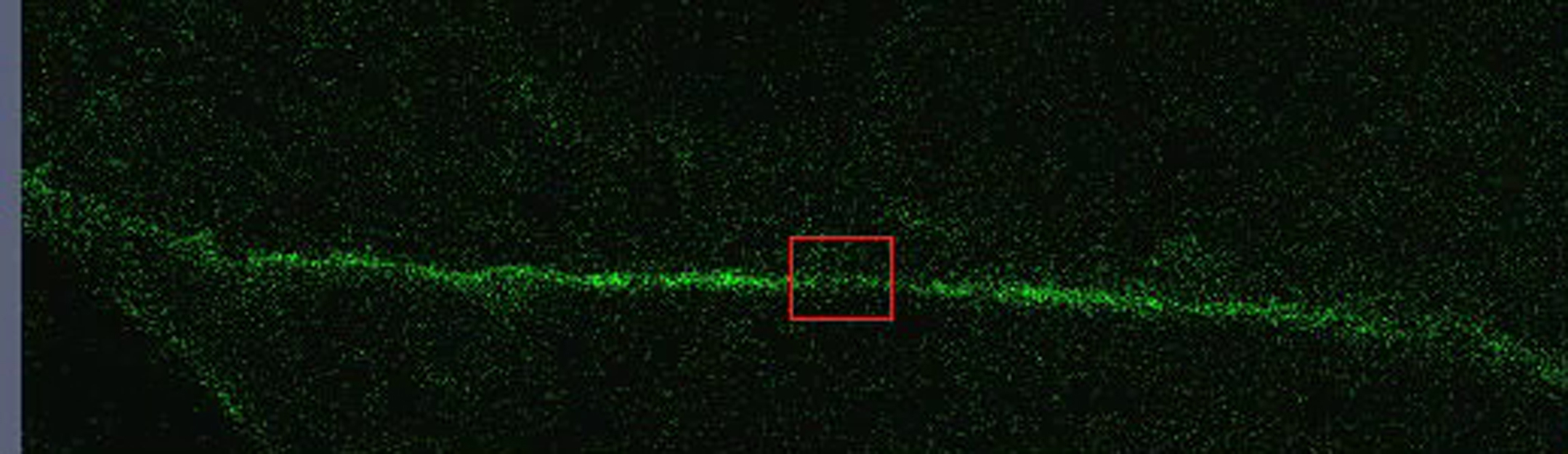

Supplement: Movie S4. Representative Video for the FRAP Analysis of Control Cells — Red rectangle: site of photobleaching. Video represents 3 min of time lapse made of 100 consecutive images taken. [file mmc5.jpg]

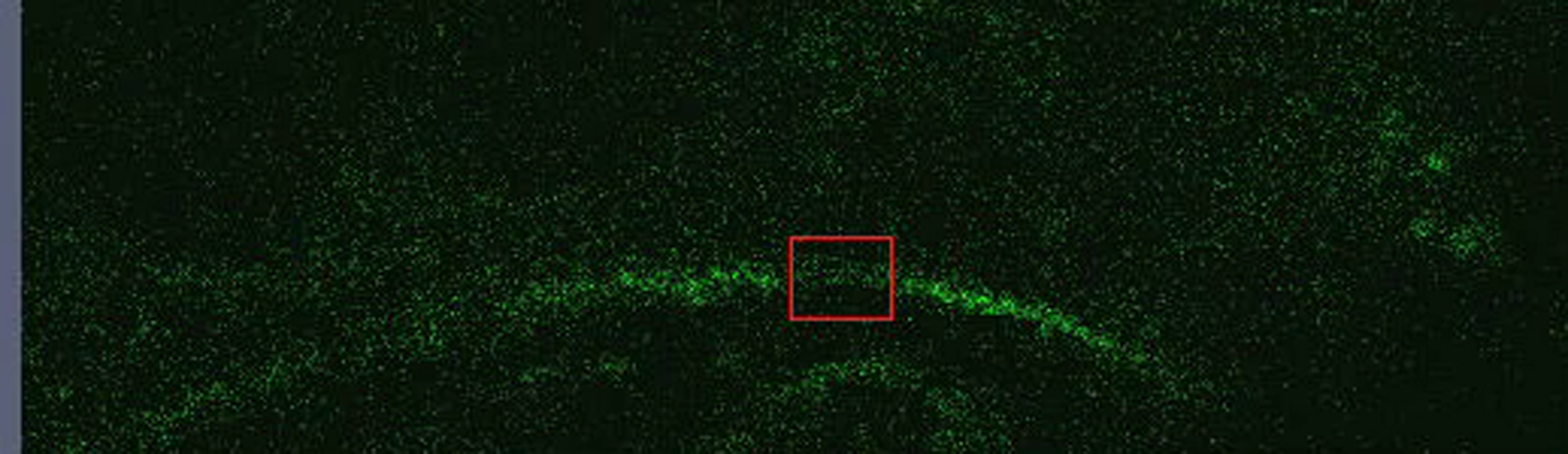

Supplement: Movie S5. Representative Video for the FRAP Analysis of RASSF1C Cells — Red rectangle: site of photobleaching. Video represents 3 min of time lapse made of 100 consecutive images taken. [file mmc6.jpg]
