# Supplementary material for: Alternate RASSF1 Transcripts Control SRC Activity, E-Cadherin Contacts, and YAP-Mediated Invasion
Source: Curr Biol. 2015 Dec 7;25(23):3019–34. doi: 10.1016/j.cub.2015.09.072 (PMC4683097; doi:10.1016/j.cub.2015.09.072)
Supplement: Document S2. Article plus Supplemental Information [file mmc7.pdf]

Current Biology

Article

Alternate RASSF1 Transcripts Control SRC Activity, E-Cadherin Contacts, and YAP-Mediated Invasion

Graphical Abstract

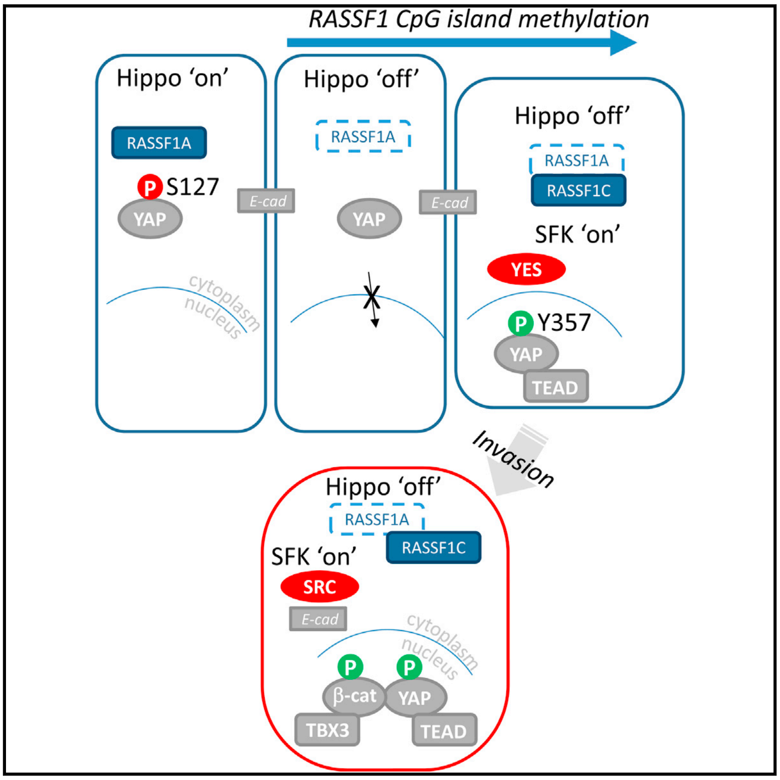

Authors

Nikola Vlahov, Simon Scrace, Manuel Sarmiento Soto, ..., Paul Timpson, Nicola Sibson, Eric O'Neill

Correspondence

eric.oneill@oncology.ox.ac.uk

In Brief

In a wide variety of sporadic malignancies, promoter methylation of the *RASSF1* gene is associated with tumor invasion and metastasis. Vlahov et al. show that the clinical phenotype is driven by both RASSF1A loss and the independently transcribed RASSF1C isoform, which promotes SRC activation, pseudo-EMT, and β-catenin/YAP1-dependent invasion of tumor cells.

Highlights

- Methylation of RASSF1A correlates with loss of hippo-inhibitory phospho-S127-YAP1
- SRC, FYN, and YES are activated by RASSF1C in RASSF1A-methylated cells
- RASSF1C promotes E-cadherin internalization and reduces cell junction integrity
- RASSF1A loss drives RASSF1C-YAP1/β-catenin-mediated transcription and invasion

Vlahov et al., 2015, Current Biology *25*, 3019–3034
December 7, 2015 ©2015 The Authors
http://dx.doi.org/10.1016/j.cub.2015.09.072

Current Biology
Article

CellPress

Alternate RASSF1 Transcripts Control SRC Activity, E-Cadherin Contacts, and YAP-Mediated Invasion

Nikola Vlahov,[1,5] Simon Scrace,[1,5] Manuel Sarmiento Soto,[1] Anna M. Grawenda,[1] Leanne Bradley,[1] Daniela Pankova,[1] Angelos Papaspyropoulos,[1] Karen S. Yee,[1] Francesca Buffa,[2] Colin R. Goding,[1,4] Paul Timpson,[3] Nicola Sibson,[1] and Eric O'Neill[1,*]
[1]CRUK/MRC Oxford Institute
[2]Applied Computational Genomics Group
Department of Oncology, University of Oxford, Oxford OX3 7DQ, UK
[3]Faculty of Medicine, Garvan Institute of Medical Research, University of New South Wales, Darlinghurst, NSW 2010, Australia
[4]Ludwig Institute for Cancer Research, University of Oxford, Oxford OX3 7DQ, UK
[5]Co-first author
*Correspondence: eric.oneill@oncology.ox.ac.uk
http://dx.doi.org/10.1016/j.cub.2015.09.072
This is an open access article under the CC BY license (http://creativecommons.org/licenses/by/4.0/).

SUMMARY

Tumor progression to invasive carcinoma is associated with activation of SRC family kinase (SRC, YES, FYN) activity and loss of cellular cohesion. The hippo pathway-regulated cofactor YAP1 supports the tumorigenicity of RAS mutations but requires both inactivation of hippo signaling and YES-mediated phosphorylation of YAP1 for oncogenic activity. Exactly how SRC kinases are activated and hippo signaling is lost in sporadic human malignancies remains unknown. Here, we provide evidence that hippo-mediated inhibition of YAP1 is lost upon promoter methylation of the RAS effector and hippo kinase scaffold RASSF1A. We find that RASSF1A promoter methylation reduces YAP phospho-S127, which derepresses YAP1, and actively supports YAP1 activation by switching *RASSF1* transcription to the independently transcribed RASSF1C isoform that promotes Tyr kinase activity. Using affinity proteomics, proximity ligation, and real-time molecular visualization, we find that RASSF1C targets SRC/YES to epithelial cell-cell junctions and promotes tyrosine phosphorylation of E-cadherin, β-catenin, and YAP1. RASSF1A restricts SRC activity, preventing motility, invasion, and tumorigenesis in vitro and in vivo, with epigenetic inactivation correlating with increased inhibitory pY527-SRC in breast tumors. These data imply that distinct *RASSF1* isoforms have opposing functions, which provide a biomarker for YAP1 activation and explain correlations of *RASSF1* methylation with advanced invasive disease in humans. The ablation of epithelial integrity together with subsequent YAP1 nuclear localization allows transcriptional activation of β-catenin/TBX-YAP/TEAD target genes, including Myc, and an invasive phenotype. These findings define gene transcript switching as a tumor suppressor mechanism under epigenetic control.

INTRODUCTION

Recent advances have highlighted that YES-associated protein (YAP1) supports KRAS tumorigenicity and assists in the maintenance of transformed phenotypes [1]. YAP1 drives proliferation by acting as a cofactor for TEAD transcriptional regulators, an activity which is restricted by hippo pathway-mediated disruption of TEAD association. In model systems, genetic ablation of core hippo pathway components leads to increased tumorigenesis [1]. In human tumors, failure to activate LATS1 due to either GNAQ mutations in uveal melanoma or through inactivation of NF2/merlin in the tumor-prone neurofibromatosis syndrome prevent this inhibitory signal and make YAP1 permissive for activation [1]. Similarly, stromal mechanics and genetic instability are reported to trigger the hippo pathway and present a tumor barrier, but as with GNAQ mutations and germline defects in NF2/merlin, these mechanisms appear to be independent of the hippo kinase/MST itself [1]. Identification of the core hippo pathway by proteomics has revealed the main direct activators of MST kinases to be SAV1 and RASSFs [2], which although infrequently mutated in cancers [3] have germline and epigenetic alterations, particularly in RASSF1A, that accelerate tumor onset and increase tumorigenicity [4, 5]. Moreover, RASSF1A activation of the hippo pathway both restricts YAP1 binding to TEAD [6] and is a direct substrate of RAS signaling in the pancreas [7], supporting the potential crosstalk in pancreatic tumor development [8, 9]. Intriguingly, methylation of the CpG island (CGI) spanning the promoter and first exon of *RASSF1* has widespread prognostic value for disease-free and poor overall survival in all major sporadic cancers [10]. Thus, loss of RASSF1A expression in *RASSF1*-methylated tumors is likely to contribute to reduced hippo pathway-mediated repression of YAP1, due to direct activation of MST kinases and the subsequent interaction and activation of LATS1 [4, 11–14]. This RASSF1A/MST2-promoted LATS1 activity is targeted toward YAP1 [12], resulting in increased pS127-YAP [15] and decreased YAP-TEAD oncogenic behavior [6, 15].

Hahn and colleagues recently highlighted that, by using a YAP1 mutant for all LATS1 phosphorylation sites (5SA) including the inhibitory S127-YAP1 phosphorylation, while permissive, required additional phosphorylation of Y357-YAP1 by the SRC

CellPress

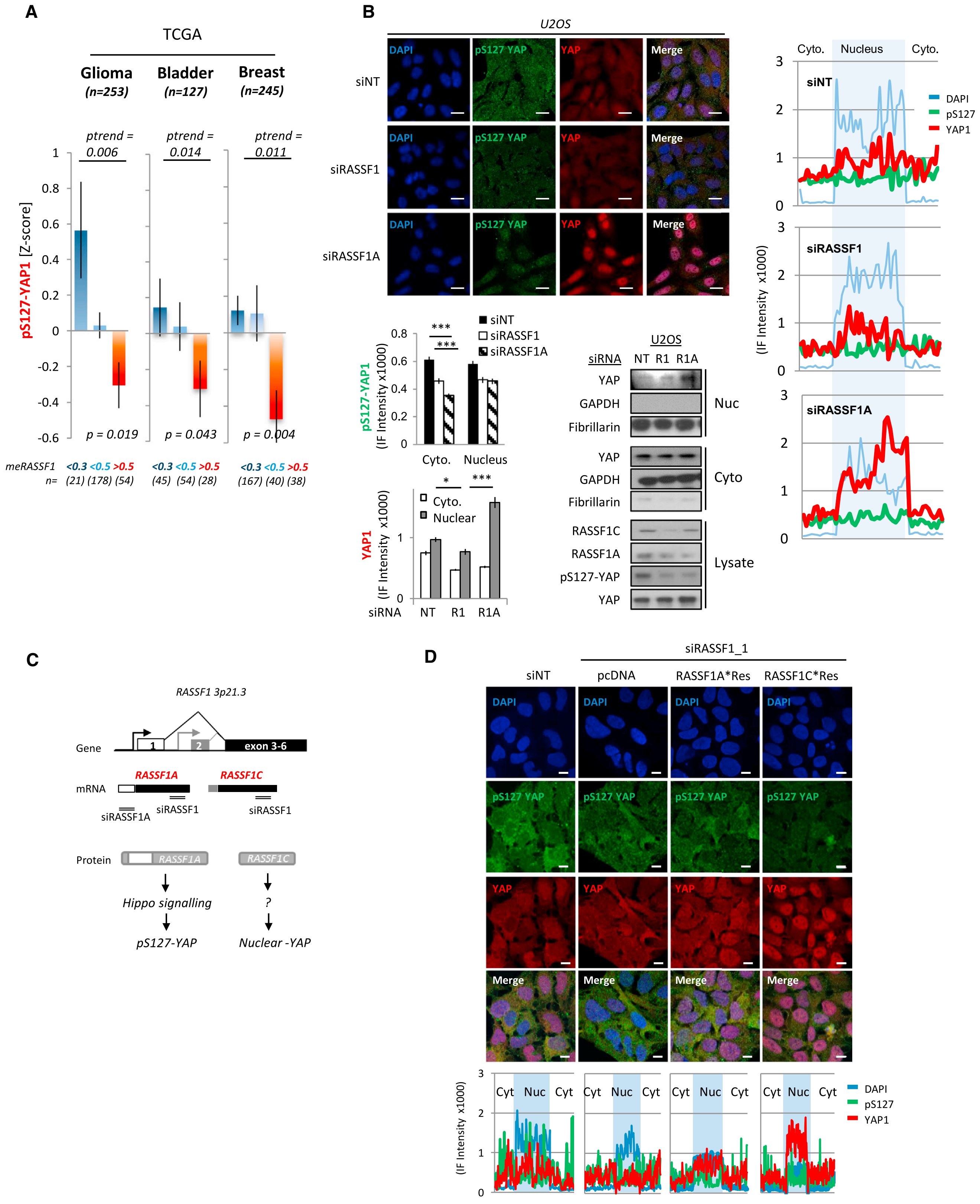

Figure 1. Loss of RASSF1A Mediates Nuclear Localization of YAP1

(A) Correlation of RASSF1 methylation levels (meRASSF1) below 0.3, between 0.3 and 0.5, and above 0.5 with loss of inhibitory YAP1-phospho-Ser127 (pS127-YAP1) in the cancer genome atlas (TCGA) data sets (cBioportal).

(legend continued on next page)

family kinase (SFK) YES to activate transcription [16]. Moreover, they found that the main tumorigenic consequence of APC loss in colorectal cancer was due to a subset of β-catenin genes that were YAP1 dependent. Thus, YAP1 may serve to integrate WNT and RAS signaling in order to trigger epithelial-to-mesenchymal transition (EMT) and invasive disease [17]. Tumor progression to invasive carcinoma is associated with constitutive loss of cellular cohesion, leading to EMT, increased cellular motility, and invasion into the surrounding tissues [18]. Activation of SFKs is associated with increased tumor invasion and metastasis through initial destabilization of epithelial cell-cell junctions. Mechanistically, this occurs through direct phosphorylation of E-cadherin, p120-catenin, and β-catenin [19–21]. Phosphorylation of E-cadherin promotes internalization, whereas phosphorylation of β-catenin decreases its affinity for E-cadherin, allowing nuclear localization where it serves as a transcriptional co-activator of TCF/Lef1 factors [22, 23]. The identification of a YAP1-β-catenin transcriptional program specified a novel role for β-catenin in activating transcription via T-box factors rather than TCF, particularly in cancers [16]. Importantly, YAP1 has also been shown to cooperate with β-catenin at TEAD-dependent promoters to induce cell proliferation in the heart and during tumorigenesis [24].

Here, we identify that *RASSF1* CGI methylation correlates with hippo pathway inactivation and loss of pS127-YAP1 in sporadic malignancies and provide evidence for the association of this methylation with invasive behavior in cancers, such as breast, bladder, and in gliomas. CGI methylation restricts primary transcript expression but can additionally promote switching to alternative gene promoters or influence splicing, implying that epigenetic regulation may modulate the relative levels of gene isoforms rather than simply silence gene expression [25]. In tumors with *RASSF1* CGI methylation, an alternative isoform, RASSF1C, is expressed from an internal promoter and has been suggested to promote motility and invasive disease [26, 27]. We find that RASSF1C actually supports tumorigenesis by promoting SRC/YES-mediated phosphorylation of E-cadherin, β-catenin, and YAP1, disrupting cell-cell contacts and initiating an EMT-like response. RASSF1A also binds to SFKs but additionally scaffolds CSK, potentially through an exon 1α-encoded C1 domain, not present in RASSF1C, which inhibits SFKs and maintains epithelial integrity. In the absence of RASSF1A, RASSF1C promotes tyrosine phosphorylation of β-catenin and YAP1, resulting in their re-localization to the nucleus and transcriptional activation of the TBX target genes, BCL2L1, BIRC5 [16], and cMyc [28]. Analyses of invasive breast tumor data sets indicate an inverse correlation of high RASSF1A methylation/low pS127-YAP1 with SRC activation and the expression of invasion-associated transcripts. To validate a role in motility and invasion, we demonstrate that RASSF1C directly promotes SFK-dependent motility, 3D invasion of mammospheres and tumor spread in vivo. These data imply that SFK activation/inactivation relies on distinct RASSF1 isoforms, presenting a mechanism for YAP1 activation in sporadic tumors and explains the clinical correlation of *RASSF1* methylation with advanced invasive disease.

RESULTS

Switching of RASSF1 Isoforms Induces Nuclear Localization of YAP1

RASSF1A is a hippo pathway scaffold that switches YAP1 association from oncogenic TEAD transcriptional complexes to tumor-suppressive YAP1/p73 [6]. As RASSF1A expression is lost in multiple cancers and associates with poor outcome, we wanted to determine whether this was a route through which the hippo pathway may be inactivated in sporadic cancers. To address this, we explored YAP1 protein information in data sets of tumors where *RASSF1* is known to be methylated, clinically significant [10], and for which pS127-YAP information was available. We found that methylation of *RASSF1-1α* (representing gene silencing) significantly correlates with low pS127-YAP1 in glioma, bladder, and breast cancer cohorts (Figure 1A), suggesting that YAP1 may be nuclear and active in *RASSF1*-methylated tumors. To test this correlation, we targeted RASSF1A expression in U2OS cells, unmethylated for RASSF1A, with siRNAs and observed lower pS127-YAP1 in line with the clinical data (Figures S1A and S1C). Intriguingly, in contrast to siRNA targeting exons common to all isoforms (siRASSF1), specific ablation of the RASSF1A isoform resulted in elevated nuclear localization of YAP1, indicating that reduced pS127-YAP1 appears required but insufficient for nuclear localization of YAP1 (Figure 1B). In keeping with a loss of hippo pathway activity, the loss of RASSF1 or RASSF1A led to a decrease in the phosphorylation of the core hippo kinases MST1/2 and LATS1 (Figure S1C) and was conversely increased by overexpression of RASSF1A in both U2OS and H1299 (methylated) cells (Figure S1D). RASSF1C transcripts are often present in RASSF1A-methylated tumors due to expression from a distinct promoter and are susceptible to siRASSF1, but not siRASSF1A (Figures 1C, S1B, and S1C) [27]. To determine whether the RASSF1C transcript was responsible for elevated nuclear YAP1 upon siRASSF1A, we designed derivatives of RASSF1A or RASSF1C to be resistant

(B) Immunofluorescence detection of YAP1 and pS127-YAP1 in U2OS cells transfected with siNT, siRASSF1A, and siRASSF1 (top). (Bottom left) Using vectors depicted in Figure S1, histogram indicating the comparative nuclear and cytoplasmic levels of YAP1 (bottom) and pS127 (top) determined by intensity of the immunofluorescence distribution is shown. (Bottom right) The corresponding immunoblot showing the nuclear, cytoplasmic, and total lysate levels of YAP1 is shown. (Far right) Distribution of DAPI (blue), YAP1 (red), and pS127-YAP1 (green) across equatorial cell vector determined by immunofluorescence is shown (average of n = 29).

(C) Schematic representation of the RASSF1A and RASSF1C isoforms (top), the location of the sequence for the siRNAs used (middle), and protein functions (bottom).

(D) Immunofluorescence detection of YAP1 and pS127-YAP1 in U2OS cells transfected with siNT, siRASSF1, and siRNA-resistant versions of RASSF1A and RASSF1C as indicated. Images are representative of two independent siRASSF1 oligos to which two siRNA restraint isoforms of both RASSF1A and RASSF1C were designed (see Figures S1G and S1H). Distribution of DAPI (blue), YAP1 (red), and pS127-YAP1 (green) across equatorial cell vector determined by immunofluorescence is shown (bottom).

All scale bars represent 20 μm.

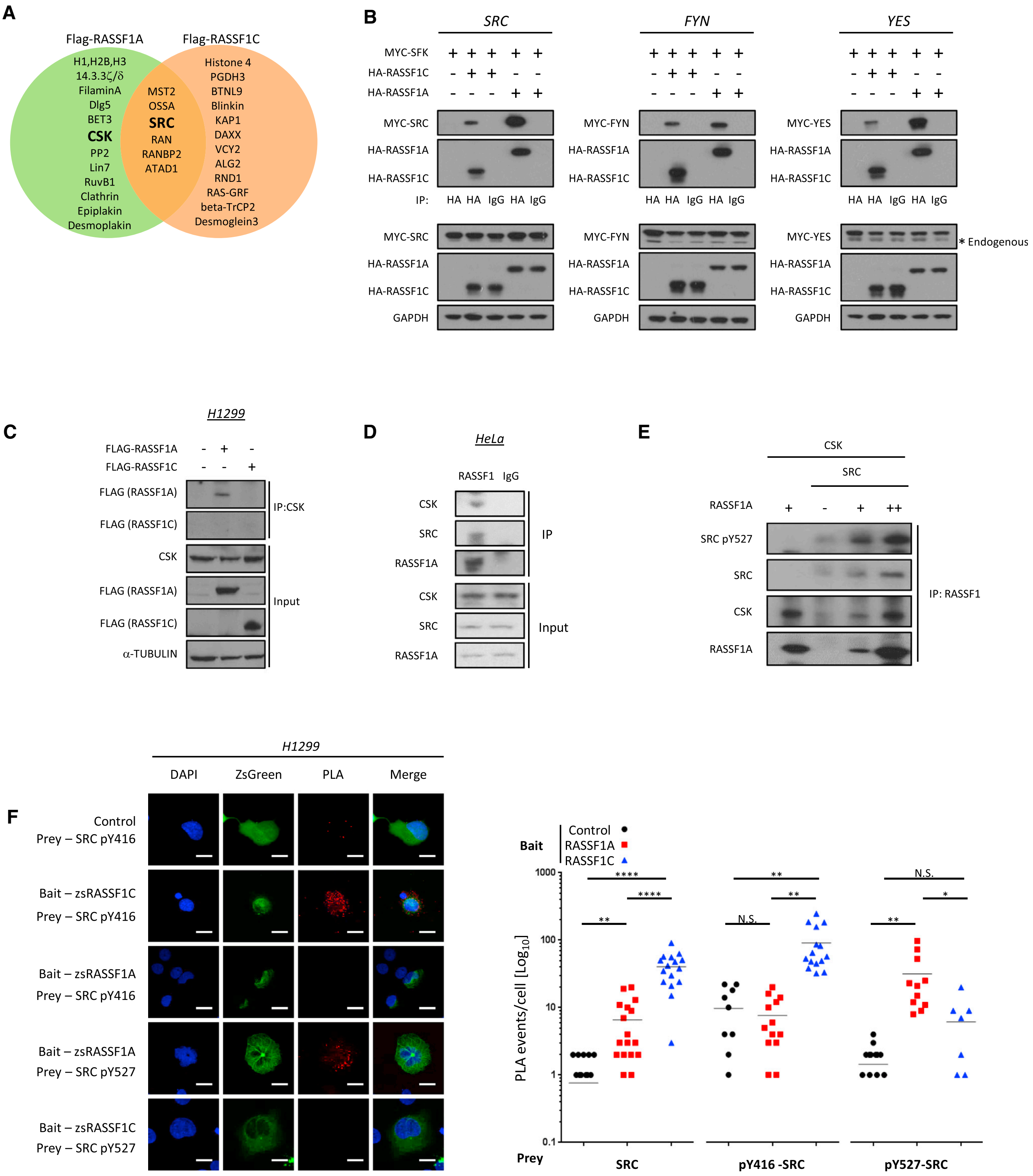

Figure 2. RASSF1C Binds to and Activates SFKs

(A) Proteomic identification of RASSF1A and RASSF1C immunoprecipitates by tandem MS/MS.

(B) HA-tag immunoprecipitation from 293T cells transfected with MYC-SRC (left), MYC-FYN (middle), or MYC-YES (right) in combination with HA-RASSF1A or HA-RASSF1C, analyzed by immunoblotting.

(C) CSK immunoprecipitation from H1299 cells transfected with FLAG-RASSF1A or FLAG-RASSF1C. The precipitates along with input fractions were analyzed by immunoblotting.

(D) RASSF1 immunoprecipitation from HeLa cells, immunoblotted for CSK, SRC, and RASSF1A.

(legend continued on next page)

to the two distinct RASSF1 siRNAs, allowing a direct comparison. RASSF1C expression restored nuclear YAP1 in siRASSF1-transfected cells without affecting the pS127-YAP1 levels, whereas RASSF1A failed to do so (Figures 1D, S1E, S1G, and S1H). Thus, reduced hippo signaling and pS127-YAP levels appear effectively uncoupled from automatic nuclear localization, in keeping with the recently demonstrated requirement for tyrosine phosphorylation to mediate the transition [29]. Intriguingly, phosphotyrosine immunoprecipitates of U2OS cells demonstrate that RASSF1C promotes increased tyrosine phosphorylation of YAP1 (Figure S1F). This indicates that promoter methylation of *RASSF1-1*α, which inhibits RASSF1 isoform A expression, reduces hippo signaling and inhibitory pYAP-S127 but favors the nuclear localization of YAP1 via *RASSF1-1*α-independent transcription of RASSF1 isoform C.

RASSF1 Isoforms Interact with and Differentially Regulate SFKs

To determine the mechanism of how RASSF1C promotes tyrosine phosphorylation and nuclear localization of YAP, we performed a proteomic screen of both isoforms to identify novel protein-protein interactions (Figure 2A). We screened RASSF1 immunoprecipitates for candidates and found that both RASSF1 isoforms bind the tyrosine kinases c-SRC, FYN, and YES (Figure 2B); interestingly, however, RASSF1A had an additional unique association with the SRC inhibitory kinase, CSK (Figures 2C and S2G). The binding of endogenous RASSF1A to CSK was confirmed in HeLa cells, unmethylated for RASSF1A (Figure 2D), and could also be demonstrated to be direct using bacterially purified proteins for RASSF1A, SRC, and CSK (Figure 2E). This association was in line with a similar role for the homolog dRASSF8 in *Drosophila* [30]. To further confirm association, we employed proximity ligation assays and found that, whereas both isoforms bind SRC, RASSF1C associated with active pY416-SRC whereas RASSF1A bound inactive, CSK-phosphorylated, pY527-SRC (Figures 2F and S2A). We next investigated the effect of RASSF1 isoform modulation of SFK activity. Depletion of RASSF1A alone had no effect on SRC activity, whereas siRASSF1 led to reduced pY416-SRC in serum or HGF-stimulated cells (Figures S2B and S2C). Conversely, exogenous expression of RASSF1C elevated pY416-SRC in *RASSF1*α-methylated colorectal, breast, and lung cancer cells but failed to do so in unmethylated cells where RASSF1A is expressed (Figures S2D and S2E) [4]. We therefore hypothesized that RASSF1C may activate SRC but is restricted by competition for association with higher-affinity RASSF1A and associated CSK. To test this, we expressed RASSF1C in H1299 cell lines in which RASSF1A expression was inducible [4] and observed increased pY416-SRC only in the absence of RASSF1A (Figure S2F). Taken together, the data indicate that RASSF1A and RASSF1C modulate SRC activity by promoting differential phosphorylation of SRC.

RASSF1C Targets SRC to the Plasma Membrane, Destabilizing Junctions

Once activated, SRC translocates to the cell membrane and phosphorylates key target proteins [31]. To determine whether the activation of SRC by RASSF1C affects the localization, we tracked GFP-SRC and pY416-SRC by florescence microscopy. We found that increased pY416-SRC levels in cells correlated with localization of RASSF1C, endogenous SRC, and pY416-SRC at the membrane (Figures S3A and S3B), with a significant increase at cell junctions compared to non-junctional plasma membrane (Figures 3A and 3B; red versus white arrows). Phosphotyrosine immunoprecipitates suggest that RASSF1C promotes phosphorylation of junction proteins E-cadherin and β-catenin, but not p120-catenin or FAK (focal adhesion kinase) (Figure 3C). SRC phosphorylation of E-cadherin is known to increase its internalization, subsequently destabilizing cell-cell junctions [19]. To determine whether RASSF1C-promoted phosphorylation of E-cadherin has any effect on E-cadherin junctional integrity, we first took GFP-E-cadherin-expressing cells where E-cadherin can be visualized at the cell periphery (Figure 3D). Co-expression of RASSF1C decreased intensity of GFP-E-cadherin at cell-cell junctions compared to controls but did not affect a GFP-E-cadherin derivative harboring mutations in all three SRC phosphorylation sites Y753F, Y754F, and Y755F [32] (Figure 3D). Moreover, the destabilization of eGFP-E-cadherin by RASSF1C occurs in a SRC-dependent manner (Figure S3C). It has been previously shown that loss of junctional components like α-catenin [33, 34] or E-cadherin [35] leads to YAP1 nuclear localization. Therefore, we tested whether the loss of adherens junctions in cells expressing RASSF1C similarly leads to YAP1 nuclear localization and indeed found RASSF1C promotes nuclear YAP1, but not in cells expressing E-cadherin with the SRC phosphorylation sites mutated (Figure 3E). The fidelity of E-cadherin-mediated junctions relies on continuous recycling via internalization and replacement through the late endosomal compartment [36]. Visualization of E-cadherin endosome trafficking in real-time via 4D tracking software (Imaris Bitplane; ANDOR) indicated that RASSF1C-expressing cells had reduced trafficking speed and did not register movement toward the junction, supporting the idea of increased internalization and failure to recycle (Figures 4A and 4B; Movie S1), a phenomenon which again was not observed in the case of the E-cadherin mutant (Figure S4A; Movies S2 and S3). To further determine the E-cadherin stability at cell-cell junctions, we expressed GFP-E-cadherin and monitored its dynamics by FRAP analysis, as has been shown previously [37]. We found that expression of RASSF1C increased the mobile fraction of E-cadherin and its turnover at junctions (increased half-life [t 1/2]), but not upon inhibition of SRC with dasatinib (Figures 4C and S4B; Movies S4 and S5), suggesting that RASSF1C increased E-cadherin recycling via SRC, thus creating junctions that are less molecularly stable. To determine the physical effect on cell-cell adhesion, we employed a dispase assay and observed that RASSF1C expression

(E) Purified protein interaction and immunoprecipitation of GST-tagged CSK, SRC, and RASSF1A.

(F) Representative immunofluorescence images showing the different levels of colocalization of RASSF1A and RASSF1C (baits) with pY416-SRC and pY527-SRC (preys) using proximity ligation assay (Duolink) in H1299 cells (left). Graph showing the quantification of the events per cell is shown (right). All scale bars represent 20 μm.

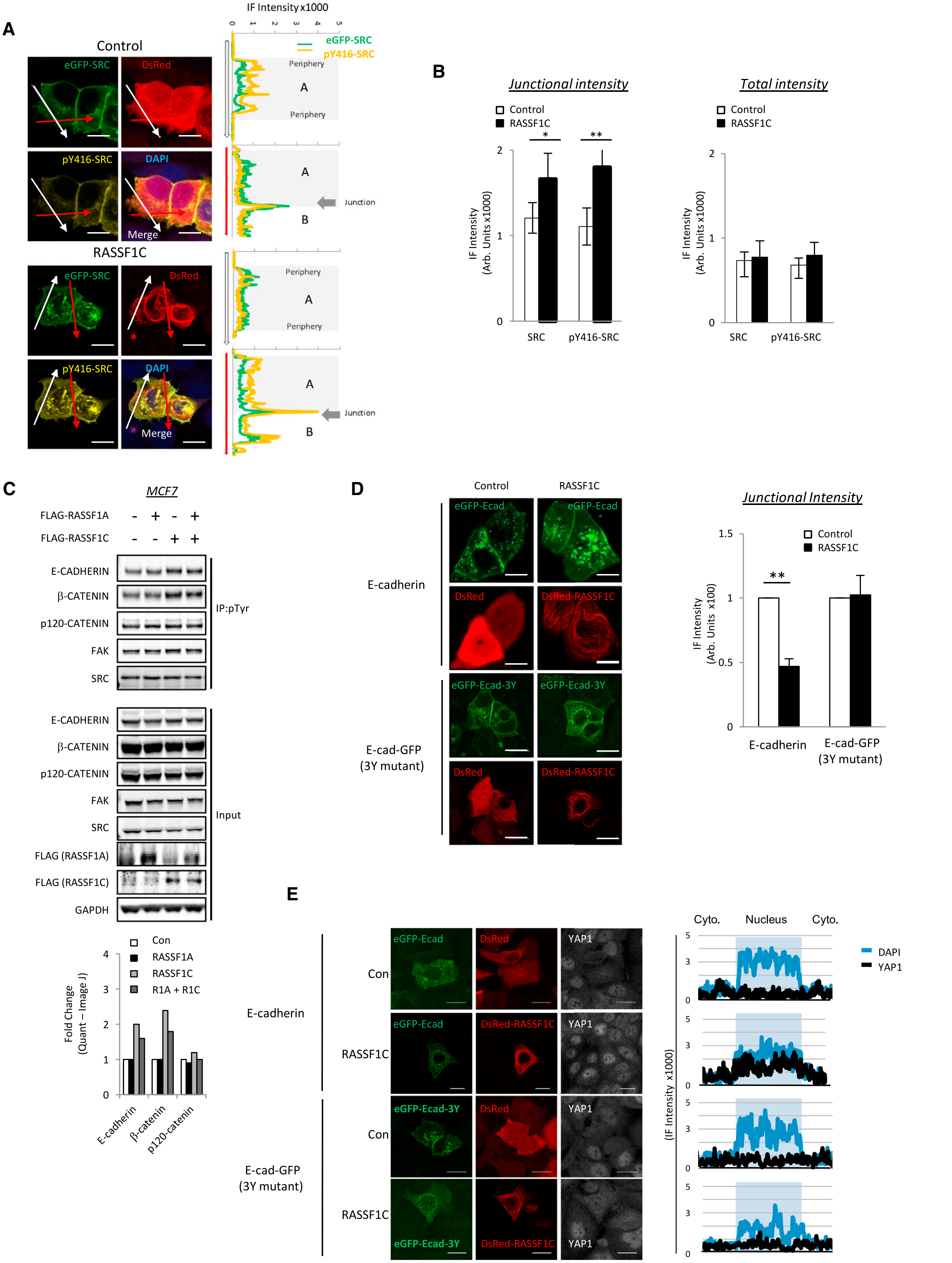

(legend on next page)

weakened cellular cohesion in a SRC-dependent manner (Figure 4D). As E-cadherin-mediated adherence relies on Ca^{2+} for stable contacts, we next wanted to test whether cell-cell disruption was indeed due to E-cadherin by employing a Ca^{2+}-switch assay. We found that, after E-cadherin contacts were efficiently ablated by removal of Ca^{2+} from the media, RASSF1C-expressing cells failed to efficiently form mature contacts upon Ca^{2+} replenishment (Figures 4E, S5A, and S5B). The siRNA knockdown of both SRC and YES endowed resistance to RASSF1C expression, implicating both in RASSF1C-mediated loss of junctional strength (Figure 4F, right) and E-cadherin intensity (Figure 4F, left). Taken together, the results indicate that expression of RASSF1C destabilizes cell-cell junctions and disrupts further recycling of E-cadherin via SRC. The loss of adherens junctions can have a wide effect on the cell. Therefore, we next wanted to further investigate the effect on cells after the loss of E-cadherin.

RASSF1C Promotes YAP and β-Catenin-Dependent Transcription

Strong E-cadherin contacts require β-catenin, which is protected from degradation through its interaction with E-cadherin [38]. RASSF1C expression promotes the phosphorylation of β-catenin (Figure 3C). Moreover, SRC phosphorylation of β-catenin decreases its affinity for E-cadherin, leading to dissociation from the membrane [22, 23]. This allows nuclear entry and transcription of target genes. Nuclear-cytoplasmic fractionation and immunofluorescence analysis confirmed that either RASSF1C expression (in RASSF1A-methylated H1299 and MCF7 cells) or specific loss of endogenous RASSF1A (U2OS and HeLa cells) increases nuclear β-catenin (Figures 5A–5C, S6A, and S6B). We also found that RASSF1C binds and activates YES1 (Figures 2B and 5D), known to promote YAP1 tyrosine phosphorylation that is required for nuclear localization and accumulation of a β-catenin-YAP complex [16, 39]. RASSF1C-induced nuclear YAP1 is also phosphorylated on Y357 (Figures 5D and 5E). Moreover, in MDA-MB-231 mesenchymal breast cancer cells that lack E-cadherin junctions, expression of RASSF1C is sufficient to drive nuclear localization of β-catenin and YAP1 (Figure S6C). β-catenin/YAP1 utilizes the T-box transcription factor TBX5 to drive a pro-tumorigenic transcriptional program including BCL2L1 and BIRC5 [16]. TBX5 is primarily linked to congenital heart disease; however, the homologs TBX2 and TBX3, which share DNA binding consensus with TBX5, are associated with cancer and invasive cellular behavior [40]. In line with the formation of a YAP1/β-catenin/TBX complex, we found that loss of RASSF1A, or expression of RASSF1C, similarly induced BCL2L1 and BIRC5 (BCL-xl and survivin; Figures 6A, 6B, S6A, and S6D).

Interestingly, RASSF1C also induces *cMYC* (Figures 6A, 6B, and S6A), which is a recognized transcriptional target of β-catenin [41] and YAP/TEAD1 [42] and is bound by TBX3 [28]. Thus, nuclear YAP1/β-catenin may be directed to a set of survival and invasive genes via TEAD1/TBX3 transcription factors. The induction of BCL2L1, BIRC5, and MYC expression by RASSF1C was reduced by siRNAs targeting either β-catenin/TBX3 or YAP1/TEAD1, suggesting that the YAP1/β-catenin complex is recruited via both TBX3 and TEAD1 DNA binding (Figures 6B and S6D). A reduction in BCL2L1, BIRC5, and MYC expression was also observed in RASSF1C cells treated with SRC inhibitors, further implicating that this process is SFK dependent (Figure S6E) [43]. MYC, as with SRC, is associated with invasive growth [44]. In agreement with activation of both oncogenes, RASSF1C promoted motility and invasion and was antagonized by RASSF1A, independently of other SRC activators identified in our screen (Figures 6C, 6D, and S6F–S6J). This was not a defect of attachment or spreading as increased invasion followed induction of RASSF1C expression in attached H1299 cells (Figure 6E, left) and is dependent on β-catenin, YAP, SRC, and YES (Figure 6E, right). Together, the data suggest that loss of RASSF1A promotes invasion via coordinated elevation of pY357-YAP1 and reduced pS127-YAP1, leading to transcriptional activation of specific TEAD/TBX3 genes.

Expression of RASSF1C Promotes Invasiveness and Tumorigenesis

To investigate the clinical relevance, we interrogated the databases described above (Figure 1A) for correlations between pS127-YAP1^{low} and invasive signatures using total YAP1 levels and the mSigDB database YAP-TAZ signature as a control. We assessed the extent of increased gene expression from invasive, metastatic, or EMT signatures but failed to see any highly significant correlation ($p < 0.0001$).

We next examined a second breast data set where methylation of RASSF1A was confirmed in all cases, suggesting that RASSF1C could be expressed and that loss of pS127-YAP1 can now combine with RASSF1C-promoted pY357-YAP1 to allow YAP1/β-catenin nuclear localization. In this data set, pS127-YAP1^{low} (independent of total-YAP1) did indeed have significantly more genes from invasive, metastatic, and EMT signatures (POOLA $p = 1.09e^{-78}$; BIDUS $p = 4.52e^{-11}$; ANASTASIOU $p = 2.62e^{-08}$; Fisher's exact test). The fact that

Figure 3. RASSF1C/SRC Destabilizes E-Cadherin at Cell-Cell Junctions

(A) Representative immunofluorescence images showing the localization of SRC and pY416-SRC at the sites of cell-cell junctions in control and RASSF1C-expressing H1299 cells. Graphs represent vector line measurements of eGFP-SRC (green) and pY416-SRC immunofluorescence (yellow) through a single cell (A) including bulk membrane (white arrow) or through two attached cells (A and B) including junctional membrane (red arrow).

(B) Graphs representing the localization of SRC and pY416-SRC at the sites of cell-cell junctions in control and RASSF1C-expressing cells in (A). Values are mean intensity of the immunofluorescent signal for the indicated antibody and representative of three independent experiments, 15 cells measurements per experiment.

(C) Phosphotyrosine immunoprecipitation from MCF7 cells expressing empty vector, FLAG-RASSF1A, FLAG-RASSF1C, or both. Bars indicate quantitation of the displayed images (ImageJ) and are representative of n = 3 experiments.

(D) Fluorescence intensity of MCF7 cells expressing GFP-E-cadherin or GFP-E-cadherin mutant (Y753F, Y754F, and Y755F) with either Ds-Red vector or Ds-Red-RASSF1C, quantified in graph (right). Quantification of GFP intensity was done on average of ten cells, three independent experiments.

(E) Immunofluorescence detection of YAP1 in MCF7 cells transfected with GFP-E-cadherin or GFP-E-cadherin mutant and either Ds-Red or DsRed-RASSF1C. Representative distribution of DAPI (blue) and YAP1 (black) across equatorial cell vector determined by immunofluorescence is shown (right).

All scale bars represent 20 μm.

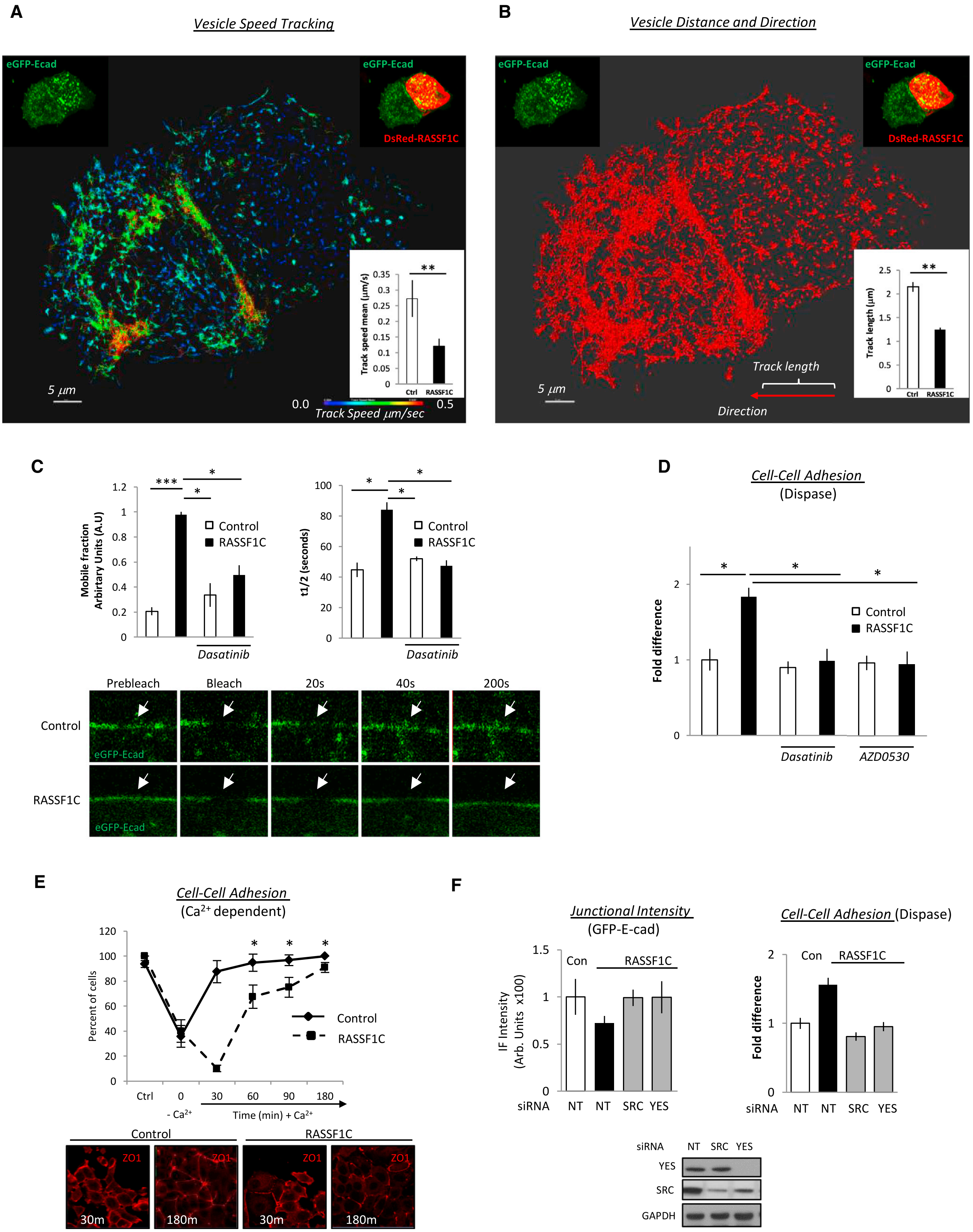

(legend on next page)

the entire data set is from invasive breast cancers negates direct mSigDB analysis in different groups; however, we could control for the increased invasive transcripts in the pS127-YAP1^{low} group as neither the YAP-TAZ or an unrelated signature displayed variation (Figures 7A and S7A; Table S1). We interpret these data in the breast (II) cohort to imply that the pS127-YAP1^{low} group has a YAP/TAZ signature but only an invasive signature when *RASSF1A* methylation is 100% penetrant (Table S2). Interestingly, this group showed significantly lower levels of the CSK substrate site pY527-SRC and elevated levels of both *BIRC5* and *MYC* (Figure 7B). Therefore, as RASSF1C is associated with SRC activation and transcription of these genes, we decided to investigate whether RASSF1C expression could promote tumorigenesis in vitro and in vivo. MDA-MB-231 cells, stably expressing either a control plasmid or RASSF1C, readily formed mammospheres in Matrigel. However, RASSF1C-associated mammospheres were significantly larger and displayed a more-aggressive phenotype that was SRC, YES, β-catenin, and YAP dependent (Figures 7C, 7D, and S7B–S7D). In line with these observations, RASSF1A methylation has recently been associated with brain metastasis [45]. To directly address tumorigenesis and invasive spread, we adopted a brain-seeding model where spread of human cells could be readily traced. MDA-MB-231 cells stably expressing RASSF1C, injected into the left striatum of SCID mice, formed significantly larger, more-aggressive tumors than either naive or empty vector controls (Figure 7E). The results collectively indicate the role of RASSF1C in invasiveness both in vitro and in vivo. The corroboration of RASSF1C activity with loss of the RASSSF1A transcript also supports increasing importance of *RASSF1A* promoter methylation with invasion, metastasis, and adverse outcome in multiple human tumors.

DISCUSSION

SRC and its associated family members play multiple roles in normal cell homeostasis controlling: cell proliferation and survival; cytoskeleton organization; cell shape; cell-cell and cell-ECM contacts; and motility. Deregulation of these activities promotes tumorigenesis, cancer cell invasion, and metastasis [46]. Evidence exists for elevation of SRC activity in tumors as a result of growth factor or cytokine signaling, but a clear somatic event is lacking [47]. In addition to mutational events and copy-number variations that drive tumors, epigenetic alterations are known to be a major contributing factor to disease progression and prognosis. We have identified that, similar to dRASSF8 binding to dCSK in *Drosophila* [30], RASSF1A associates with CSK and serves to keep SRC repressed. Inactivation of RASSF1A expression is the most widely observed epigenetic event across all sporadic human malignancies and has been confirmed to be a deleterious prognostic factor in meta-analyses of breast, bladder, lung, colorectal, prostate, esophageal, and ovarian cancers [10]. Therefore, the absence of CSK scaffolding to SRC, identified here, is likely to be a contributing factor in association of *RASSF1A* methylation with disease progression. In colorectal cancer, methylation of *RASSF1A* is not only associated with tumor dissemination [10], but it is found to be dramatically elevated in liver metastasis compared to primary tissue [48]. The fact that RASSF1A cooperates with APC loss to promote intestinal tumors [49] suggests that SRC activation may contribute to colorectal tumorigenesis by promoting the tyrosine phosphorylations of both β-catenin and YAP1 that are required for nuclear localization and transcriptional activation [16, 29, 39]. These data therefore provide an explanation for the prognostic value of *RASSF1A* methylation, while also providing a biomarker rational for treating RASSF1A-negative tumors with SRC inhibitors.

EMT is characterized by loss of cell-cell contacts through inactivation of E-cadherin and gain of mesenchymal markers. We find that RASSF1C expression in epithelial cells replicates a partial-EMT phenotype [18] where E-cadherin is expressed, together with the mesenchymal marker vimentin (data not shown), but prevented from forming stable contacts. Further, we observed that RASSF1C expression allows β-catenin and YAP1, normally sequestered at the membrane, to translocate to the nucleus. In addition to SRC-mediated destruction of cell-cell contacts in epithelial cells, we find that mesenchymal MDA-MB-231 cells also appear to require YES-mediated phosphorylation of YAP for nuclear targeting, as has been implicated previously for RUNX2 complexes [39] and in cancer-associated fibroblasts (CAFs) in response to mechanical stress [50]. Interestingly, the association of RASSF1A with filamin A and Arp3 (Figure 2A) suggests that actin dynamics and the mechanical stress response may by sensed by RASSF1A and contribute to SRC and YAP1 activation. RASSF1C also promotes nuclear accumulation of YAP1/β-catenin and upregulation of target genes that promote tumorigenesis, including BCL2L1 and

Figure 4. RASSF1C Expression Leads to Disruption of E-Cadherin Trafficking

(A and B) Representative images of the tracking of all the vesicles in control and DsRed-RASSF1C-expressing MCF7 cells, transfected with eGFP-E-cadherin (Imaris). Bar graphs (bottom right) show the analysis of the mean speed heatmap (A) or distance (B) of the vesicles in control and RASSF1C cells. For each analysis, five cells per experiment were used and an average of 700 vesicles were tracked (bars). The results are from three independent experiments including Movie S1. Inserts (top) display representative still immunofluorescence images showing the accumulation of E-cadherin and the expression of DsRed-RASSF1C.

(C) FRAP analysis of RASSF1C-expressing MCF7 cells. (Left) Mobile fraction of the return of GFP-E-cadherin after photobleaching is shown. (Right) Halftime of the return of GFP-E-cadherin at the sites of cell-cell junctions after bleaching in MCF7 cells is shown. (Bottom) Representative still images of Movies S2 and S3 displaying junctional GFP-E-cadherin in MCF7 cells, expressing DS-Red or Ds-Red-RASSF1C, captured prebleach and following bleach. Arrows, bleached area. For each of the three independent experiments, FRAP analysis was done on ten cells.

(D) Quantification of a dispase assay in MCF7 cells expressing Ds-Red or Ds-Red-RASSF1C in the presence or absence of dasatinib treatment (50 nM; 18 hr) or AZD0530 (2.5 μM; 18 hr) showing number of single cells in suspension.

(E) Quantification of total cell-cell contacts formed in calcium switch assay in MCF7 cells expressing Zs-green empty vector or Zs-green-RASSF1C. (Bottom) Representative images for ZO-1 at the sites of cell-cell junctions are shown.

(F) Quantification of a dispase assay (right) and the junctional intensity levels (left) of MCF7 cells transfected with siNT, siSRC, or siYES. (Bottom) Immunoblot indicating the level of siRNA knockdown is shown.

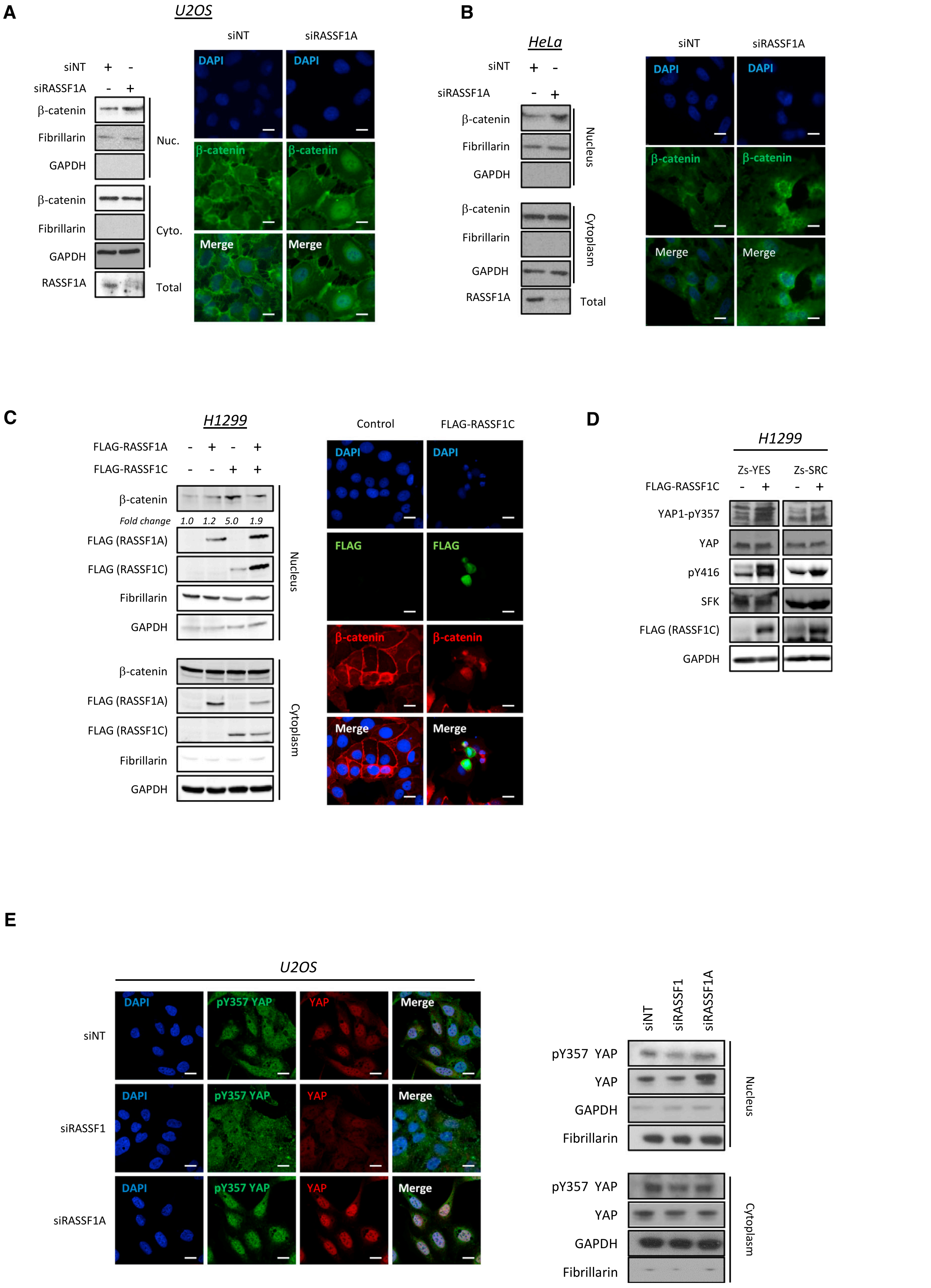

(legend on next page)

BIRC5. YAP1/β-catenin promote cancer cell proliferation and tumorigenesis via TBX5 [16] but also promote overgrowth of the heart through binding to TEAD [24]. Our data suggest that, in breast cancer cells, YAP1/β-catenin complex with TBX3, which phenocopies TBX5 at *BCL2L1* and *BIRC5* but additionally promotes *cMYC* expression, potentially by combining known TEAD- and TBX3-binding elements [28, 42].

We also confirm that RASSF1A activation of the hippo pathway maintains phosphorylation of YAP1, specifically pS127-YAP1 (Figures 1B and S1A), preventing association with TEAD [6, 15]. Upon loss of the pS127, YAP1 is permissive for activation but requires additional modifications for nuclear localization and transcriptional transactivation [15, 16, 29, 39], which we find are dependent on loss of the RASSF1A transcript and expression of RASSF1C (Figure 7F). Moreover, YAP-dependent tumorigenesis, mammosphere formation, and growth in soft agar have been attributed to BIRC5 expression [16, 42], which together with MYC-mediated invasion [44] is in line with the formation of larger, more-aggressive mammospheres in cells expressing RASSF1C and larger tumors in vivo. These results also explain the emerging association of both YAP1 and TBX3 with invasive cancers and motility during development [51, 52].

These phenotypes are supported by investigation of the clinical data sets that now include CGI methylation and protein phosphorylation, corroborating links of *RASSF1A* methylation with invasive spread and explaining prognostic association of this epigenetic event. Given the differential regulation of SFK complexes and YAP activity by RASSF1A and RASSF1C isoforms, the balance of expression of these two RASSF1 isoforms may provide an elegant mechanism for fine-tuning SFK signaling and YAP1 transcriptional activity during development and in emerging epithelial cancers. This isoform switch of RASSF1A may govern further SFK-regulatory events by the RASSF family, such as RASSF1C homolog Rapl-mediated regulation of SRC activation in innate immunity [53]. Moreover, is likely to be a common mechanism that adds complexity to the functionality of genetic information encoded by a single gene.

EXPERIMENTAL PROCEDURES

Proximity Ligation Assay

Proximity ligation assay was performed as described using the Duolink Starter Kit (Sigma). H1299 cells were transfected and treated as per instructions before overnight incubation with primary antibodies (SRC, SRC pY416, and SRC pY527 [Cell Signaling]; ZsGreen [Clontech]; RASSF1A [Epitomics]; RASSF1C [Abcam]; FLAG tag [Sigma]; and HA tag [Millipore]) and secondary antibodies for 1 hr. Hybridization was performed for 30 min in a humidified chamber, ligation reactions for 30 min, amplification for 100 min, and DAPI was used for cell detection. The dot-like structures were imaged using Zeiss LSM780 microscope using a 63× objective. For each cell, five z stack images were taken and analyzed with BlobFinder V3.2 (Uppsala University) [54]. The average values, SEM, and significance were calculated using Prism 6.0 (Graphpad) software.

Real-Time Molecular Visualization

MCF7 cells (2×10^5 cells/condition) on glass bottom plates (Ibidi) were transfected with GFP-E-cadherin and DsRed or DsRed-RASSF1C plasmids. Cells were grown in complete media until imaging, when media was changed to DMEM/F-12 media without phenol red and supplemented with 10% (v/v) FBS and 100 U/ml Pen/strep. Cells were imaged using Zeiss LSM780 confocal microscope using a 63× objective, for the time-lapse one image per second for a total of 200 s. Tracking of internalized E-cadherin was achieved using Imaris 7.71 software (Bitplane; ANDOR). Threshold was determined for each video and varied between 0.4 and 0.6 μm in size. The average distance traveled was set at a threshold of 1 μm. A Quality filter was applied and the threshold set at 15. The data were extracted using the Vantage feature and average values, SEM, and significance were calculated using Prism 6.0 (Graphpad) software. On average, five cells and 700 vesicles each were counted per experiment, and the data shown represent three independent experiments.

Fluorescence Recovery after Photobleaching

MCF7 cells (2×10^5 cells/condition) were plated on 35-mm glass bottom plates (Ibidi). Cells were grown in complete media until imaging, when media was changed to DMEM/F-12 media without phenol red and supplemented with 10% (v/v) FBS and 100 U/ml pen/strep. Cells were imaged using Zeiss LSM780 confocal microscope using a 63× (NA 1.4) objective. Each bleach was done at full laser power, and two pulses were used in order to achieve 60% bleach per region of interest. Images were taken every 2 s for 5 min after the bleach. Data were analyzed using easyFRAP software [55]. The immobile and mobile fractions were calculated using double exponential formula (intensity $[I] = IE - I1 \times e(-t/T1) - I2 \times e(-t/T2)$).

Animal Experiments

Mice were anesthetized and skull burr-hole drilled. Animals were each focally injected with 5×10^3 MDA-MB-231 tumor cells expressing empty vector (pCDNA3) or MYC-RASSF1C in 0.5 μl PBS in the left striatum using a 75-mm-tipped glass microcapillary (Clark Electromedical Instruments). At day 21, all animals were transcardially perfusion fixed under terminal anesthesia (n = 4 per group) and brains were post-fixed, cryoprotected, embedded, and frozen in isopentane at −40°C. To assess areas of tumor colonization, photomicrographs of each brain section were obtained using ScanScope CS slide scanner (Aperio) and analyzed using ImageScope (Aperio). For immunofluorescence, sections were streptavidin and biotin blocked, incubated with anti-CD34 primary antibody (Abcam; brain vessels) or anti-vimentin antibody (VectorLabs; tumor cells), washed, and incubated with a streptavidin-Cy3 fluorophore or AMCA-conjugated secondary antibody (Invitrogen; 1:100) for 30 min. Expanded animal experimental procedures are outlined in Supplemental Experimental Procedures.

Figure 5. RASSF1C Leads to β-Catenin Nuclear Localization and pY357

(A) Nuclear/cytoplasmic fractionation of U2OS cells transfected with siNT or siRASSF1A (left). Representative images of U2OS cells transfected with siNT or siRASSF1A are shown (right).

(B) Nuclear/cytoplasmic fractionation (left) and immunofluorescence detection of β-catenin (right) of HeLa cells transfected with siNT or siRASSF1A.

(C) Nuclear/cytoplasmic fractionation of H1299 cells transiently transfected with empty vector, FLAG-RASSF1A, FLAG-RASSF1C, or both FLAG-RASSF1A and FLAG-RASSF1C to show β-catenin localization. (Right) Representative images show β-catenin localization in H1299 transfected with empty vector or FLAG-RASSF1C.

(D) H1299 cells transfected with Zs-Green-SRC or Zs-Green-YES and either empty vector or FLAG-RASSF1C and blotted for pY416-SRC, YAP, and the SFK site, pY357-YAP1, as indicated.

(E) Immunofluorescence detection of YAP1 and pY357-YAP1 in U2OS cells transfected with siNT, siRASSF1, or siRASSF1A (left) and immunoblot showing the nuclear/cytoplasmic distribution of YAP1 (right).

All scale bars represent 20 μm.

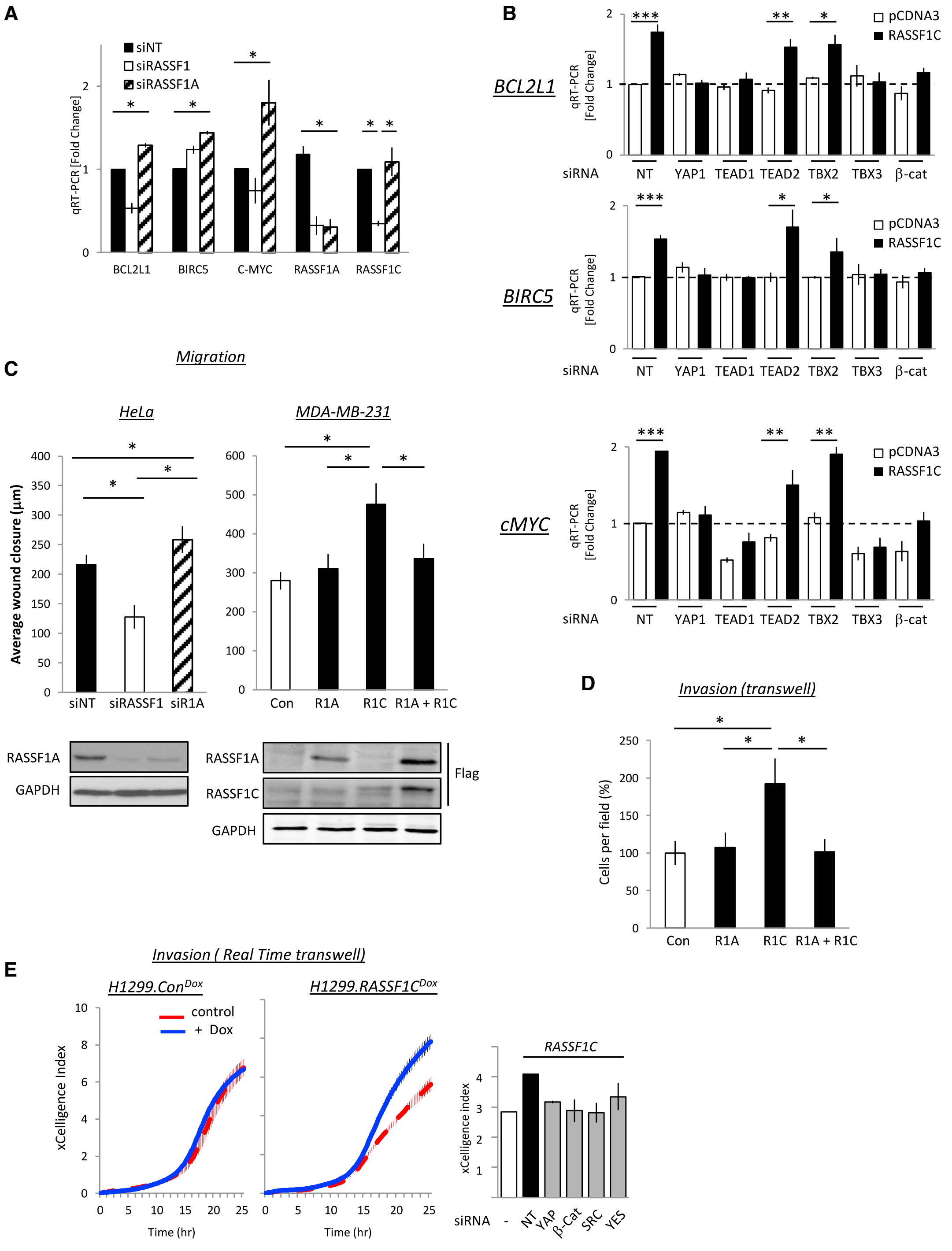

Figure 6. RASSF1C Promotes YAP1-Dependent Transcription and Cell Motility

(A) Quantification of qRT-PCR for the β-catenin-YAP target genes *BCL2L1*, *BIRC5*, and *cMYC* as well as RASSF1A and RASSF1C expression in U2OS cells transfected with siNT, siRASSF1, or siRASSF1A.

(B) Quantification of qRT-PCR for the β-catenin-YAP target genes *BCL2L1*, *BIRC5*, and *cMYC* in MCF7 cells transfected with empty vector or FLAG-RASSF1C and indicated siRNAs.

(C) Scratch wound motility assay of HeLa cells transfected with non-targeting siNT, siRASSF1, and siRASSF1A (left). Wound healing motility assay of MDA-MB-231 cells transfected with empty vector, FLAG-RASSF1A, FLAG-RASSF1C, or both FLAG-RASSF1A and FLAG-RASSF1C is shown (right).

(D) Quantification of a Transwell assay with MDA-MB-231 cells expressing empty vector, FLAG-RASSF1A, FLAG-RASSF1C, or both.

(E) Migration assay in real time using ExCELLigence analyzer with H1299.R1C^{Dox} TET-ON FLAG-RASSF1C inducible cells (right) or controls H1299.Con^{Dox} TET-ON empty-vector-inducible cells (left) in the presence or absence of 1 μg/μl doxycycline. (Right) Migration assay in real time using ExCELLigence analyzer on MCF7 cells transfected with empty vector or FLAG-RASSF1C with siNT, siYAP1, siβ-catenin, siSRC, or siYES is shown.

A

Patients	Low methyl *RASSF1*	High methyl *RASSF1*	Low p127 vs Low YAP GSEA significance (Fischer's exact) *Summary of Supplementary Table 2.
Glioma	79%	21%	Invasive breast (POOLA)<br>Metastasis (BIDUS)<br>EMT (ANASTASSIOU)<br>YAP_TAZ
Bladder	78%	22%	Invasive breast (POOLA)<br>Metastasis (BIDUS)<br>EMT (ANASTASSIOU)<br>YAP_TAZ
Breast (I)	84%	16%	Invasive breast (POOLA)<br>Metastasis (BIDUS)<br>EMT (ANASTASSIOU)<br>YAP_TAZ
Breast (II)	0	100%	**Invasive breast (POOLA)**<br>**Metastasis (BIDUS)**<br>**EMT (ANASTASSIOU)**<br>**YAP_TAZ**

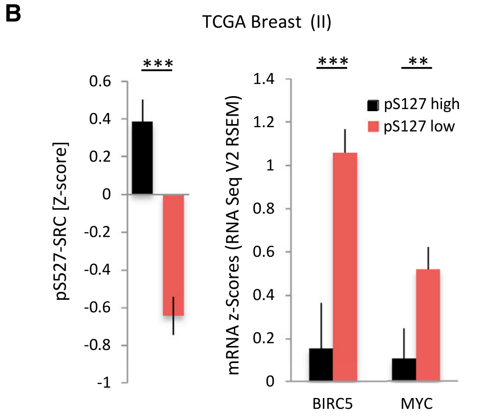

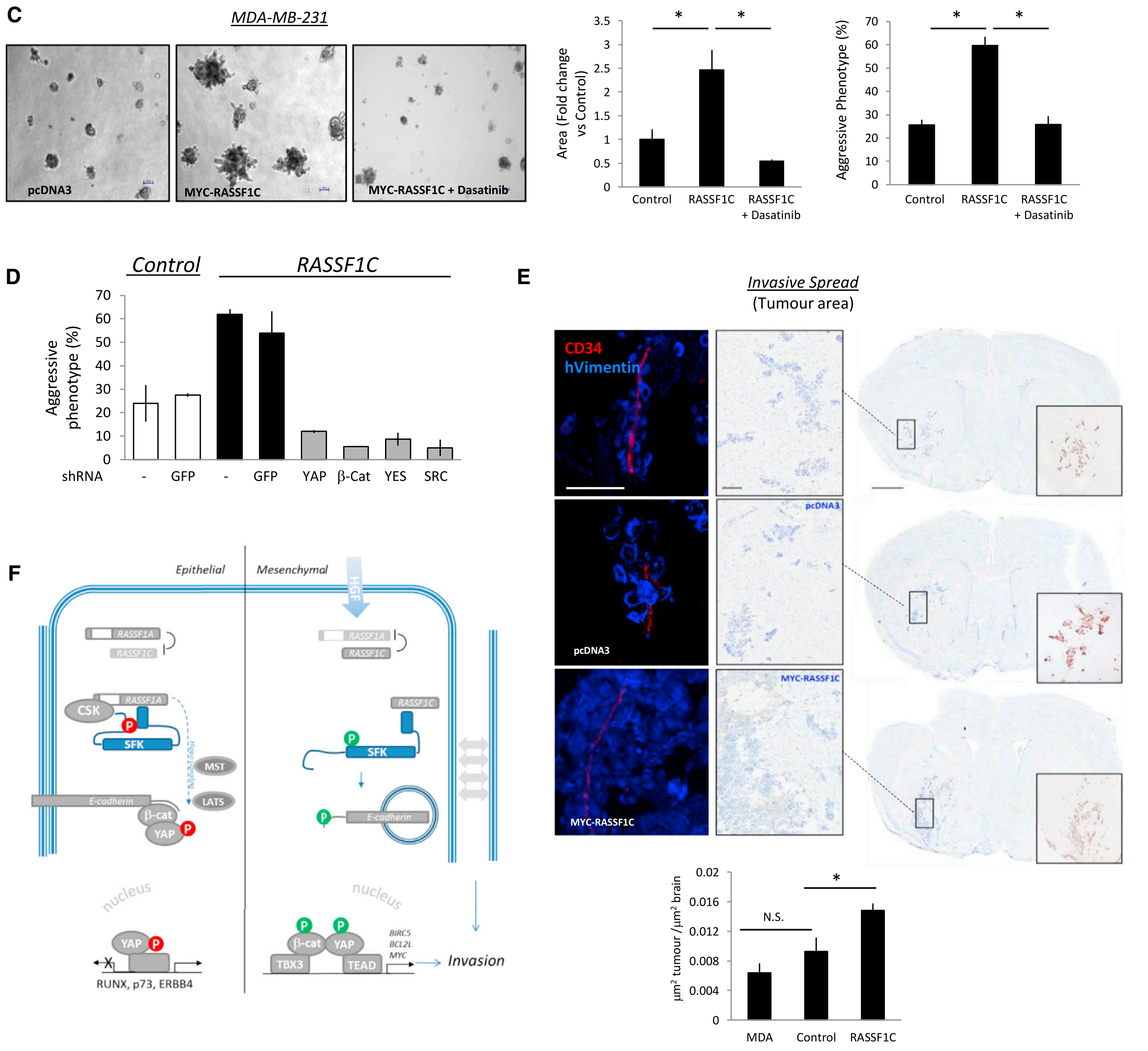

(legend on next page)

All animal experiments were approved by the University of Oxford local animal ethical committee and were performed according to terms of a license granted by the UK Home Office, adhering to the Animals (Scientific Procedures) Act 1986.

The Cancer Genome Project Analysis

The data were downloaded from cBioPortal for Cancer Genomics [56, 57] and analyzed with SPSS 21.0 and R version 3.0.1 software. For each clinical data set, cases with missing (NA) methylation, protein, or gene expression values were excluded where appropriate. The Shapiro-Wilk test was used to assess distribution of data sets, null hypothesis of normal distribution was rejected at $p < 0.05$ level, and the non-parametric Spearman-Rho test was used for correlation. The non-parametric Kruskal-Wallis or Jonckheere-Terpstra was used to compare the differences of either protein or gene expression levels between the appropriate groups analyzed. The Molecular Signatures Database (MSigDB v4.0) [58] was used to select gene sets, and POOLA_ INVASIVE_ BREAST_ CANCER_ UP [59], BIDUS_ METASTASIS_ UP [60], ANASTASSIOU_ CANCER_ MESENCHYMAL_ TRANSITION_ SIGNATURE [61], CORDENONSI_ YAP_ CONSERVED_ SIGNATURE [62], REACTOME_ YAP1_ AND_ WWTR1_ TAZ_ STIMULATED_ GENE_ EXPRESSION, and BLALOCK_ ALZHEIMERS_ DISEASE_ INCIPIENT_ UP [63] were selected as either test or control gene signature sets. The non-parametric Mann-Whitney test was used to compare variation in gene expression between the groups analyzed and the Fisher's exact test used to compare differences in frequency distributions.

Statistics

For all in vitro experiments, statistical analysis was carried out using a Student's t test. Tumor areas were compared by ANOVA followed by post hoc Newman-Keuls t tests. All data are expressed as mean ± SEM.

SUPPLEMENTAL INFORMATION

Supplemental Information includes seven figures, two tables, Supplemental Experimental Procedures, and five movies and can be found with this article online at http://dx.doi.org/10.1016/j.cub.2015.09.072.

AUTHOR CONTRIBUTIONS

N.V. and S.S. helped design and performed the majority of experiments. M.S.S. and N.S. contributed the in vivo experiments. A.M.G. performed the bioinformatics analyses with advice from F.B. L.B., A.P., and K.S.Y. contributed to nuclear localization and hippo pathway experiments. D.P. and S.S. performed the spheroid analysis. C.R.G. assisted with TBX reagents and advice. P.T. advised S.S. on imaging analysis of Src and E-cadherin. E.O. is responsible for the concept and designed and wrote the manuscript with S.S. and N.V.

ACKNOWLEDGMENTS

We would like to thank A. Harris for advice and G. Brown for assistance with Imaris imaging. This work was supported by Cancer Research UK A19277, Cancer Research UK studentships (S.S. and N.V.), a Medical Research Council Studentship (A.P.), and an Oxford Cancer Research Centre development fund award to S.S. The authors apologize for any references omitted due to space constraints.

Received: February 26, 2015
Revised: July 23, 2015
Accepted: September 25, 2015
Published: November 5, 2015

REFERENCES

1. Moroishi, T., Hansen, C.G., and Guan, K.L. (2015). The emerging roles of YAP and TAZ in cancer. Nat. Rev. Cancer *15*, 73–79.
2. Couzens, A.L., Knight, J.D., Kean, M.J., Teo, G., Weiss, A., Dunham, W.H., Lin, Z.Y., Bagshaw, R.D., Sicheri, F., Pawson, T., et al. (2013). Protein interaction network of the mammalian Hippo pathway reveals mechanisms of kinase-phosphatase interactions. Sci. Signal. *6*, rs15.
3. Harvey, K.F., Zhang, X., and Thomas, D.M. (2013). The Hippo pathway and human cancer. Nat. Rev. Cancer *13*, 246–257.
4. Yee, K.S., Grochola, L., Hamilton, G., Grawenda, A., Bond, E.E., Taubert, H., Wurl, P., Bond, G.L., and O'Neill, E. (2012). A RASSF1A polymorphism restricts p53/p73 activation and associates with poor survival and accelerated age of onset of soft tissue sarcoma. Cancer Res. *72*, 2206–2217.
5. Pefani, D.E., Latusek, R., Pires, I., Grawenda, A.M., Yee, K.S., Hamilton, G., van der Weyden, L., Esashi, F., Hammond, E.M., and O'Neill, E. (2014). RASSF1A-LATS1 signalling stabilizes replication forks by restricting CDK2-mediated phosphorylation of BRCA2. Nat. Cell Biol. *16*, 962–971, 1–8.
6. van der Weyden, L., Papaspyropoulos, A., Poulogiannis, G., Rust, A.G., Rashid, M., Adams, D.J., Arends, M.J., and O'Neill, E. (2012). Loss of RASSF1A synergizes with deregulated RUNX2 signaling in tumorigenesis. Cancer Res. *72*, 3817–3827.
7. Chamberlain, C.E., Scheel, D.W., McGlynn, K., Kim, H., Miyatsuka, T., Wang, J., Nguyen, V., Zhao, S., Mavropoulos, A., Abraham, A.G., et al.

Figure 7. RASSF1C Promotes Invasive Spread and Tumorigenesis In Vivo

(A) Cohorts of glioma, bladder, and breast (I, Koboldt; II, invasive carcinoma) from The Cancer Genome Atlas (TCGA) with percent of patients with RASSF1A methylation as indicated. Relative significance of gene changes from MSigDB signatures in active YAP1 is shown (pS127-YAP1^{low} versus YAP1), red ($p < 0.5$); bold ($p < 0.000001$); both groups have identical expression of genes for YAP-TAZ signatures (green).

(B) The pS127-YAP1^{low} population in the TCGA Breast (II) displays lower pY527-SRC and increased BIRC5 and MYC mRNA (red bars) compared to the pS127-YAP1high population (black bars).

(C) Mammospheres from MDA-MB-231 cells stably expressing empty vector, MYC-RASSF1C, or MYC-RASSF1C treated on days 3, 6, and 9 with SFK inhibitor (10 nM dasatinib) grown in Matrigel. Images were taken on day 10. Graphs indicate size of mammospheres (middle) and mammospheres with aggressive phenotypes (right). All scale bars represent 50 μm.

(D) Quantification of mammosphere growth experiment on Matrigel, using MDA-MB-231 cells stably expressing empty vector or MYC-RASSF1C and transfected with shGFP, shYAP1, shβ-catenin, shYES, or shSRC.

(E) Immunofluorescence photomicrographs showing tumor growth patterns in each of the experimental groups at day 21 after intracerebral tumor cell injection (left). Brain vessels stained with CD34 (red: Cye3 fluorophore) and tumor cells stained with human vimentin (blue: AMCA fluorophore) are shown. The scale bar represents 50 μm. Immunohistochemical photomicrographs of the tumor core for each tumor cell line counterstained with cresyl violet are shown (right). The scale bar represents 1 mm. Higher-resolution images of boxed region are shown on the left-hand side (scale bars 100 μm), and insets show contiguous sections in which tumor cells were stained with anti-vimentin antibody. (Bottom) Graph shows area of tumor colonization in each of the experimental groups 21 days after intracerebral injection. Error bars represent SEM.

(F) Model. The hippo pathway both prevents YAP1-TEAD association and promotes cytoplasmic YAP1. RASSF1A sustains CSK inhibitory phosphorylation of SRC family kinases (SFKs) in response to HGF (Figure S2C) and promotes hippo-mediated pS127-YAP1 and tumor suppressive transcription. EMT signals that disrupt RASSF1A or epigenetic inactivation (*meRASSF1*) make YAP1 permissive for TEAD transcription and allow RASSF1C to activate SFKs. Internalization of E-cadherin and phosphorylation of β-catenin and Y357-YAP1 then promotes nuclear localization and TBX/TEAD-mediated transcription of invasive genes invasive transcription.

(2014). Menin determines K-RAS proliferative outputs in endocrine cells. J. Clin. Invest. *124*, 4093–4101.

8. Kapoor, A., Yao, W., Ying, H., Hua, S., Liewen, A., Wang, Q., Zhong, Y., Wu, C.J., Sadanandam, A., Hu, B., et al. (2014). Yap1 activation enables bypass of oncogenic Kras addiction in pancreatic cancer. Cell *158*, 185–197.
9. Zhang, H., von Gise, A., Liu, Q., Hu, T., Tian, X., He, L., Pu, W., Huang, X., He, L., Cai, C.L., et al. (2014). Yap1 is required for endothelial to mesenchymal transition of the atrioventricular cushion. J. Biol. Chem. *289*, 18681–18692.
10. Grawenda, A.M., and O'Neill, E. (2015). Clinical utility of RASSF1A methylation in human malignancies. Br. J. Cancer *113*, 372–381.
11. Hamilton, G., Yee, K.S., Scrace, S., and O'Neill, E. (2009). ATM regulates a RASSF1A-dependent DNA damage response. Curr. Biol. *19*, 2020–2025.
12. Matallanas, D., Romano, D., Yee, K., Meissl, K., Kucerova, L., Piazzolla, D., Baccarini, M., Vass, J.K., Kolch, W., and O'neill, E. (2007). RASSF1A elicits apoptosis through an MST2 pathway directing proapoptotic transcription by the p73 tumor suppressor protein. Mol. Cell *27*, 962–975.
13. Guo, C., Tommasi, S., Liu, L., Yee, J.K., Dammann, R., and Pfeifer, G.P. (2007). RASSF1A is part of a complex similar to the Drosophila Hippo/Salvador/Lats tumor-suppressor network. Curr. Biol. *17*, 700–705.
14. Praskova, M., Xia, F., and Avruch, J. (2008). MOBKL1A/MOBKL1B phosphorylation by MST1 and MST2 inhibits cell proliferation. Curr. Biol. *18*, 311–321.
15. Ahn, E.Y., Kim, J.S., Kim, G.J., and Park, Y.N. (2013). RASSF1A-mediated regulation of AREG via the Hippo pathway in hepatocellular carcinoma. Mol. Cancer Res. *11*, 748–758.
16. Rosenbluh, J., Nijhawan, D., Cox, A.G., Li, X., Neal, J.T., Schafer, E.J., Zack, T.I., Wang, X., Tsherniak, A., Schinzel, A.C., et al. (2012). β-Catenin-driven cancers require a YAP1 transcriptional complex for survival and tumorigenesis. Cell *151*, 1457–1473.
17. Shao, D.D., Xue, W., Krall, E.B., Bhutkar, A., Piccioni, F., Wang, X., Schinzel, A.C., Sood, S., Rosenbluh, J., Kim, J.W., et al. (2014). KRAS and YAP1 converge to regulate EMT and tumor survival. Cell *158*, 171–184.
18. Friedl, P., and Alexander, S. (2011). Cancer invasion and the microenvironment: plasticity and reciprocity. Cell *147*, 992–1009.
19. Behrens, J., Vakaet, L., Friis, R., Winterhager, E., Van Roy, F., Mareel, M.M., and Birchmeier, W. (1993). Loss of epithelial differentiation and gain of invasiveness correlates with tyrosine phosphorylation of the E-cadherin/beta-catenin complex in cells transformed with a temperature-sensitive v-SRC gene. J. Cell Biol. *120*, 757–766.
20. Lilien, J., and Balsamo, J. (2005). The regulation of cadherin-mediated adhesion by tyrosine phosphorylation/dephosphorylation of beta-catenin. Curr. Opin. Cell Biol. *17*, 459–465.
21. Yap, A.S., Niessen, C.M., and Gumbiner, B.M. (1998). The juxtamembrane region of the cadherin cytoplasmic tail supports lateral clustering, adhesive strengthening, and interaction with p120ctn. J. Cell Biol. *141*, 779–789.
22. Roura, S., Miravet, S., Piedra, J., García de Herreros, A., and Duñach, M. (1999). Regulation of E-cadherin/Catenin association by tyrosine phosphorylation. J. Biol. Chem. *274*, 36734–36740.
23. Coluccia, A.M., Benati, D., Dekhil, H., De Filippo, A., Lan, C., and Gambacorti-Passerini, C. (2006). SKI-606 decreases growth and motility of colorectal cancer cells by preventing pp60(c-Src)-dependent tyrosine phosphorylation of beta-catenin and its nuclear signaling. Cancer Res. *66*, 2279–2286.
24. Heallen, T., Zhang, M., Wang, J., Bonilla-Claudio, M., Klysik, E., Johnson, R.L., and Martin, J.F. (2011). Hippo pathway inhibits Wnt signaling to restrain cardiomyocyte proliferation and heart size. Science *332*, 458–461.
25. Hovestadt, V., Jones, D.T., Picelli, S., Wang, W., Kool, M., Northcott, P.A., Sultan, M., Stachurski, K., Ryzhova, M., Warnatz, H.J., et al. (2014). Decoding the regulatory landscape of medulloblastoma using DNA methylation sequencing. Nature *510*, 537–541.
26. Reeves, M.E., Baldwin, S.W., Baldwin, M.L., Chen, S.T., Moretz, J.M., Aragon, R.J., Li, X., Strong, D.D., Mohan, S., and Amaar, Y.G. (2010). Ras-association domain family 1C protein promotes breast cancer cell migration and attenuates apoptosis. BMC Cancer *10*, 562.
27. Malpeli, G., Amato, E., Dandrea, M., Fumagalli, C., Debattisti, V., Boninsegna, L., Pelosi, G., Falconi, M., and Scarpa, A. (2011). Methylation-associated down-regulation of RASSF1A and up-regulation of RASSF1C in pancreatic endocrine tumors. BMC Cancer *11*, 351.
28. Han, J., Yuan, P., Yang, H., Zhang, J., Soh, B.S., Li, P., Lim, S.L., Cao, S., Tay, J., Orlov, Y.L., et al. (2010). Tbx3 improves the germ-line competency of induced pluripotent stem cells. Nature *463*, 1096–1100.
29. Taniguchi, K., Wu, L.W., Grivennikov, S.I., de Jong, P.R., Lian, I., Yu, F.X., Wang, K., Ho, S.B., Boland, B.S., Chang, J.T., et al. (2015). A gp130-Src-YAP module links inflammation to epithelial regeneration. Nature *519*, 57–62.
30. Langton, P.F., Colombani, J., Chan, E.H., Wepf, A., Gstaiger, M., and Tapon, N. (2009). The dASPP-dRASSF8 complex regulates cell-cell adhesion during Drosophila retinal morphogenesis. Curr. Biol. *19*, 1969–1978.
31. Sandilands, E., and Frame, M.C. (2008). Endosomal trafficking of Src tyrosine kinase. Trends Cell Biol. *18*, 322–329.
32. Mukherjee, M., Chow, S.Y., Yusoff, P., Seetharaman, J., Ng, C., Sinniah, S., Koh, X.W., Asgar, N.F., Li, D., Yim, D., et al. (2012). Structure of a novel phosphotyrosine-binding domain in Hakai that targets E-cadherin. EMBO J. *31*, 1308–1319.
33. Schlegelmilch, K., Mohseni, M., Kirak, O., Pruszak, J., Rodriguez, J.R., Zhou, D., Kreger, B.T., Vasioukhin, V., Avruch, J., Brummelkamp, T.R., and Camargo, F.D. (2011). Yap1 acts downstream of α-catenin to control epidermal proliferation. Cell *144*, 782–795.
34. Silvis, M.R., Kreger, B.T., Lien, W.H., Klezovitch, O., Rudakova, G.M., Camargo, F.D., Lantz, D.M., Seykora, J.T., and Vasioukhin, V. (2011). α-catenin is a tumor suppressor that controls cell accumulation by regulating the localization and activity of the transcriptional coactivator Yap1. Sci. Signal. *4*, ra33.
35. Kim, N.G., Koh, E., Chen, X., and Gumbiner, B.M. (2011). E-cadherin mediates contact inhibition of proliferation through Hippo signaling-pathway components. Proc. Natl. Acad. Sci. USA *108*, 11930–11935.
36. Le, T.L., Yap, A.S., and Stow, J.L. (1999). Recycling of E-cadherin: a potential mechanism for regulating cadherin dynamics. J. Cell Biol. *146*, 219–232.
37. Canel, M., Serrels, A., Miller, D., Timpson, P., Serrels, B., Frame, M.C., and Brunton, V.G. (2010). Quantitative in vivo imaging of the effects of inhibiting integrin signaling via Src and FAK on cancer cell movement: effects on E-cadherin dynamics. Cancer Res. *70*, 9413–9422.
38. Estrabaud, E., Lassot, I., Blot, G., Le Rouzic, E., Tanchou, V., Quemeneur, E., Daviet, L., Margottin-Goguet, F., and Benarous, R. (2007). RASSF1C, an isoform of the tumor suppressor RASSF1A, promotes the accumulation of beta-catenin by interacting with betaTrCP. Cancer Res. *67*, 1054–1061.
39. Zaidi, S.K., Sullivan, A.J., Medina, R., Ito, Y., van Wijnen, A.J., Stein, J.L., Lian, J.B., and Stein, G.S. (2004). Tyrosine phosphorylation controls Runx2-mediated subnuclear targeting of YAP to repress transcription. EMBO J. *23*, 790–799.
40. Papaioannou, V.E. (2014). The T-box gene family: emerging roles in development, stem cells and cancer. Development *141*, 3819–3833.
41. He, T.C., Sparks, A.B., Rago, C., Hermeking, H., Zawel, L., da Costa, L.T., Morin, P.J., Vogelstein, B., and Kinzler, K.W. (1998). Identification of c-MYC as a target of the APC pathway. Science *281*, 1509–1512.
42. Dong, J., Feldmann, G., Huang, J., Wu, S., Zhang, N., Comerford, S.A., Gayyed, M.F., Anders, R.A., Maitra, A., and Pan, D. (2007). Elucidation of a universal size-control mechanism in Drosophila and mammals. Cell *130*, 1120–1133.
43. Gómez-Casares, M.T., García-Alegria, E., López-Jorge, C.E., Ferrándiz, N., Blanco, R., Alvarez, S., Vaqué, J.P., Bretones, G., Caraballo, J.M., Sánchez-Bailón, P., et al. (2013). MYC antagonizes the differentiation

induced by imatinib in chronic myeloid leukemia cells through downregulation of p27(KIP1.). Oncogene *32*, 2239–2246.

44. Wolfer, A., and Ramaswamy, S. (2011). MYC and metastasis. Cancer Res. *71*, 2034–2037.
45. Marzese, D.M., Scolyer, R.A., Roqué, M., Vargas-Roig, L.M., Huynh, J.L., Wilmott, J.S., Murali, R., Buckland, M.E., Barkhoudarian, G., Thompson, J.F., et al. (2014). DNA methylation and gene deletion analysis of brain metastases in melanoma patients identifies mutually exclusive molecular alterations. Neuro-oncol. *16*, 1499–1509.
46. Yeatman, T.J. (2004). A renaissance for SRC. Nat. Rev. Cancer *4*, 470–480.
47. Irby, R.B., and Yeatman, T.J. (2000). Role of Src expression and activation in human cancer. Oncogene *19*, 5636–5642.
48. Tommasi, S., Besaratinia, A., Wilczynski, S.P., and Pfeifer, G.P. (2011). Loss of Rassf1a enhances p53-mediated tumor predisposition and accelerates progression to aneuploidy. Oncogene *30*, 690–700.
49. van der Weyden, L., Arends, M.J., Dovey, O.M., Harrison, H.L., Lefebvre, G., Conte, N., Gergely, F.V., Bradley, A., and Adams, D.J. (2008). Loss of Rassf1a cooperates with Apc(Min) to accelerate intestinal tumourigenesis. Oncogene *27*, 4503–4508.
50. Calvo, F., Ege, N., Grande-Garcia, A., Hooper, S., Jenkins, R.P., Chaudhry, S.I., Harrington, K., Williamson, P., Moeendarbary, E., Charras, G., and Sahai, E. (2013). Mechanotransduction and YAP-dependent matrix remodelling is required for the generation and maintenance of cancer-associated fibroblasts. Nat. Cell Biol. *15*, 637–646.
51. Fukui, H., Terai, K., Nakajima, H., Chiba, A., Fukuhara, S., and Mochizuki, N. (2014). S1P-Yap1 signaling regulates endoderm formation required for cardiac precursor cell migration in zebrafish. Dev. Cell *31*, 128–136.
52. Shen, J., Lu, J., Sui, L., Wang, D., Yin, M., Hoffmann, I., Legler, A., and Pflugfelder, G.O. (2014). The orthologous Tbx transcription factors Omb and TBX2 induce epithelial cell migration and extrusion in vivo without involvement of matrix metalloproteinases. Oncotarget *5*, 11998–12015.
53. Han, C., Jin, J., Xu, S., Liu, H., Li, N., and Cao, X. (2010). Integrin CD11b negatively regulates TLR-triggered inflammatory responses by activating Syk and promoting degradation of MyD88 and TRIF via Cbl-b. Nat. Immunol. *11*, 734–742.
54. Allalou, A., and Wählby, C. (2009). BlobFinder, a tool for fluorescence microscopy image cytometry. Comput. Methods Programs Biomed. *94*, 58–65.
55. Rapsomaniki, M.A., Kotsantis, P., Symeonidou, I.E., Giakoumakis, N.N., Taraviras, S., and Lygerou, Z. (2012). easyFRAP: an interactive, easy-to-use tool for qualitative and quantitative analysis of FRAP data. Bioinformatics *28*, 1800–1801.
56. Cerami, E., Gao, J., Dogrusoz, U., Gross, B.E., Sumer, S.O., Aksoy, B.A., Jacobsen, A., Byrne, C.J., Heuer, M.L., Larsson, E., et al. (2012). The cBio cancer genomics portal: an open platform for exploring multidimensional cancer genomics data. Cancer Discov. *2*, 401–404.
57. Gao, J., Aksoy, B.A., Dogrusoz, U., Dresdner, G., Gross, B., Sumer, S.O., Sun, Y., Jacobsen, A., Sinha, R., Larsson, E., et al. (2013). Integrative analysis of complex cancer genomics and clinical profiles using the cBioPortal. Sci. Signal. *6*, pl1.
58. Subramanian, A., Tamayo, P., Mootha, V.K., Mukherjee, S., Ebert, B.L., Gillette, M.A., Paulovich, A., Pomeroy, S.L., Golub, T.R., Lander, E.S., and Mesirov, J.P. (2005). Gene set enrichment analysis: a knowledge-based approach for interpreting genome-wide expression profiles. Proc. Natl. Acad. Sci. USA *102*, 15545–15550.
59. Poola, I., DeWitty, R.L., Marshalleck, J.J., Bhatnagar, R., Abraham, J., and Leffall, L.D. (2005). Identification of MMP-1 as a putative breast cancer predictive marker by global gene expression analysis. Nat. Med. *11*, 481–483.
60. Bidus, M.A., Risinger, J.I., Chandramouli, G.V.R., Dainty, L.A., Litzi, T.J., Berchuck, A., Barrett, J.C., and Maxwell, G.L. (2006). Prediction of lymph node metastasis in patients with endometrioid endometrial cancer using expression microarray. Clin. Cancer Res. *12*, 83–88.
61. Anastassiou, D., Rumjantseva, V., Cheng, W., Huang, J., Canoll, P.D., Yamashiro, D.J., and Kandel, J.J. (2011). Human cancer cells express Slug-based epithelial-mesenchymal transition gene expression signature obtained in vivo. BMC Cancer *11*, 529.
62. Cordenonsi, M., Zanconato, F., Azzolin, L., Forcato, M., Rosato, A., Frasson, C., Inui, M., Montagner, M., Parenti, A.R., Poletti, A., et al. (2011). The Hippo transducer TAZ confers cancer stem cell-related traits on breast cancer cells. Cell *147*, 759–772.
63. Blalock, E.M., Geddes, J.W., Chen, K.C., Porter, N.M., Markesbery, W.R., and Landfield, P.W. (2004). Incipient Alzheimer's disease: microarray correlation analyses reveal major transcriptional and tumor suppressor responses. Proc. Natl. Acad. Sci. USA *101*, 2173–2178.

Current Biology

Supplemental Information

Alternate RASSF1 Transcripts Control SRC Activity, E-Cadherin Contacts, and YAP-Mediated Invasion

Nikola Vlahov, Simon Scrace, Manuel Sarmiento Soto, Anna M. Grawenda, Leanne Bradley, Daniela Pankova, Angelos Papaspyropoulos, Karen S. Yee, Francesca Buffa, Colin R. Goding, Paul Timpson, Nicola Sibson, and Eric O'Neill

Figure S1

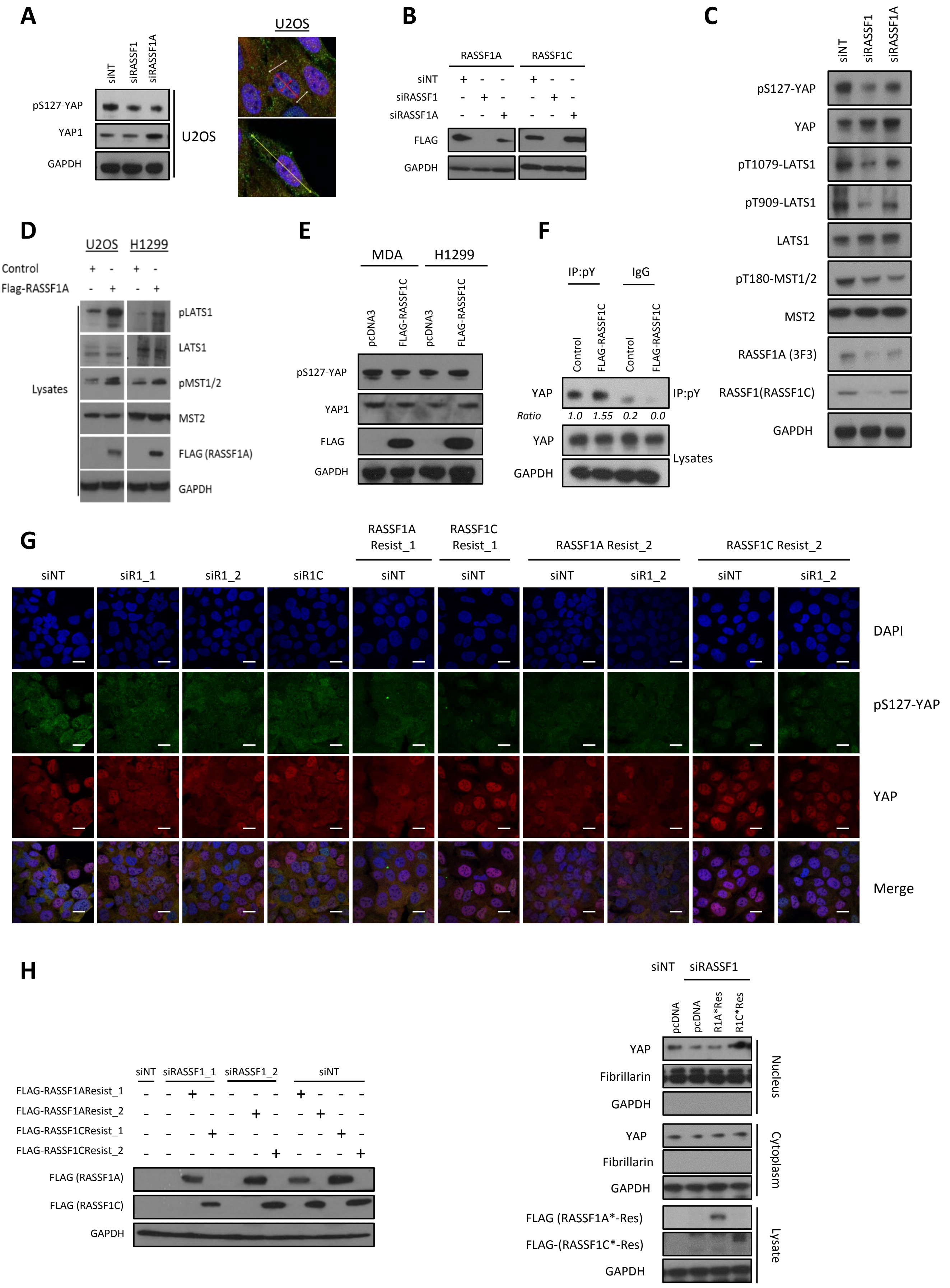
A
siNT
siRASSF1
siRASSF1A
pS127-YAP
YAP1
GAPDH
U2OS
U2OS
B
RASSF1A
RASSF1C
siNT
siRASSF1
siRASSF1A
FLAG
GAPDH
C
siNT
siRASSF1
siRASSF1A
pS127-YAP
YAP
pT1079-LATS1
pT909-LATS1
LATS1
pT180-MST1/2
MST2
RASSF1A (3F3)
RASSF1(RASSF1C)
GAPDH
D
U2OS
H1299
Control
Flag-RASSF1A
Lysates
pLATS1
LATS1
pMST1/2
MST2
FLAG (RASSF1A)
GAPDH
E
MDA
H1299
pcDNA3
FLAG-RASSF1C
pcDNA3
FLAG-RASSF1C
pS127-YAP
YAP1
FLAG
GAPDH
F
IP:pY
IgG
Control
FLAG-RASSF1C
Control
FLAG-RASSF1C
YAP
IP:pY
Ratio
1.0 1.55 0.2 0.0
YAP
GAPDH
Lysates
G
RASSF1A Resist_1
RASSF1C Resist_1
RASSF1A Resist_2
RASSF1C Resist_2
siNT
siR1_1
siR1_2
siR1C
siNT
siNT
siNT
siR1_2
siNT
siR1_2
DAPI
pS127-YAP
YAP
Merge
H
siNT
siRASSF1_1
siRASSF1_2
siNT
FLAG-RASSF1AResist_1
FLAG-RASSF1AResist_2
FLAG-RASSF1CResist_1
FLAG-RASSF1CResist_2
FLAG (RASSF1A)
FLAG (RASSF1C)
GAPDH
siNT
siRASSF1
pcDNA
pcDNA
R1A*Res
R1C*Res
YAP
Fibrillarin
GAPDH
Nucleus
YAP
Fibrillarin
GAPDH
Cytoplasm
FLAG (RASSF1A*-Res)
FLAG-(RASSF1C*-Res)
GAPDH
Lysate

Figure S1 (Related to Figure 1). RASSF1C Leads to Nuclear Localisation of YAP1.

(*A*) Left, lysates from siNT, siRASSF1 or siRASSF1A treated U2OS cells western blotted for total YAP1 or pS127-YAP1. Images indicate vectors taken for determination of cytoplasmic (value = average of two vectors/cell, white arrows) nuclear (value = average of two vectors/cell, red arrows) staining and cell equatorial vector (yellow arrows). (*B*) Western blot of knockdown of exogenously expressed FLAG-RASSF1A (left) or FLAG-RASSF1C (right) by siRASSF1 or siRASSF1A. (*C*) Immunoblotting of lysates from U2OS cells transfected with siNT, siRASSF1 or siRASSF1A. (*D*) Immunoblotting of lysates from U2OS and H1299 cells transfected with either Control or Flag-RASSF1A. (*E*) Levels of total YAP1 and pS127-YAP1 in MDA-MB-231 (Left) and H1299 (Right) cells (RASSF1A ^{methyl}) in the presence and absence of FLAG-RASSF1C (*F*) Immunoprecipitation with total pY antibody from U2OS cells transfected with either Control or Flag-RASSF1C (Right). (*G*) Immunofluorescence images of YAP1 and pS127-YAP1 nuclear/cytoplasmic localisation of U2OS cells, transfected with siRNA resistant forms of RASSF1A and RASSF1C and treated with siNT, siRASSF1 (R1_1 or R1_2) or siRASSF1C (siR1C). Images represent siNT for R1_1 shown in Figure 1D. (H) Western blot showing RASSF1A and RASSF1C resistant construct expression is unaffected by siRNA treatment with siRASSF1_1 or siRASSF1_2 (Left). Nuclear/Cytoplasmic fractionation for YAP1 of U2OS cells transfected with either siNT or siRASSF1 and siRNA resistant versions of RASSF1A and RASSF1C (Right). All scale bars represent 20 μm.

Figure S2

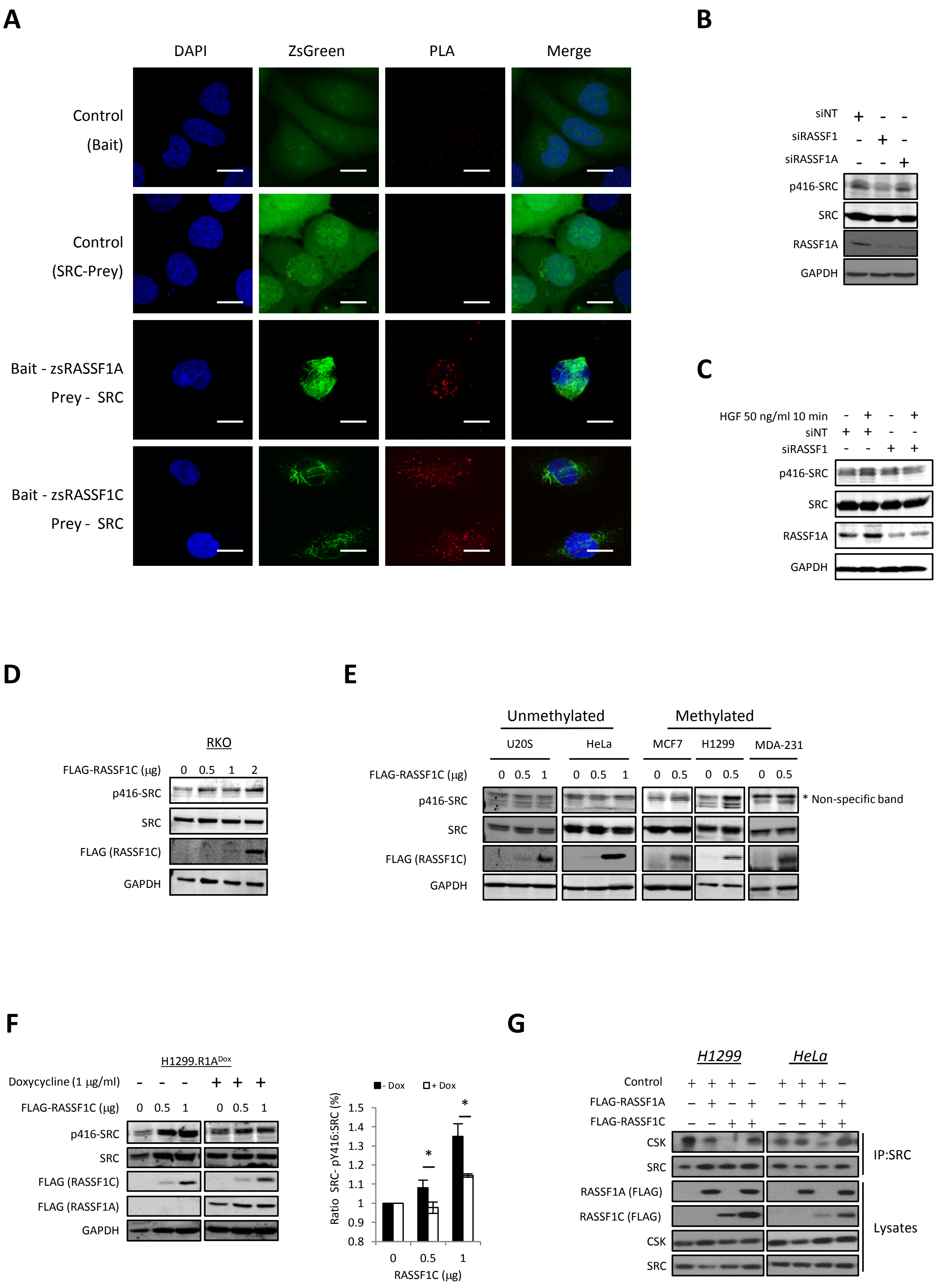
A
DAPI
ZsGreen
PLA
Merge
Control
(Bait)
Control
(SRC-Prey)
Bait - zsRASSF1A
Prey - SRC
Bait - zsRASSF1C
Prey - SRC
B
siNT + - -
siRASSF1 - + -
siRASSF1A - - +
p416-SRC
SRC
RASSF1A
GAPDH
C
HGF 50 ng/ml 10 min - + - +
siNT + + - -
siRASSF1 - - + +
p416-SRC
SRC
RASSF1A
GAPDH
D
RKO
FLAG-RASSF1C (µg) 0 0.5 1 2
p416-SRC
SRC
FLAG (RASSF1C)
GAPDH
E
Unmethylated
Methylated
U20S
HeLa
MCF7
H1299
MDA-231
FLAG-RASSF1C (µg) 0 0.5 1 0 0.5 1 0 0.5 0 0.5 0 0.5
p416-SRC
SRC
FLAG (RASSF1C)
GAPDH
* Non-specific band
F
H1299.R1A Dox
Doxycycline (1 µg/ml) - - - + + +
FLAG-RASSF1C (µg) 0 0.5 1 0 0.5 1
p416-SRC
SRC
FLAG (RASSF1C)
FLAG (RASSF1A)
GAPDH
- Dox
+ Dox
Ratio SRC- pY416:SRC (%)
1.5
1.4
1.3
1.2
1.1
1
0.9
0.8
*
*
0
0.5
1
RASSF1C (µg)
G
H1299
HeLa
Control + + + – + + + –
FLAG-RASSF1A – + – + – + – +
FLAG-RASSF1C – – + + – – + +
CSK
SRC
IP:SRC
RASSF1A (FLAG)
RASSF1C (FLAG)
CSK
SRC
Lysates

Figure S2 (Related to Figure 2) RASSF1C Expression Activates SRC Only When RASS1F1A is Absent. (*A*) Representative immunofluorescence images showing the different levels of colocalisation of RASSF1A and RASSF1C (Baits) with SRC (Preys) using proximity ligation assay (Duolink). (*B*) HeLa cells transfected with non-targeting siRNA (NT), siRNA targeting all RASSF1 isoforms (siRASSF1) or specifically RASSF1A (siRASSF1A). Blotted for SRC auto-phosphorylation site, Y416, using antibodies against avian SRC (p416-SRC). (*C*) Western blot of pY416-SRC in HeLa cells transfected with siNT or siRASSF1 prior to stimulation with 50 ng/ml HGF. (*D*) Western blot p416-SRC in RKO cells, methylated for RASSF1A, transfected with increasing concentrations of FLAG-RASSF1C. (*E*) Western blot of p416-SRC in RASSF1A positive (U2OS and HeLa) and RASSF1A negative (MCF7 and MDA-MB-231) cells transiently transfected with FLAG-RASSF1C. (*F*) H1299.R1A^{Dox} TET-ON FLAG-RASSF1A inducible cells transiently transfected with indicated concentrations of FLAG-RASSF1C and treated with doxycycline as indicated. Graph shows quantitation of SRC-pY416/SRC ratio. Error bars depict 1 x SEM. (*G*) Immunoprecipitation of SRC from lysates from H1299 and HeLa cells transfected with either Flag-RASSF1A or Flag-RASSF1C. All scale bars represent 20 μm.

Figure S3

A

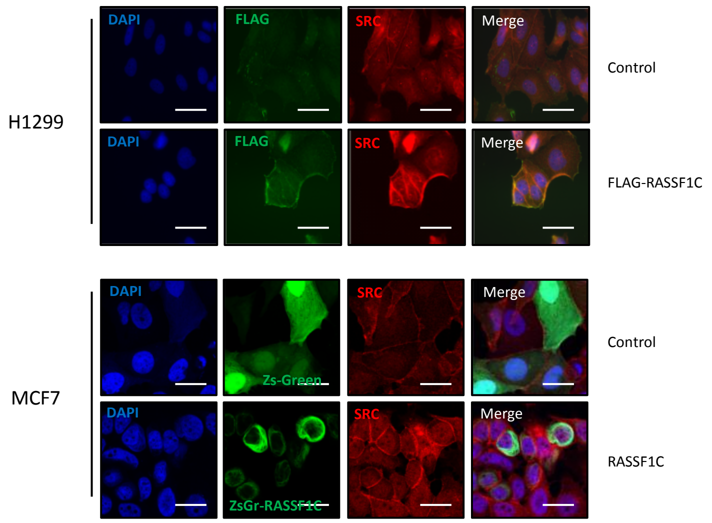

B

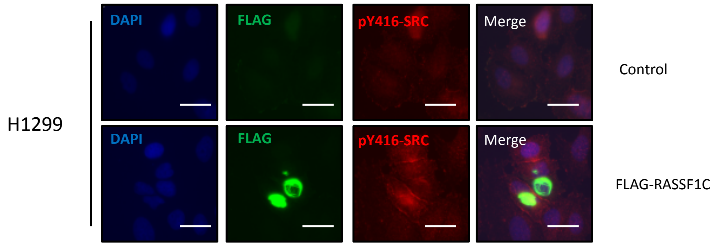

C

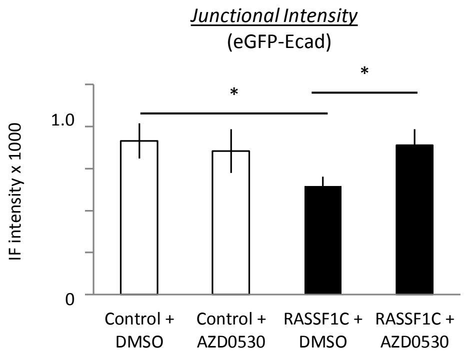

Figure S3 (Related to Figure 3). RASSF1C Localises SRC activation to Adherens Junction.

(*A*) Immunofluorescence images of SFK localisation in H1299 cells transfected with empty vector or FLAG-RASSF1C (top) and MCF7 cells transfected with Zs-Green empty vector or Zs-Green-RASSF1C (bottom). (*B*) Immunofluorescence images of pY416-SRC in H1299 cells transfected with empty vector or greater expression levels of FLAG-RASSF1C after 24hrs. (*C*) Junctional intensity of MCF7 cells transfected with eGFP-E-cadherin and either DsRed or DsRed-RASSF1C cells, treated with DMSO or AZD0530 (2.5 μM, 18 h). The intensity was measured using the Zeiss Zen 2011 software. All scale bars represent 20 μm.

Figure S4

A

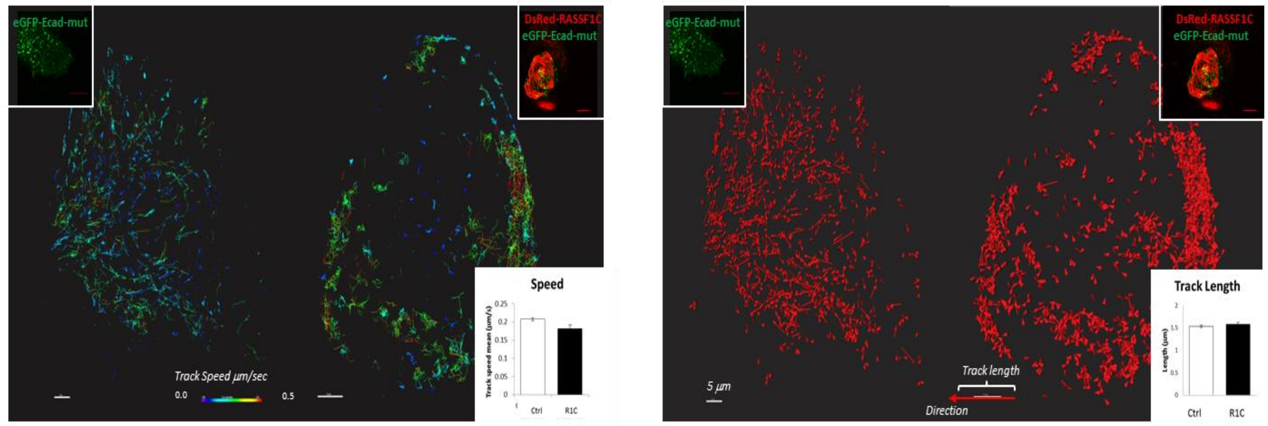
eGFP-Ecad-mut
DsRed-RASSF1C
eGFP-Ecad-mut
Speed
Track Speed µm/sec
0.0
0.5
Ctrl
R1C
eGFP-Ecad-mut
DsRed-RASSF1C
eGFP-Ecad-mut
Track Length
5 µm
Track length
Direction
Ctrl
R1C

B

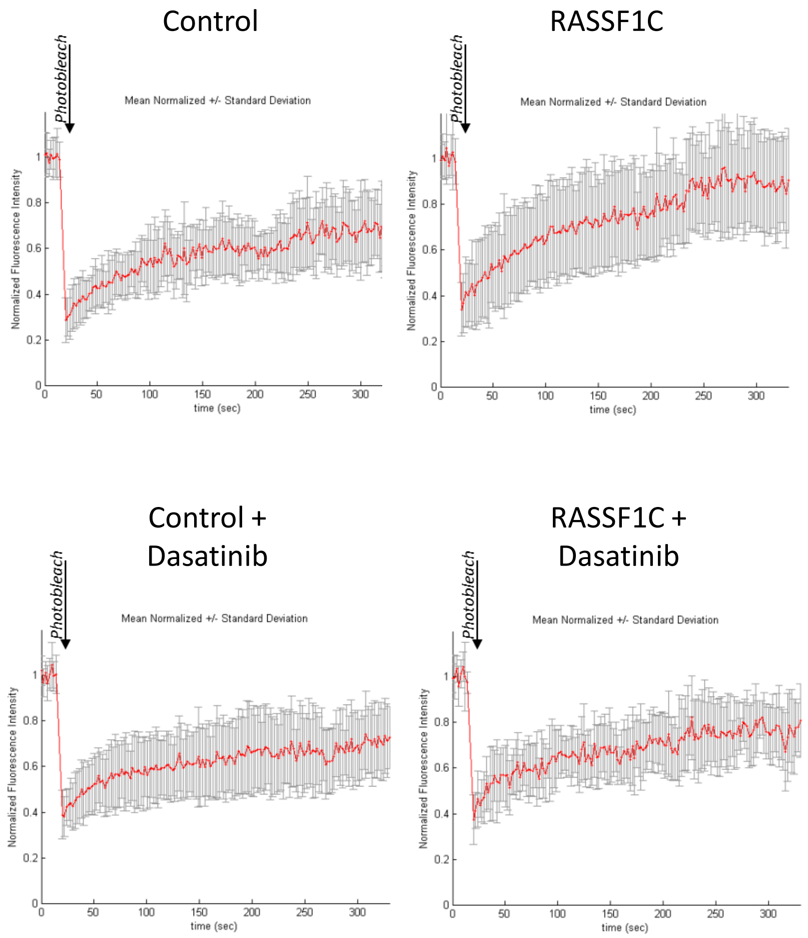
Control
RASSF1C
Control + Dasatinib
RASSF1C + Dasatinib
Photobleach
Mean Normalized +/- Standard Deviation
Normalized Fluorescence Intensity
time (sec)

Figure S4 (Related to Figure 4). Representative Graphs for the FRAP Analysis.

(*A*) Representative images of the tracking of all the vesicles in DsRed Control and DsRed-RASSF1C expressing MCF7 cells and transfected with eGFP-E-cadherin mutant (Y753F, Y754F, Y755F) (Imaris). Bar graphs show the analysis of the mean speed heatmap (Bottom left) or distance (Bottom right) of the vesicles in control and RASSF1C cells. For each analysis 5 cells per experiment were used and an average of 700 vesicles were tracked (bars). The results are from three independent experiments including Movie S2 and S3. Immunofluorescence images at the top demonstrate the accumulation of E-cadherin mutant and the expression of DsRed-RASSF1C. (*B*) Example graphs from FRAP analysis of junctional GFP-E-cadherin in MCF7 cells transfected with Ds-Red empty vector or Ds-Red-RASSF1C, treated with Dasatinib. Representative FRAP timelapse in Movies S4 and S5.

Figure S5

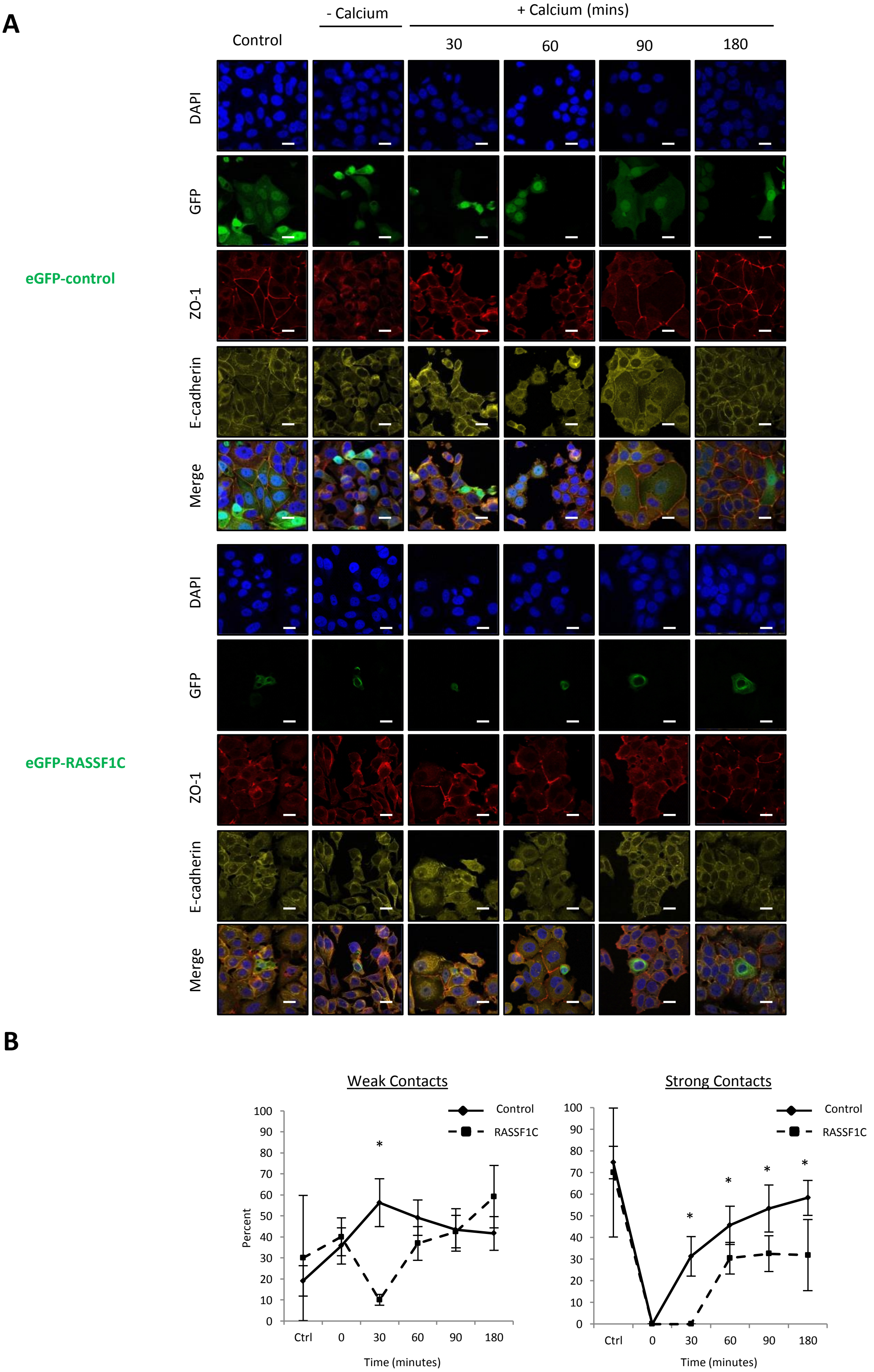
A
- Calcium
+ Calcium (mins)
Control
30
60
90
180
DAPI
GFP
ZO-1
E-cadherin
Merge
eGFP-control
eGFP-RASSF1C
B
Weak Contacts
Strong Contacts
Control
RASSF1C
Percent
Ctrl
0
30
60
90
180
Time (minutes)
*

Figure S5 (Related to Figure 4). RASSF1C Affects the Establishment of Cell-Cell Junctions.

(*A*) Example images of Calcium Switch Assay in MCF7 cells transfected with GFP-empty vector or GFP-RASSF1C. Control represents cells that have remained in media containing calcium. All scale bars represent 20 μm. (*B*) Quantitation of number of weak contacts (left) and strong contacts (right) (defined in methods) formed at indicated time points.

Figure S6

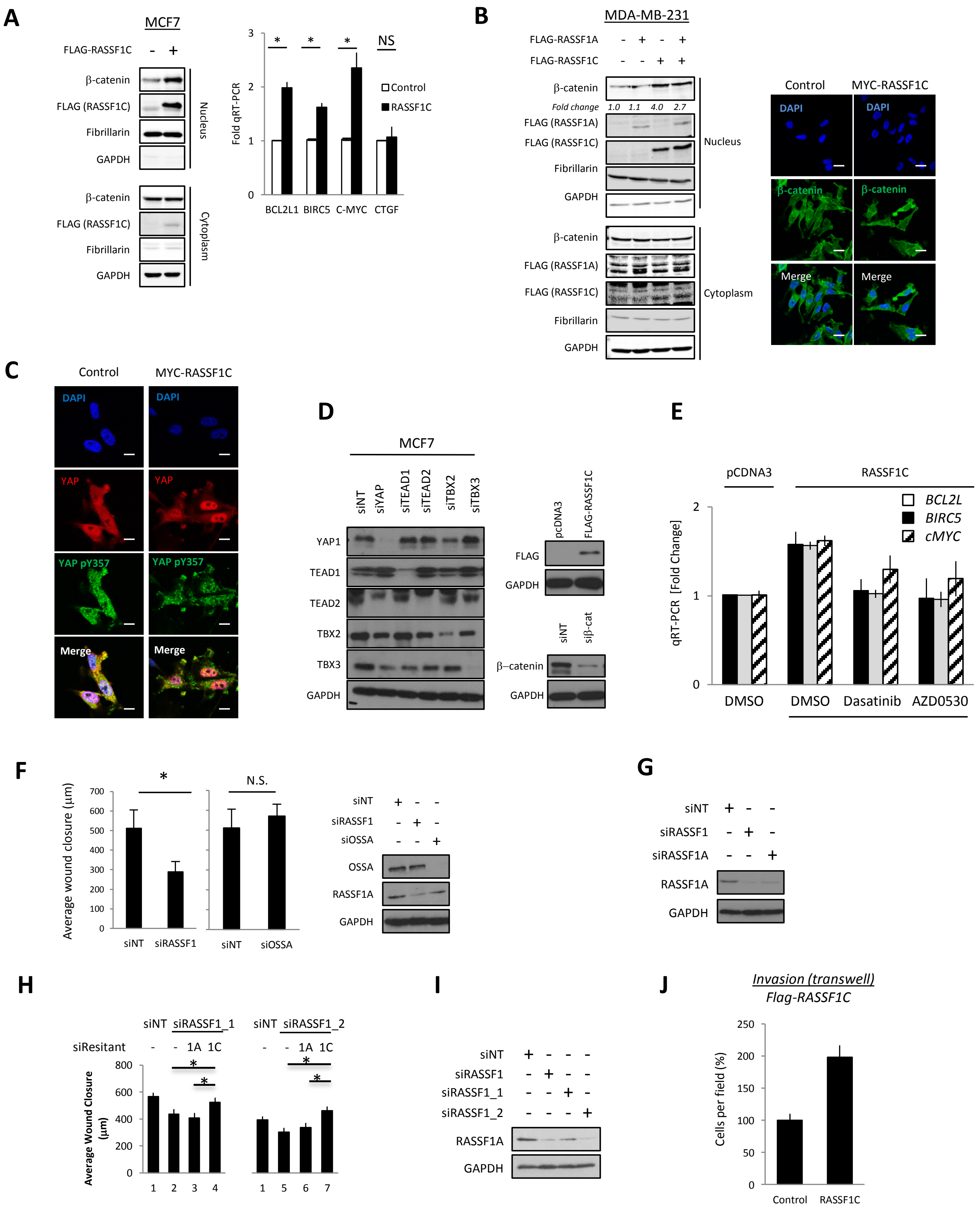

A
MCF7
FLAG-RASSF1C - +
β-catenin
FLAG (RASSF1C)
Fibrillarin
GAPDH
Nucleus
β-catenin
FLAG (RASSF1C)
Fibrillarin
GAPDH
Cytoplasm
Fold qRT-PCR
* * * NS
Control
RASSF1C
BCL2L1 BIRC5 C-MYC CTGF
B
MDA-MB-231
FLAG-RASSF1A - + - +
FLAG-RASSF1C - - + +
β-catenin
Fold change 1.0 1.1 4.0 2.7
FLAG (RASSF1A)
FLAG (RASSF1C)
Fibrillarin
GAPDH
Nucleus
β-catenin
FLAG (RASSF1A)
FLAG (RASSF1C)
Fibrillarin
GAPDH
Cytoplasm
Control
MYC-RASSF1C
DAPI
β-catenin
Merge
C
Control
MYC-RASSF1C
DAPI
YAP
YAP pY357
Merge
D
MCF7
siNT siYAP siTEAD1 siTEAD2 siTBX2 siTBX3
YAP1
TEAD1
TEAD2
TBX2
TBX3
GAPDH
pcDNA3 FLAG-RASSF1C
FLAG
GAPDH
siNT siβ-cat
β-catenin
GAPDH
E
pCDNA3
RASSF1C
BCL2L
BIRC5
cMYC
qRT-PCR [Fold Change]
DMSO DMSO Dasatinib AZD0530
F
Average wound closure (µm)
* N.S.
siNT siRASSF1 siNT siOSSA
siNT + - -
siRASSF1 - + -
siOSSA - - +
OSSA
RASSF1A
GAPDH
G
siNT + - -
siRASSF1 - + -
siRASSF1A - - +
RASSF1A
GAPDH
H
siNT siRASSF1_1 siNT siRASSF1_2
siResitant - - 1A 1C - - 1A 1C
Average Wound Closure (µm)
1 2 3 4 1 5 6 7
I
siNT + - - -
siRASSF1 - + - -
siRASSF1_1 - - + -
siRASSF1_2 - - - +
RASSF1A
GAPDH
J
Invasion (transwell)
Flag-RASSF1C
Cells per field (%)
Control RASSF1C

Figure S6 (Related to Figures 5 & 6). RASSF1C promotes β-catenin/YAP1 Dependent Transcription and Invasion.

(*A*) Nuclear/Cytoplasmic fractionation of MCF7 cells expressing empty vector or FLAG-RASSF1C (left). Quantification of qRT-PCR for YAP-β-catenin-TBX target genes BCL2L1 and BIRC5, β-catenin-TCF/LEF target gene c-MYC and YAP/TEAD target gene CTGF in MCF7 cells transfected with empty vector or FLAG-RASSF1C (Right). * represents $p < 0.05$. (*B*) Left: Nuclear/Cytoplasmic fractionation of MDA-MB-231 cells transiently transfected with empty vector, FLAG-RASSF1A, FLAG-RASSF1C or Both FLAG-RASSF1A and FLAG-RASSF1C to show β-catenin localisation, quantitated by Licor imaging software. Right: Immunofluorescence depicting β-catenin localisation in MDA-MB-231 cells transiently transfected with empty vector or MYC-RASSF1C. (*C*) Representative images for the localisation of YAP1 and pY357-YAP1 in MDA-MB-231 cells transfected with Control or MYC-RASSF1C vectors. All scale bars represent 20 μm. (D) Lysates from MCF7 cells with the indicated siRNAs used in qRT-PCR assays in Fig. 6B. (*E*) Quantification of qRT-PCR for BCL2L1, BIRC5 and c-MYC genes in MCF7 cells transfected with empty vector or FLAG-RASSF1C and treated with either Dasatinib (50 nM, 18 h) or AZD0530 (2.5 μM, 18 h). (*F*) Left: Quantification of scratch wound assay of siNT vs siRASSF1 (p= 2.6 x 10-4) and siNT vs siRNA targeting the oxidative stress-associated activator of SRC, OSSA (p= 0.16). Right: Western blot showing knock down of RASSF1A by siRASSF1 and OSSA by siOSSA. (*G*) Western blot of knockdown of RASSF1A by siRASSF1 and siRASSF1A, related to Fig. 3D. (*H*) Quantitation of scratch wound assay of HeLa cells transfected with siNT or two different siRNAs to RASSF1 (siRASSF1_1 or siRASSF1_2) and siRNA resistant constructs of either RASSF1A or RASSF1C as described in Supplementary Fig S1g, S1H (siRASSF1_1: siNT vs siRASSF1_1 p= 5.7 x 10-9, RASSF1C vs pcDNA p= 6.2 x 10-5, RASSF1C vs RASSF1A p= 9.7 x 10-10. siRASSF1_2: siNT vs siRASSF1_2 p= 1.9 x 10-6, RASSF1C vs pcDNA p= 5.4 x 10-12, RASSF1C vs RASSF1A p= 1.2 x 10-8). (*I*) Western blot showing knockdown of endogenous RASSF1A by siRASSF1 (SMARTpool), siRASSF1_1 and siRASSF1_2. (*J*) Quantification of transwell assay with MDA-MB-231 cells stably expressing empty vector or MYC-RASSF1C. Error bars depict 1 x SEM.

Figure S7

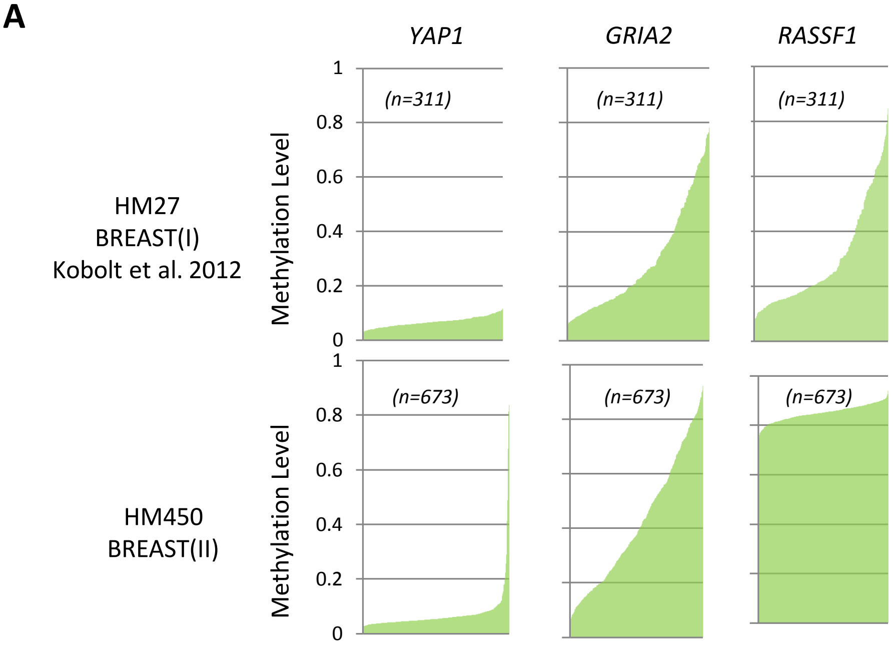
A
YAP1
GRIA2
RASSF1
(n=311)
(n=673)
Methylation Level
HM27
BREAST(I)
Kobolt et al. 2012
HM450
BREAST(II)
0
0.2
0.4
0.6
0.8
1

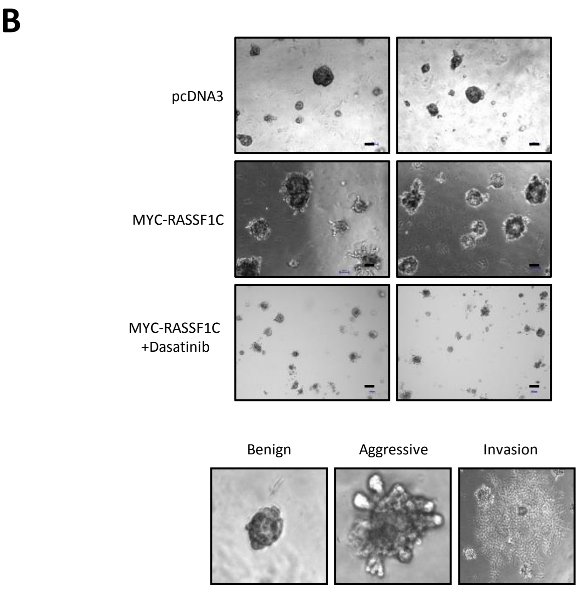
B
pcDNA3
MYC-RASSF1C
MYC-RASSF1C
+Dasatinib
Benign
Aggressive
Invasion

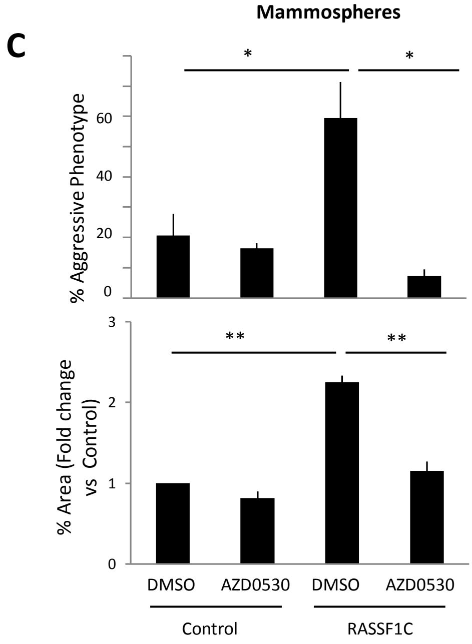
C
Mammospheres
% Aggressive Phenotype
% Area (Fold change vs Control)
0
20
40
60
1
2
3
*
**
DMSO
AZD0530
Control
RASSF1C

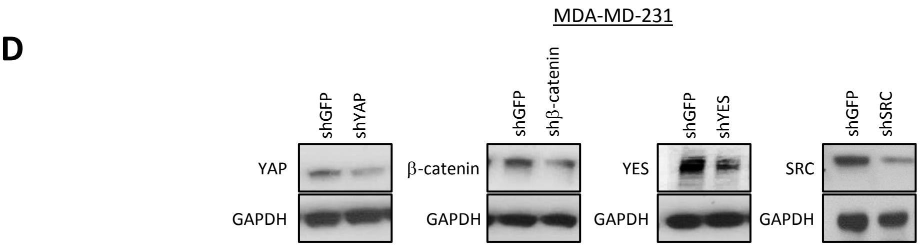
MDA-MD-231
D
shGFP
shYAP
shβ-catenin
shYES
shSRC
YAP
β-catenin
YES
SRC
GAPDH

Figure S7 (Related to Figure 7) RASSF1C Promotes Invasiveness *in vitro* and *in vivo*.

(*A*) Top: Invasive breast cancer dataset (BREAST (I)) methylation levels (n=311/825) for non-methylated (YAP1) and methylated genes (GRIA2 and RASSF1) using Illumina HM27 beadchi. Bottom: Invasive Breast cancer dataset (Breast (II)) analysed for the same targets using Illumina HM450 beadchip (n=673/1003), identifies similar levels of methylation as HM27 for *YAP1* and *GRIA2* but increased methylation of *RASSF1*. Data sets are non-overlapping and the same samples do not have information from both reads. (*B*) Example images of Mammospheres from MDA-MB-231 cells stably expressing empty vector, MYC-RASSF1C or MYC-RASSF1C treated with dasatinib grown in matrigel. Aggressive phenotype was defined as mammospheres with greater than 5 invasive projections into the surrounding matrigel. Comparative images of benign, aggressive and invasion (invasion of MDA-MB-231 cells stably expressing RASSF1C through matrigel to the base of the tissue culture dish) are also indicated. All scale bars represent 100 μm. (*C*) Mammospheres grown in matrigel from MDA-MB-231 cells stably expressing empty vector or MYC-RASSF1C were treated on days 3, 6 and 9 with either DMSO or SFK inhibitor (1.25 μM AZD0530). Images were taken on day 10. Graphs indicate the number of mammospheres with aggressive phenotypes (Top) and the size of mammospheres (Bottom). (*D*) Immunoblots showing the knockdown of the targeted genes by shRNA in MDA-MB-231 cells used for mammospheres from Figure 7D.

Table S1

Gene expression signature	*Significance threshold Mann-Whitney (Bonferroni corrected)	Gene (n)	% Signature Genes with variation in mRNA levels* (expected direction)									% Signature genes increased (^) and decreased (v) in <pS127 or <YAP		
			Glioma			Bladder			Breast (I)			Breast (II)		
			< pS127	< YAP1	Fisher's exact (pS127 vs YAP1)	< pS127	< YAP1	Fisher's exact (pS127 vs YAP1)	< pS127	< YAP1	Fisher's exact (pS127 vs YAP1)	< pS127 (Fisher's exact) ^ vs v	< YAP1 (Fisher's exact) ^ vs v	Fisher's exact ($pS127^{low}$ vs $YAP1^{low}$)
Invasive breast cancer (POOLA)	0.0001	275	32%*	25%*	*0.07*	0.4%	6%	***p*=0.0001**	2%	0.4%	*p*=0.1	^ 15% v 9% *p=0.036*	2.5% 4% *p=0.35*	***p*=1.09e^{-07}**
Metastasis (BIDUS)	0.0004	214	17%	4%	*p=0.2*	0	0%	*p*=1	11%	2%	***p*=0.006**	^ 25% v 1% *p=2.77e^{-15}*	3% 1% *p*=0.2	***p*=4.52e^{-11}**
Cancer EMT (ANASTASSIOU)	0.0007	64	17%	9%	*p*=0.3	2%	53%	***p*=6.52e^{-12}**	5%	11%	*p*=0.3	^ 89% v 0% *p=2.2e^{-16}*	42% 0% *p*=4.5e^{-10}	***p*=2.62e^{-08}**
YAP SIGNATURE (CORDENONSI)	0.0008	57	25%	26%	*p=1*	2%	14%	*p=0.03*	10.5%	25%	*0.08*	^ 38% v 5% *p=2.24e^{-05}*	28% 2% *p*=9.3e^{-05}	*p=0.3*
REACTOME_YAP_TAZ	0.002	24	17%	17%	*p*=1.0	4%	8%	*p*=1.0	25%	20%	*p=1*	^ 33% v 0% *p=3.90e^{-03}*	25% 0% *p*=0.04792	*p=0.7*
Alzheimer's	0.0001	388	-	-	-	-	-	-	-	-	-	^ 2% v 7% *p=0.0011*	0.5% 7% *p*=1.44e^{-07}	***p=0.6***

* opposite direction

Table S1 (Related to Figure 7A). Enrichment analysis of mSigDB invasive signatures in Cohorts of Glioma, Bladder and Breast (I – Koboldt et al. 2012; II - Invasive Carcinoma HM450) from The Cancer Genome Atlas (TCGA). Cohorts were separated into groups of high and low based on the top and bottom 100 zScores for phospho-S127-YAP1 or total YAP1. The number of genes that increased above the significance cut off for each signature (*) in the low group were scored as a percentage. Comparison of changes in pS127-YAP1 to YAP1 indicates specificity for loss of the phospho signal rather than effects of total protein – confirmed by indistinguishable scores for YAP-TAZ signatures. For Breast (II) we additionally show percentage of signatures genes that go in the opposite direction, genes decreased in low groups (v).

Table S2

Gene Signature	Breast (II) vs.	pS127-YAP1 Fisher's exact	pS127-YAP1 OR	YAP1 Fisher's exact	YAP1 OR
POOLA	Breast (I)	$2.85e^{-08}$	8.05	0.07	7.14
	Glioma	**$5.32e^{-06}$**	0.38	**$2.43e^{-15}$**	0.08
	Bladder	$1.98e^{-12}$	49.13	0.06	0.40
BIDUS	Breast (I)	$8.15e^{-08}$	5.07	0.17	3.57
	Glioma	$5.65e^{-07}$	4.35	1	0.87
	Bladder	$< 2.2e^{-16}$	Inf	0.01	Inf
ANASTASSIOU	Breast (I)	$< 2.2e^{-16}$	151.34	$5.04e^{-05}$	6.24
	Glioma	$< 2.2e^{-16}$	37.42	0.14	1.83
	Bladder	$< 2.2e^{-16}$	456.63	1	0.97
YAP/TAZ (REACTOME)	Breast (I)	0.75	1.49	0.74	1.55
	Glioma	0.32	2.45	0.49	2.03
	Bladder	0.02	10.98	0.14	4.39
YAP (CORDENONSI)	Breast (I)	0.0009	5.26	0.83	1.20
	Glioma	0.16	1.92	1	1.09
	Bladder	$5.61e^{-07}$	34.32	0.10	4.39

Table S2 (Related to Figure 7A). Low levels of pS127-YAP1 correlate with invasive signature only in the context of *RASSF1-1*α methylation.

Comparison of invasive gene signatures enrichment (mSigDB database) in Breast II - Invasive Carcinoma HM450 to BREAST (I), Glioma and Bladder of The Cancer Genome Atlas (TCGA). Elevated YAP-TAZ signature genes are equivalent for pS127-YAP1 and YAP1 in all cohorts (Supplementary Table 1) and no significant difference is observed between Breast I and II, however, invasive, metastatic and EMT signatures show highly significant differences between the *RASSF1-1*α methylated Breast II and limited methylated Breast I. Similar significance is observed with Glioma and Bladder where identical HM450 analysis was applied to characterise *RASSF1-1*α .

Supplemental Experimental Procedures

Cell Culture and Transfection.

Cells were purchased from ATCC and maintained in DMEM supplemented with 10 % FBS, 2 mM Glutamine and 100 U/ml Penicillin/Streptomycin (all from Life Technologies) at 37 ^{o}C, 5 % CO_2 in a humidified incubator. Cells were passaged by trypsinising (0.25 % Trypsin EDTA solution (Life Technologies) and reseeding when necessary. Cells were transfected using Lipofectamine 2000 transfection reagent (Life Technologies) according to manufacturer's guidelines. See siRNA sequences table at the end of Experimental Procedures. For experiments investigating SRC activation by RASSF1C, or using growth factors (HGF (Peprotech)), cells were incubated overnight in DMEM containing 0.1 % FBS to remove growth factor stimulation prior to growth factor treatment and cell lysis.

Cell Lysis.

Cells were lysed for western blot using Laemmli lysis buffer (2.5 mM Tris-HCl pH6.8, 2 % SDS, supplemented with protease inhibitor) before boiling (100 ^{o}C, 10 min). Protein concentration of lysates for western blot and prior to addition of lysate to immunoprecipitations was done using Bradford reagent. Absorbance was analysed using POLARstar OMEGA machine at 595 nm.

Mammosphere Growth Assay.

24 well plates were coated with a thick layer (300 μl per well) of matrigel mixed 1:1 with DMEM media without supplements. Matrigel was allowed to set for at least 30 min at 37 ^{o}C before 2500 MDA-MB-231 cells were plated on top of the matrigel. Mammospheres were allowed to form over 10 days changing the media every 3 days. Images of the mammospheres were taken at 4 X magnification with Nikon TE2000 Eclipse microscope using NIS elements software. Image analysis was done using Image J software.

Nuclear/Cytoplasmic Fractionation.

Cells were trypsinised, collected, washed two times with PBS and incubated for 15 min on ice in cytoplasmic lysis buffer (10 mM HEPES pH 7.9, 10 mM KCl, 0.1 mM EDTA, 0.1 mM EGTA, 1 mM DTT, 0.5 mM PMSF) before NP40 detergent was added (final concentration = 0.65 % (v/v)). Cells were vortexed immediately for 10 seconds and centrifuged (1500 x g, 5 min, 4 ^{o}C). The supernatant (cytoplasmic fraction) was removed and placed into a fresh tube. The pellet (Nuclear fraction) was further lysed with Laemmli lysis buffer (see Cell Lysis). Lysates were analysed by western blot.

Immunoprecipitation.

Cells were lysed in immunoprecipitation lysis buffer (150 mM NaCl, 1 % NP40, 20 mM Hepes pH 7.5, 0.5 mM EDTA, 1 x protease inhibitor (Roche), 50 mM NaF, 10 mM β–glycerophosphate and 0.5 mM Sodium orthovanidate). Lysates were then cleared by centrifugation (20817 x g, 10 min, 4 °C). The protein concentration of the lysates was then determined by Bradford assay, such that an equal amount of protein was loaded into each immunoprecipitation. Protein G Dyna-beads (Millipore) were washed three times before indicated antibodies and lysate were added to beads and rotated at 4 °C for 3 hr. Beads were washed four times with immunoprecipitation wash buffer (150 mM NaCl, 1 % NP40, 20 mM Hepes pH 7.5, 0.5 mM EDTA) by centrifugation (2150 x g, 4 °C, 2 min) prior to resuspension in loading buffer (10 % Glycerol, 62.5 mM Tris-HCl pH 6.8, 2 % SDS, 2 % 2-Mercaptoethanol plus bromophenol blue).

Immunoprecipitation of Purified Proteins.

Recombinant His-tagged RASSF1A (1 μg) (Fitzgerald Industries, USA) was mixed with either recombinant, kinase active His-tagged CSK (1 μg) (Active Motif) or His-tagged SRC (1 μg) (Active Motif) in a reaction tube with mild lysis buffer (0.1% NP40, 20 mM HEPES pH 7.5, 150 mM NaCl, 0.5 mM EDTA, 50 mM NaF, 10 mM β–glycerophosphate and 0.5 mM Sodium orthovanidate) supplemented with protease inhibitors (Roche) in 20 μl volume. The tubes were then incubated for 30 minutes at 37 °C after which 5 μl of each reaction was used for immunoprecipitation. The pull-downs were achieved with either SRC (Cell Signalling) or RASSF1 (Santa Cruz) antibodies bound to agarose beads for 1.5 hours at 4 °C under constant rotation. The beads were washed four times with the lysis buffer by centrifugation (2150 x g, 4 °C, 5 min) prior to resuspension in loading buffer (10 % Glycerol, 62.5 mM Tris-HCl pH 6.8, 2 % SDS, 2 % 2-Mercaptoethanol plus bromophenol blue).

Immunofluorescence.

Cells, plated onto glass cover slips, were fixed for 15 min in 4 % paraformaldehyde (PFA) and permeabilsed with 0.2 % Triton X before blocking with 0.2 % Fish Skin Gelatin (FSG) for 1 hr. Primary antibody incubations were carried out at 4 °C overnight in 0.2 % FSG. Unless otherwise stated primary antibodies were diluted 1 in 100. Cover slips were washed three times with PBS before incubation with Alexa fluor secondary antibody (1 in 500) (Life Technologies). Cover slips were washed a further three times with PBS before being mounted onto slides with ProLong Gold antifade mounting medium with DAPI (Life Technologies). Slides were imaged using Nikon 90i microscope using NIS elements software, Leica DM IRBE microscope using Simple PCI6 software or Zeiss LSM780 confocal microscope using Zeiss ZEN2011 software.

Dispase Assay.

Cells were incubated in 2 μg/ml Dispase II solution (Sigma) for 30 min before junctions were disrupted mechanically by pipetting. Cells were then centrifuged (1 x g, 5 min) to pellet cell aggregates. Samples were taken from the top of the supernatant and analysed using a haemocytometer.

Scratch Wound Assay.

Cells were grown at high confluency on a tissue culture plate. A wound was made using a P200 pipette tip. Dislodged cells were removed by washing 3 times with PBS before media was re-added to the plate. HeLa cells were incubated in DMEM supplemented with 10 % serum for 24 hr. MDA-MB-231 cells were incubated in DMEM supplemented with 1 % serum for 14 hr. Images were taken at the same point in each plate at 0 hr and the end time point using the Nikon TE2000 Eclipse microscope and analyzed using NIS elements software. Transwell assay is described in supplementary methods.

Transwell Assay.

DMEM media containing 10 % FBS was loaded into the bottom chamber of a transwell plate (BD-Biosciences) before 2.5 x 10^4 MDA-MB-231 cells were loaded into the top chamber of a transwell plate in DMEM containing 0.1 % FBS. Cells were allowed to migrate at 37 ^oC, 5 % CO_2 for 18 hr in a humidified incubator before cells were fixed in 100 % methanol for 10 min at RT. Transwell membranes were removed from the bottom of the top chamber and mounted onto microscope slides using Prolong® Gold antifade reagent with DAPI (Life Technologies). Cells that had migrated through the membrane were then counted using a Nikon 90i microscope at 20 X magnification. Five fields of view were counted per membrane and three membranes were used in each experiment.

Calcium Switch Assay.

MCF7 cells were grown at high density (3 x 10^5 cells/condition) on cover slips in calcium containing media. With the exception of 'Control', cells were washed twice with PBS before being incubated overnight in calcium free media (Life Technologies). The cells were then washed in PBS and normal media (DMEM supplemented with 10 % FBS, 2 mM Glutamine and 100 U/ml Penicillin/Streptomycin) was added for up to 180 minutes. Coverslips were collected at the indicated time points. The coverslips were then stained using the immunofluorescence protocol. Images were taken using Ziess LSM780 using a 63 X objective and analysed using ZEN software. Cells expressing GFP-empty vector or GFP-RASSF1C were utilised for single cell analysis. Total contacts were calculated as the percentage of junctions formed vs the total number of cells in contact with cell being analysed.

Weak contacts were defined as being diffuse or interrupted staining at the cell-cell junction. Strong contacts were defined as robust, well defined staining at the cell-cell junction.

Mass Spectrometry.

Gel pieces excised from the SDS-PAGE gel were subjected to tryptic digest and the resulting peptides extracted from the gel following the *in-gel* digest protocol from the Kessler lab (www.ccmp.ox.ac.uk/protocols-and-tools). Briefly, samples were incubated overnight at RT in wash buffer (50 % (v/v) methanol, 5 % (v/v) acetic acid) before dehydration with acetonitrile. Samples were reduced (10 mM DTT, 10 min, RT) and alkylated (50 mM iodoacetamide, 10 min, RT) prior to digestion overnight at 37 ^{o}C with 20 μg/μl trypsin (Promega) (in 50 mM ammonium bicarbonate). Excess trypsin was removed and gel pieces incubated at 37 ^{o}C overnight. Trypsinised protein was extracted from the gel with extraction buffer 1 (50 % (v/v) acetonitrile, 5 % (v/v) formic acid, 10 min, RT) followed by extraction buffer 2 (85 % (v/v) acetonitrile, 5 % (v/v) formic acid, 10 min RT). Extracted peptides were analysed by online nanoflow liquid chromatography tandem mass spectrometry using a Dionex U300 (fitted with a Pepmap C18 column and eluted with a linear gradient of acetonitrile) connected to a Bruker HCTultra ETD II ion trap trough a nanoelectrospray ion source. Identified peptides were analysed using Mascot MS/MS ion search (www.matrixscience.com).

ExCELLigence Analysis.

160 μl DMEM containing 10 % FBS, with or without doxycycline (2 μg/ml) (SIGMA) were added to the bottom chamber of an exCELLigence CIM plate. The top chamber was then attached to the bottom chamber and 50 μl of DMEM without supplements was added to each well. The plate was loaded into the exCELLigence analyser and a blank run was done to zero the machine. 40,000 cells were plated into the top chamber and allowed to settle for 30 min at room temperature before the plate was loaded into the exCELLigence analyser. Cells were incubated in the machine at 37 ^{o}C, 5 % CO_2 in a humidified incubator and measurements were taken every 15 min for 25 hr. [S11]

Quantitative real time PCR (qPCR).

50,000 cells were lysed and cDNA prepared using the Power SYBR Green Cells-to-Ct Kit (Life Technologies). qPCR was done using the same kit on an Applied Biosystems 7500 Fast Real Time PCR system. Protocol: Holding Step (1 x cycle: 95 ^{o}C 10 min), Cycling Step (50 x cycle: 95 ^{o}C 15 sec, 60 ^{o}C 1 min) and Melt Curve Step (1 x cycle: 95 ^{o}C 15 sec, 60 ^{o}C 1 min, 95 ^{o}C 30 sec, 60 ^{o}C 15 sec). 18S was used as an internal control. Primers are listed in Table 1.

Western Blot.

Protein samples were loaded onto a gel and separated by SDS-PAGE. NuPAGE® precast gels (10 % or 4-12 %) (Life Technologies) were loaded into a Novex® mini cell tank filled with Novex® MOPS running buffer (both from Life Technologies). Self-cast gels made with a 10 % resolving gel and 4 % stacking gel and were loaded into BioRad Mini Protean® TetraCell gel tanks. Protein was transferred onto PVDF-F or PVDF-P using Bio-Rad mini gel blotting system. PVDF-P was blocked and incubated with primary with 5 % non-fat milk or BSA diluted in PBS-Tween 20. Secondary antibodies were always incubated in 5 % non-fat Milk. PVDF-P membranes were covered in ECL solutions from Thermo Scientific, Millipore or GE Healthcare prior to exposure to film (Kodak) and developed in a XoGraph developer. PVDF-F membranes were blocked and incubated with both primary and secondary antibodies in Licor blocking buffer (diluted 1:1 in PBS) prior to being analysed using the Licor Odyssey analyser. Membranes were quantitated using Image J or Licor Odyssey software. Primary antibodies: pLATS1 (Ser909), pLATS1 (Thr1079), YAP-pS127, phospho-MST1 (T183)/MST2 (T180), YES, SRC-pY416, SRC-pY527, SFK, c-SRC, E-cadherin and CSK were purchased from Cell Signalling Technologies; RASSF1A (3f3), TBX3 (A-20), TEAD2, β-catenin, YAP and CSK were purchased from Santa Cruz Biotechnology; TBX2 (62-2) (provided by Colin Goding); LATS1 (BETHYL Labs). RASSF1C, YAP-pY357, Fibrillarin and FAM120A (OSSA) were purchased from Abcam; FLAG-(M2)-tag, MYC-tag, HA-tag and anti-pTyrosine were purchased from Millipore, GAPDH and MST2 were purchased from Epitomics; p120-catenin, FAK and TEAD1were purchased from BD Biosciences; ZO-1 from Invitrogen.

Animal experiments.

Female SCID mice, 7-8 weeks old, were anaesthetized with 2-3 % isoflurane in 70 % N_2O:30 % O_2, placed in a stereotaxic frame (Stoetling Co., USA) and maintained with 1 % isoflurane. The skull was exposed and a burr-hole drilled. Three groups of animals were focally injected with either 5 x 10^3 MDA-MB-231 tumor cells untreated, expressing empty vector (pCDNA3) or MYC-RASSF1C, in 0.5 µl PBS in the left striatum (coordinates relative to Bregma; anterior +0.5 mm, lateral 1.5 mm, depth 2.5 mm) using a 75 mm-tipped glass microcapillary (Clark Electromedical Instruments, UK). At day 21 after intracerebral tumor cell injection all animals were transcardially perfusion-fixed under terminal anesthesia (n = 4 per group) with 0.9 % heparinized saline followed by 200 ml of periodate lysine paraformaldehyde (PLP) containing only 0.025 % glutaraldehyde (PLP*light*). The brains were post-fixed, cryoprotected, embedded and frozen in isopentane at -40 °C. Immunohistochemistry. For immunohistochemical analysis, 10 µm sections were collected onto gelatinized slides, washed in PBS

and counterstained for 5 min in cresyl violet. Slides were mounted and mounted using DPX (Thermo Fisher Scientific, UK). To assess areas of tumor colonization, photomicrographs of each brain section were obtained using ScanScope CS slide scanner (Aperio, Vista, CA, USA) and analyzed using ImageScope (Aperio). For immunofluorescence, sections were quenched with 1 % hydrogen peroxide in PBS, streptavidinand biotin-blocked (SP-2002, Vector Laboratories), blocked (TNB, PerkinElmer), incubated with anti-CD34 primary antibody (Abcam, UK, brain vessels) and anti-Vimentin antibody (VectorLabs, tumor cells), and secondary antibody (biotinylated anti-Rat and anti-Rabbit antibodies respectively). Sections were then washed with PBS, incubated with streptavidin-HRP (PerkinElmer; 1:200) in TNB for 30 min, washed and incubated for 8 min in the dark with TSA-biotin (PerkinElmer; 1:100) in amplification buffer (PerkinElmer). Slides were washed and incubated with a streptavidin-Cy3 fluorophore or AMCA-conjugated secondary antibody (Invitrogen; 1:100) for 30 min. Slides were cover-slipped using Vectashield mounting medium (Vector Laboratories).

RT-PCR primer sequences.

Target Gene	Forward Primer	Reverse Primer
BCL2L1[16]	ACTCTTCCGGGATGGGGTAA	ACAAAAGTATCCCAGCCGCC
BIRC5[16]	TGACGACCCCATAGAGGAACA	CGCACTTTCTCCGCAGTTTC
C-MYC[16]	CCTACCCTCTCAACGACAGC	CTTGTTCCTCCTCAGAGTCGC
CTGF	CACCCGGGTTACCAATGACA	GGATGCACTTTTTGCCCTTCTTA
RASSF1A	AGTGCGCGCATTGCAAGTT	AAAGGTCAGGTGTCTCCCAC
RASSF1C[26]	CTGCAGCCAAGAGGACTCGG	GGGTGGCTTCTTGCTGGAGGG
18S[30]	AGTCCCTGCCCTTTGTACACA	GATCCGAGGGCCTCACTAAAC

siRNA sequences.

Target Gene	siRNA target sequence	Purchased from
siRASSF1 (siGenomeSMARTpool)	ACGCACAAGGGCACGUGAA	Thermo (Dharmacon)
	CAAGGACGGUUCUUACACA	
	GCAAGAAGCCACCCUCCUU	
	CUACAUAACUUCCUACGUA	
siRASSF1A	GACCUCUGUGGCGACUUC	Eurofins MWG
siMST	GGAUAGUUUUUCAAAUAGGtt	Ambion
siOSSA (siGenome SMARTpool)	GCGUAUGACUCUGAUUAUG	Thermo (Dharmacon)
	GUUAUUCGAUUUAAGAGAG	
	AGGCAGCUGUCUUAAAUAA	
	GCUAUCAGCUCUCUUCAUG	
siRASSF1_1	ACGCACAAGGGCACGUGAA	Eurofins MWG
siRASSF1_2	CAAGGACGGUUCUUACACA	Eurofins MWG
siYAP1	CUGGUCAGAGAUACUUCUUtt	
siCTNNB1 (siGenome SMARTpool)	GCUGAAACAUGCAGUUGUA	Thermo (Dharmacon)
(b-catenin)	GAUAAAGGCUACUGUUGGA	
	CCACUAAUGUCCAGCGUUU	
	ACAAGUAGCUGAUAUUGAU	
siTEAD1 (siGenome SMARTpool)	Cat no. GS7003	Qiagen
siTEAD2 (siGenome SMARTpool)	GGAAGACCCGAACUCGAAA	Thermo (Dharmacon)
	GGAAUGAACUGAUCGCCCG	
	GCAGUUGAUUCUUACCAGA	
	CGAAGGAAAUCAAGGGAAA	
siTBX2	GUUUCACAACUCCCGCUGGUU	
siTBX3	CAGCTCACCCTGCAGTCCA	
siNT siGenome sequence No. 2	siGenome sequence No. 2	Thermo(Dharmacon)
siYES1	GAAGGACCCUGAUGAAAGA	Thermo(Dharmacon)
siSRC	GAGAACCUGGUGUGCAAAG	Thermo(Dharmacon)

Supplemental References

S1. Scrace, S., O'Neill, E., Hammond, E.M., and Pires, I.M. (2013). Use of the xCELLigence System for Real-Time Analysis of Changes in Cellular Motility and Adhesion in Physiological Conditions. Methods Mol Biol *1046*, 295-306.
